# Deterministic wet etching of aspheric fiber microlenses with tunable conic geometry for tailored optical functionality

**DOI:** 10.1038/s41467-026-75331-y

**Published:** 2026-08-07

**Authors:** Sanggon Kim, Iason Keramidis, Isabel Plasencia-Fernandez, Johanna Alonso, Louison Brochoire, Cyril Bories, Annie Barbeau, Younes Messaddeq, Yves De Koninck

**Affiliations:** 1 Center for Optics, Photonics and Lasers (COPL), Department of Physics, Université Laval, Québec, QC Canada; 2 CERVO Brain Research Centre, Quebec Mental Health Institute, Québec, QC Canada; 3 Departments of Psychiatry & Neuroscience and Anesthesiology & Intensive Care, Université Laval, Québec, QC Canada

**Keywords:** Fibre optics and optical communications, Design, synthesis and processing, Optical manipulation and tweezers, Biophotonics

## Abstract

Efficient coupling between guided optical fiber modes and radiated fields in the surrounding medium remains a fundamental limitation across photonics, sensing, and biophotonics. Micro-lensed fibers offer a promising solution, yet scalable fabrication with predictable geometry and deterministic optical performance has remained elusive. Here, we introduce laser-controlled wet-chemical etching (LCWCE), a single-parameter strategy that directly sculpts micro-lenses, from hyperbolic to parabolic and prolate elliptical profiles, on standard optical fibers. Local laser illumination establishes an axially confined etching-rate gradient, enabling sub-micrometer control of curvature and working distance independent of fiber type or internal structure. A physics-separated multiphysics framework combining wave optics, heat-transfer, and temperature-dependent etching kinetics captures the observed geometry evolution and validates the underlying mechanism. LCWCE enables milliwatt-scale, minimal-power fiber-based optical trapping, as well as minimally invasive in vivo dendritic detection and single-cell neural interrogation beyond 1.5 mm depth in live brains, transforming ubiquitous optical fibers into scalable, high-performance photonic probes.

## Abstract (web summary)

Fabricating microscale optical structures with precise and predictable geometry remains a longstanding challenge in photonics. Here, the authors demonstrate a laser-controlled chemical etching approach for deterministic formation of aspheric fiber microlenses with programmable geometry and optical functionality.

## Introduction

Fiber optics is at the forefront of modern technology, driving advancements in high-speed data transmission^1^, remote and precise sensing^2,3^, and cutting-edge medical diagnostics^4–7^. Silica-based fibers are especially valued for their high transmission efficiency and low background noise. Yet, despite their intrinsic advantages, efficient light coupling, especially with precise matching of the fiber’s guided modes, remains a persistent challenge limiting system performance.

Microstructuring fiber tips has emerged as a powerful way to address this challenge, enhancing coupling efficiency and expanding functional versatility^6,8–11^. In particular, micro-lensed fibers (MLFs), microscale convex lenses integrated directly on the fiber tip^11^, offer compact, alignment-free coupling with broadened acceptance angles, improved mode matching, and reduced system bulk. The versatility of MLFs is reflected in their diverse applications, which span: laser diode coupling^11^, photonic devices, integrated circuits^11–13^, quantum optics^14^, high-resolution microscopy^15,16^, mass spectroscopy^17,18^, laser micro-machining^19^, coherence tomography^16,20^, optical tweezers^21^, bio/chemical sensing^22–25^, endoscopy^26^, and minimally invasive surgery^27^. This breadth highlights the far-reaching significance of MLFs across photonics, life sciences, and precision engineering.

Despite this promise, MLF adoption has been hindered for decades by fabrication bottlenecks. Existing methods, including thermal pulling and breaking^28–30^, laser micromachining^31^, arc processing^32^, and mechanical grinding^33,34^, struggle with precision, reproducibility, scalability, or cost. Melting-based approaches inherently form spherical rather than aspheric structures, compromising beam shaping and coupling efficiency^35,36^. Grinding allows unique geometries^33,34^ but is labor-intensive, rough-surfaced, and unsuited for high-throughput production^37^. While focused ion beam (FIB) milling can achieve extremely high geometric precision^38^, its prohibitive cost, low throughput, and reliance on specialized facilities severely limit its scalability and practical adoption. More recently, Two-photon lithography (TPL) has emerged as a versatile alternative for fabricating complex microlens (ML) geometries with improved economic accessibility^39,40^. However, TPL inherently relies on polymer-based photoresists, introducing a glass–polymer interface and material-specific constraints. Compared with silica, these polymers typically exhibit higher optical absorption and autofluorescence, narrower transparency windows, and lower tolerance to high optical intensities, which together limit low-signal modalities such as Raman spectroscopy, low-background fluorescence measurements, and quantum optical applications, while reducing long-term photostability and power-handling capability.

Chemical etching offers scalability and low-cost accessibility, with the potential to form complex lens geometries without specialized equipment. However, controlling microscale reaction dynamics to produce aspheric convex lenses spanning hyperbolic, parabolic, and prolate elliptical profiles has remained an unachieved milestone, even after decades of efforts, including doping guided etching strategies^41,42^. Since the first demonstration of lensed fibers formed by melting in 1973^43^, no fabrication method has overcome these combined limitations of geometric control, predictability, and scalability.

Here, we demonstrate the fabrication of convex ML structures on commercial optical fibers using a wet-chemical etching approach supported by a multidisciplinary modeling framework. The core strategy uses guided optical energy to induce localized heating within the surrounding etchant. Under conduction-dominated microscale conditions, this generates stable temperature gradients near the fiber surface that, through the exponential temperature dependence of chemical etching kinetics, produce spatially varying etching rates and drive deterministic geometry transformation during fabrication.

To quantitatively understand and predict this process, we developed a sequentially coupled multiphysics framework integrating optical wave propagation, laser-induced heat transfer, temperature-dependent wet-etch kinetics, and moving-boundary surface evolution. The model captures the dynamic interplay between these processes throughout etching and identifies tapered fibers as favorable initial geometries within the explored parameter space. Specifically, the combined effects of tapered geometry and tailored optical emission establish an axial temperature gradient that promotes coordinated axial recession and radial rounding, ultimately enabling deterministic formation of convex ML geometries.

Within the investigated conditions, this approach enables sub-micron control over ML curvature and allows the realization of hyperbolic, parabolic, and prolate elliptical profiles in a predictable manner. The resulting geometries provide systematic tuning of the effective working distance across different fiber configurations. Because the process relies on standard fiber materials and does not require modification of intrinsic material properties, it is readily compatible with existing fiber-based systems. In addition, the method is low-cost and scalable, requiring only conventional laboratory equipment compared to serial, point-by-point nanofabrication techniques such as FIB milling or electron-beam lithography.

To illustrate the practical impact of the fabricated MLF in demanding application scenarios, we demonstrate two representative capabilities enabled by the tapered MLF geometry. First, the enhanced optical confinement achieved by the hyperbolic ML allows optical trapping at significantly reduced power compared to conventional fiber tips. Second, using a two-step tapered MLF geometry that is challenging to realize with conventional fabrication approaches, we demonstrate non-invasive in vivo detection and manipulation of individual neuronal dendritic branches at depths exceeding 1.5 mm in the brain with a high signal-to-noise ratio. These demonstrations highlight how the integration of optics, heat-transfer, chemical kinetics, and geometry evolution enables fiber-based functionalities that are difficult to achieve using existing fabrication methods.

Overall, this work establishes a versatile platform for shaping fiber-based optical interfaces through controlled chemical etching, providing a practical route to precision light delivery and collection for applications spanning photonics, biomedicine, and sensing.

## Results

### Laser-controlled wet-chemical etching (LCWCE) for controlled formation of MLF

Precise control over etching rate profiles at the micrometer scale is critical for shaping the fiber tip into the desired structure via chemical etching. The etching process with hydrofluoric acid (HF) occurs through the following endothermic reaction^44^:

1 $${Si}{O}_{2}\left(s\right)+6{HF}\left({aq}\right)\longrightarrow {H}_{2}{Si}{F}_{6}\left({aq}\right)+2{H}_{2}O\,\left(l\right)\,\triangle {H}_{{rxn}}^{\circ }=127{kJ}$$

The etching rate follows an Arrhenius-type expression^45,46^:

2 $$r=A[{HF}]{e}^{-{E}_{a}/{RT}}$$

where *A* is the pre-exponential factor, [HF] the molar concentration of HF${E}_{a},$ the activation energy of the reaction, *R* the gas constant, and *T* the absolute temperature in Kelvin. Based on the equation, controlling the etching rate profile can be achieved by either: (1) adjusting the local concentration of the etchant, as the etching rate scales directly with the etchant concentration, or (2) precisely modulating the local temperature. Among these, temperature control is particularly practical, leveraging the ability of silica fibers to guide and emit light that is absorbed by the surrounding etchant near the fiber tip, producing localized heating and thereby enhancing the local etching rate through Arrhenius-type temperature dependence.

For a flat-cleaved fiber, optical heating absorbed by the etchant produces a predominantly radial temperature gradient with a maximum at the fiber center, which accelerates etching within the core region and leads to pit formation rather than convex shaping. In contrast, formation of a smooth ML requires an axially localized etching-rate gradient near the fiber apex together with progressive redistribution of surface recession during etching.

**Fig. 1:**
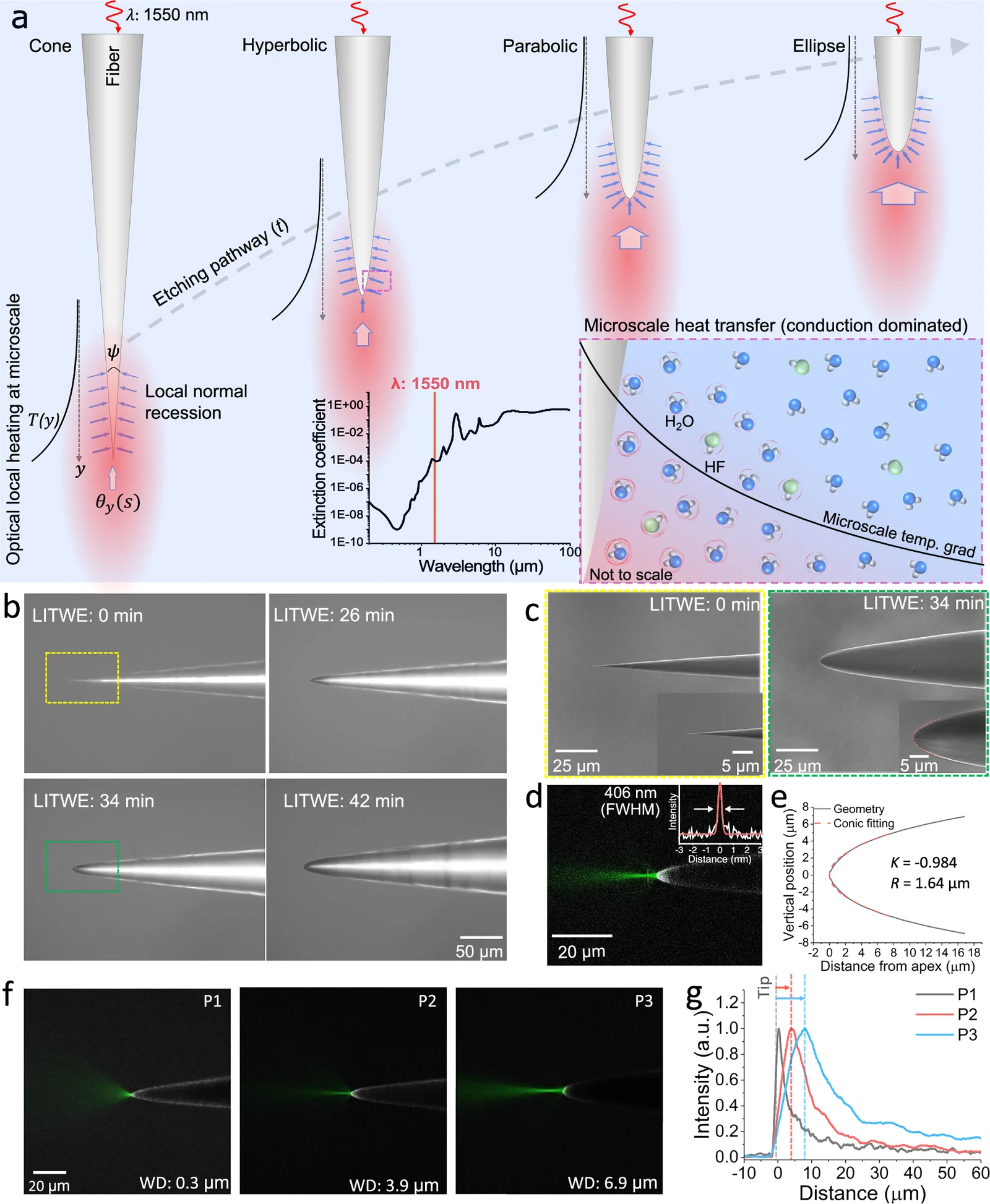
Deterministic formation of microlensed fiber (MLF) tips using laser-controlled wet-chemical etching (LCWCE). **a** Schematic illustration of LCWCE, showing the transformation of a tapered fiber into a microlensed fiber through localized thermally assisted etching. Absorption of 1550 nm light by the aqueous etchant generates localized heating and temperature gradient (*T*) near the fiber apex, producing spatially confined surface recession and progressive evolution of the tip geometry from conical to hyperbolic, parabolic, and prolate elliptical profiles via redistribution of the local recession orientation, ${\theta }_{y}(s)$, relative to the fiber axis (*y*). Insets show the absorption spectrum of the etchant and a conceptual illustration of conduction-dominated microscale heat transfer. **b**, **c** Representative optical and SEM images of fiber tips (Initial taper angle (ψ): 9°) at representative etching times (0–42 min), with insets highlighting tip morphology before and after 34 min of LCWCE. **d** Confocal fluorescence image of the MLF shown in (**c**), demonstrating optical confinement with a measured beam width of 406 nm (Gaussian fit). Beam width was measured from a single MLF at two orthogonal rotational orientations, and the averaged beam width at the focal plane with maximum intensity was reported. Fluorescent medium: 5 µM fluorescein in DI water; excitation wavelength: 488 nm. **e** Extracted surface profile of the MLF shown in (**c**) and corresponding conic fit used to determine the radius (*R*) of curvature and conic constant (*K*). **f**, **g** Demonstration of deterministic tuning of optical working distance through tip-geometry control. **f** Representative confocal fluorescence images of MLFs with different tip geometries. **g** Corresponding axial fluorescence intensity profiles showing tunable working distances of approximately 0.3, 3.9, and 6.9 µm.

A chemically tapered fiber naturally satisfies these requirements. The taper modifies the guided optical mode and concentrates optical emission near the sharp end, generating a localized temperature field near the fiber apex. Because the heated region is confined to the micrometer scale, heat dissipation remains dominated by thermal conduction with only weak local convection (Fig. 1a inset), preserving a stable axial temperature gradient that produces a localized etching-rate profile and drives controlled geometry evolution.

From a geometrical perspective, the resulting shape evolution can be understood as a continuous surface-generation process rather than the direct formation of a target conic profile. During etching, the fiber surface recedes locally along the surface-normal direction. If the etching rate were spatially uniform, the surface would recede self-similarly and preserve its initial geometry. However, preferential material removal near the apex continuously exposes a newly generated surface with evolving local normal orientations. Consequently, the recession orientation relative to the fiber axis (y), denoted as ${\theta }_{y}$(*s*), becomes distributed over a progressively larger portion of the tip surface, where s represents the position along the evolving surface. Progressive broadening of the ${\theta }_{y}$(*s*) distribution redistributes curvature along the fiber axis, enabling continuous evolution from an initially conical profile toward diverse conic geometries, including hyperbolic, parabolic, and ultimately prolate elliptical structures, analogous to the continuous geometric variation underlying classical conic families.

The evolution of the fiber tip geometry during continuous laser illumination was experimentally characterized at discrete etching durations using high-magnification optical microscopy and scanning electron microscopy (SEM) (Fig. 1b, c). Here, 1550 nm was selected as the optical energy source because it lies within a strong absorption band of the etchant while remaining readily available from standard fiber lasers (Fig. 1a inset). These measurements reveal progressive tip rounding during etching, consistent with the proposed geometry evolution.

Optical confinement at the etched fiber tip was further verified experimentally using confocal fluorescence microscopy with fluorescein (peak emission wavelength ≈ 515 nm) in water. Despite limitations associated with indirect fluorescence-based measurements, including the broad emission spectrum of fluorescein and molecular diffusion, the recorded fluorescence profiles indicate subwavelength beam confinement with an effective beamwidth of approximately 406 nm (Fig. 1d).

To provide an initial quantitative description of one resulting geometry, the measured tip profile was fitted to a conic section. A more detailed analysis of geometry evolution across fabrication conditions is presented in the following section. The extracted parameters yield a tip radius of curvature of 1.64 µm and a conic constant of − 0.984, where the conic constant describes the degree of deviation from a spherical profile and determines the resulting lens geometry, corresponding here to a prolate elliptical shape (Fig. 1e).

This level of geometric control is further validated in Fig. 1f, g. Confocal fluorescence measurements from fibers fabricated with different tip geometries demonstrate deterministic tuning of the optical working distance from approximately 0.3 µm to 6.9 µm. All measurements were performed in water, where the low refractive-index contrast between the silica fiber and the surrounding medium imposes stringent requirements on the tip geometry for efficient focusing (Supplementary Fig. 1). Under these conditions, the optical response becomes highly sensitive to the lens geometry, making precise control over transitions between hyperbolic, parabolic, and prolate elliptical shapes essential, a level of control that cannot be achieved with melting-based methods, which inherently favor spherical rounding.

To quantitatively assess fabrication precision, we analyzed fiber tips prepared in six independent LIWCE runs under the same conditions. The apex radius of curvature was measured to be 1.49 ± 0.24 µm (*n* = 6), demonstrating a narrow geometric spread and high consistency in the primary parameter governing optical performance (Supplementary Fig. 2). Notably, this level of geometric precision is achieved without specialized micromachining equipment; only a standard laser diode and wet-etching setup are required.

This precision relies on careful selection of etchant concentration and laser power to maintain stable and controllable geometry evolution. Excessive etching rates, resulting from high etchant concentration or broadened thermal profiles, promote material removal along the fiber sidewalls and suppress controlled tip rounding. Conversely, excessively high laser power can induce stronger convective flow in the surrounding etchant, degrading structuring efficiency. Through empirical optimization across multiple conditions, 10.9 wt% HF and 150 mW laser power were identified as conditions that reliably balance etching rate and process stability for single-mode fibers. Optimal conditions for multimode fibers differ due to their distinct optical mode distributions and are discussed in the following section.

### Optical-thermal-chemical mechanism governing LCWCE-driven geometry evolution

To clarify the physical mechanism underlying LCWCE and validate the observed geometry evolution, we employed a multiphysics simulation framework that decouples the dominant physical processes while preserving their quantitative physical influence on the qualitative evolution of the geometry. A fully coupled three-dimensional, time-dependent simulation would require simultaneous resolution of sub-micron optical field variations, micron-scale localized power deposition, and transient heat diffusion over spatial and temporal scales far exceeding the optical heating region. Resolving these disparate scales within a single transient framework is computationally prohibitive for laboratory-scale resources; moreover, even a fully coupled two-dimensional transient formulation remains impractical due to the combined demands of sub-wavelength electromagnetic resolution, steep thermal gradients, and long processing times.

We therefore formulated a physics-separated multiphysics modeling framework, combining (i) full wave-optics electromagnetic simulations, (ii) steady-state heat-transfer calculations, and (iii) temperature-dependent wet-etch kinetics coupled with moving-boundary geometry evolution. Optical emission from experimentally measured fiber geometries was simulated using a commercial finite-element Multiphysics solver (COMSOL) to obtain steady-state temperature fields. Surface temperature profiles extracted along the fiber boundary, parameterized by the surface anchor length ${s}_{{local}}$ referenced to the apex, were converted into local normal etching-rate kernels ${V}_{n}\left({s}_{{local}}\right)$ using Arrhenius kinetics. These kernels were subsequently applied within a fully numerical moving-boundary surface-evolution model implemented in MATLAB, which advances each surface point along its instantaneous outward normal. This approach provides a computationally tractable yet physically faithful route to capture how localized optical heating drives time-dependent tip rounding and axial recession.

**Fig. 2:**
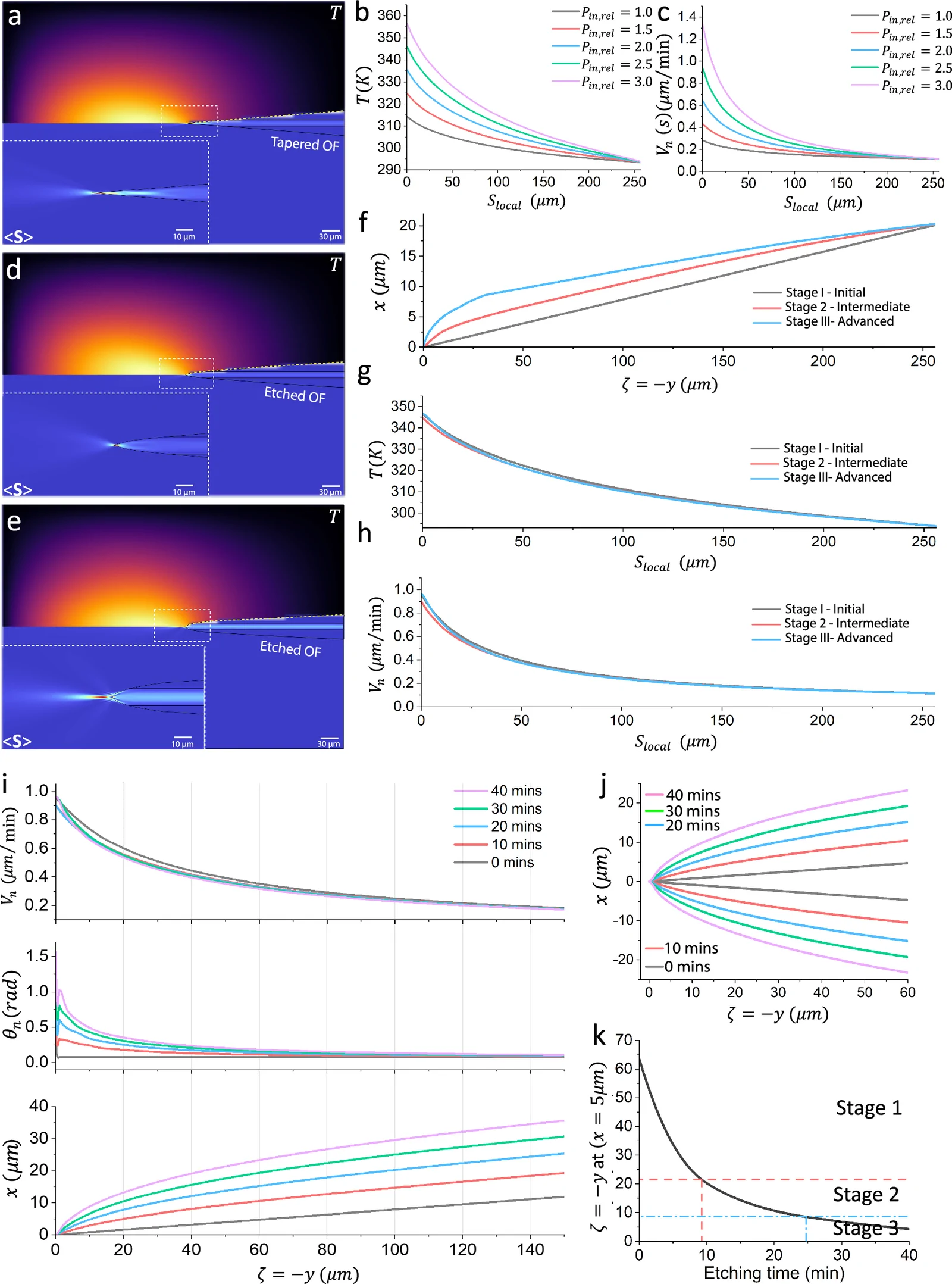
Optical–thermal–chemical mechanism governing laser-controlled wet-chemical etching (LCWCE)-driven geometry evolution. **a** Two-dimensional multiphysics simulation of the steady-state electromagnetic time-averaged Poynting vector, <**S** > , and the resulting temperature distribution generated in water by 1550 nm light emitted from the initial tapered fiber geometry (taper angle$:\,9^{\circ }$). **b** Surface temperature profiles, *T*(*s*_*local*_), extracted along the fiber surface for different relative input powers (*P*_*in,rel*_), showing localized apex heating and axial temperature decay. **c** Corresponding local normal etching-rate profiles, *V*_*n*_(*s*_*local*_), calculated from (**b**) using Arrhenius kinetics. **d**, **e** Steady-state electromagnetic and temperature distributions for experimentally observed intermediate and advanced etching geometries. **f** Extracted surface profiles corresponding to panels (**a**), (**d**), and (**e**). **g**, **h** Temperature profiles, *T*(*s*_*local*_), and corre*s*ponding etching-rate kernels, *V*_*n*_ (*s*_*local*_), extracted from the evolved geometries, demonstrating preservation of localized heating and etching during geometry evolution. Simulations in panels (**a**), (**d**), and (**e**) were performed at *P*_*in,rel*_ = 2.5. **i** Axial profiles of the applied etching-rate kernel, *V*_*n*_(*ζ)*, surface-normal orientation, *θ*_*n*_(*ζ*), and radial displacement, |*x* | (*ζ*), during geometry evolution (0–40 min). **j** Reconstructed fiber profiles showing progressive apex recession and tip rounding. **k** Apex recession evaluated at a fixed lateral position (*x* = 5 µm), defining the transition boundary used to switch between etching-rate kernels during staged geometry evolution.

Steady-state multiphysics simulations were first performed for the initial tapered fiber geometry (Fig. 2a–c). Because thermophysical properties of the etchant solution were not available experimentally, water, the dominant component of the HF solution, was adopted as the thermal medium. Thermophysical properties (density, heat capacity, thermal conductivity, and viscosity) were obtained from the embedded COMSOL material database, while the refractive index was assigned based on the literature values (Supplementary Table 1). The solid-liquid interface was treated as thermally insulating, and heat loss from the truncated liquid domain was incorporated via an effective microscale heat-flux boundary condition. At the heating volumes relevant here (equivalent heating diameter ≈ 3.6 µm; Supplementary Fig. 3), thermal dissipation is expected to occur primarily through conduction rather than bulk convection^47^, enabling stable temperature gradients that govern local etching behavior. Accordingly, an effective microscale heat-transfer description was adopted to capture the resulting thermal field. Details of the electromagnetic model, heat-transfer formulation, estimation of the effective heat-transfer coefficient, and validation of thermal robustness are provided in the Supplementary Information.

Temperature profiles were extracted along the fiber surface using the anchor coordinate ${s}_{{local}}$ (Fig. 2b). Simulations were performed in two dimensions, with optical power specified per unit length, and regions near the outer thermal boundary, where temperatures relaxed toward ambient, were excluded from analysis to avoid boundary artifacts. Local etching rates were then computed from the surface temperature using an Arrhenius relation,

3 $$r={r}_{293.15K}\bullet {e}^{\frac{{-E}_{a}}{R}\left(\frac{1}{T}-\frac{1}{293.15K}\right)}$$

where ${E}_{a}$ = 34,243 J/mol for amorphous silica^46^, *R* is 8.314 J/(mol·K), and ${r}_{293.15K}$ = 0.11 µm/min (10.9 wt% HF, experimentally determined). Figure 2c shows the resulting etching-rate ${V}_{n}\left({s}_{{local}}\right)$ profiles of the tapered fiber for different relative input powers. Through the exponential temperature dependence of Arrhenius etching kinetics, the temperature distributions are transformed into further amplified localized etching-rate gradients that drive geometry evolution.

Because the optical emission profile evolves as the geometry changes, steady-state simulations were additionally performed for experimentally observed intermediate and advanced geometries (*x*) corresponding to 26 and 34 min of etching (Fig. 2d–h). The extracted temperature and etching-rate distributions showed only modest variation across stages, indicating that the governing thermal conditions remain largely preserved throughout geometry evolution. These observations support representing the continuous process using a staged sequence of etching kernels.

Using the extracted kernels, we simulated time-dependent geometry evolution through a moving-boundary formulation:

4 $$\triangle r\,\left(x,t\right)=\,-{V}_{n}\left(s\right){{{\bf{n}}}}\left(s,t\right)\triangle t$$

where $n\left(s,t\right)$ is the outward unit normal of the evolving surface, and △t is the time increment. To maintain numerical stability without impractically small-time steps, both the evolving geometry and applied etching-rate kernels were anchored at the instantaneous apex position. This apex-referenced formulation suppresses artificial coordinate drift and ensures that the resulting evolution reflects changes in local curvature and surface orientation rather than translational motion; full numerical details are provided in the Supplementary Information.

The simulated evolution is summarized in Fig. 2i–k. Figure 2i shows the applied normal etching-rate kernels ${V}_{n}\left(\zeta \right)$, corresponding to surface-normal orientation ${\theta }_{n}(\zeta )$, and radial displacement |x | (ζ) over time. Although the kernel remains fixed within each stage, continuous changes in ${\theta }_{n}(\zeta )$ redistribute the local etching response and drive progressive tip rounding. Importantly, the increase in |*x* | (ζ) does not represent radial thickening; rather, it reflects curvature redistribution associated with apex recession and geometry evolution.

Reconstructed fiber profiles (Fig. 2j) show progressive apex recession accompanied by increasing tip rounding, in close agreement with experimentally observed morphologies (Fig. 1). Figure 2k further quantifies apex recession at a fixed lateral reference position (*x* = $5\,\mu m$), which defines the switching boundary between successive etching-rate kernels. The resulting recession curve exhibits an exponential-like decay, indicating progressive slowing of apex recession during later stages.

The simulated transition times occur earlier than experimentally observed due to the steady-state approximation, which neglects transient heating of the surrounding etchant before reaching thermal equilibrium. This discrepancy affects only the absolute time scale and does not alter the predicted evolution sequence or final geometry.

Although three etching stages were employed here, the extracted etching-rate kernels differed only modestly across stages. Simulations using a single kernel derived from the initial geometry produced nearly identical rounding behavior, indicating that the governing etching conditions remain largely preserved throughout geometry evolution.

To further assess the generality of this mechanism, we compared surface temperature distributions across fibers with different taper angles (TAs: 9°−21°). The resulting temperature profiles remained highly localized near the apex and exhibited similar spatial decay characteristics across geometries (Supplementary Fig. 4), suggesting that LCWCE operates through a common optical-thermal mechanism independent of TA. However, although the thermal mechanism remains largely preserved, its interaction with the initial taper geometry can produce distinct geometry-evolution pathways. This dependence is examined in the following section.

### Taper-angle-dependent evolution of rounded fiber tip geometries and access to distinct conic profiles

Having established the governing optical-thermal-chemical framework, we next investigated how the initial taper geometry influences the resulting tip evolution and accessible lens geometries.

**Fig. 3:**
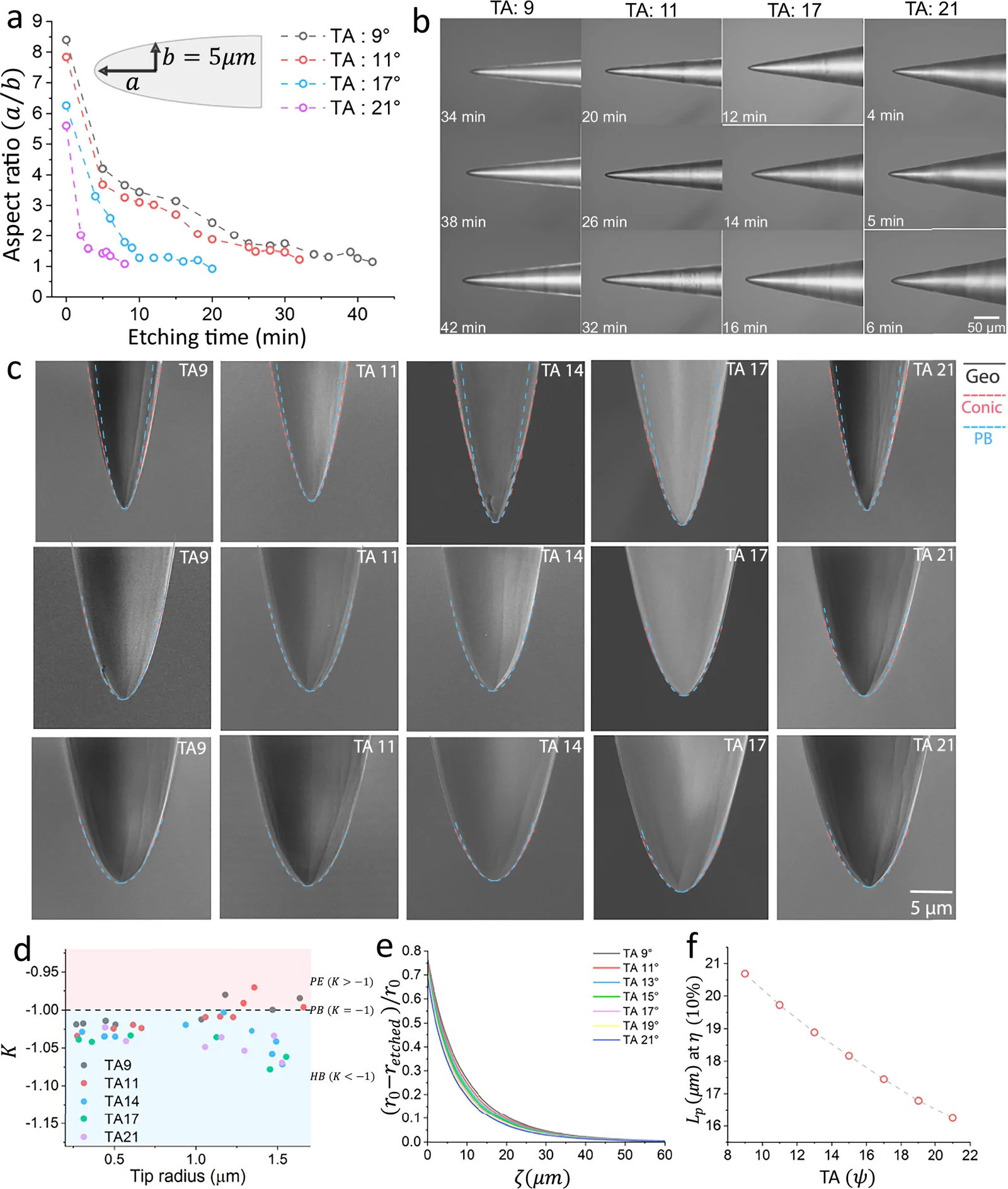
Taper-angle-dependent evolution of rounded fiber tip geometries and access to distinct conic profiles. **a** Evolution of the lensed fiber aspect ratio (*a/b*) with etching time for selected taper angles (TAs = 9°, 11°, 17°, and 21°), showing progressive tip rounding. Here, *b* = 5 µm corresponds to the mode-field radius of a standard single-mode fiber and is used for normalization. **b** Representative optical microscope images corresponding to the stages shown in (**a**). **c** Representative SEM images of fiber tips fabricated with different full TAs (9°, 11°, 14°, 17°, and 21°) at increasing tip radii; TA = 14° was additionally included to resolve the geometry transition region. Dashed curves indicate fitted conic profiles (red) and reference parabolic boundaries (blue). **d** Experimentally extracted conic constant *K* plotted as a function of tip radius for different TAs. Shaded regions denote hyperbolic (HB), parabolic (PB), and prolate elliptical (PE) regimes. **e** Normalized rounding magnitude, (*r*_*0*_ *− r*_*etched*_)/*r*_*0*_, plotted as a function of the apex-anchored axial coordinate *ζ* for different TAs. **f** Propagation length, *L*_*p*_, defined as the axial distance over which the normalized rounding magnitude exceeds 10% of its maximum value; larger *L*_*p*_ indicates broader axial redistribution and promotes transitions toward parabolic and prolate elliptical geometries.

To evaluate the fabrication dynamics, tip evolution was first quantified from optical microscope images by tracking the aspect ratio (*a/b*) of the rounded fiber tip as a function of etching time (Fig. 3a, b). Optical microscopy was used because this analysis captures overall geometry evolution rather than local apex features. Here, *b* = 5 µm corresponds to the mode-field radius of a standard single-mode fiber ( ≈ 10 µm at 1550 nm) and is used as a normalization length scale for geometric comparison. Because etching time is the directly controlled process parameter in LCWCE, this representation captures how rapidly different taper geometries evolve toward comparable rounded states.

Smaller TAs required longer etching durations to achieve equivalent levels of rounding, whereas larger TAs reached similar aspect ratios more rapidly. Representative optical images corresponding to these stages are shown in Fig. 3b, demonstrating controlled and continuous evolution of tip curvature during etching. Notably, the experimentally observed slowdown in apex recession during later stages exhibited an exponential-like decay, qualitatively consistent with the simulated evolution trend shown in Fig. 2k. This trend can be understood geometrically as a consequence of progressive tip rounding: at early stages, the extremely thin taper geometry enables small radial surface recession to produce substantial axial apex retreat because removal of a small amount of material results in large shortening of the tip. As the tip becomes increasingly rounded and thicker, radial recession becomes progressively less effective at shortening the tip, and the contribution to apex recession shifts progressively toward axial surface recession, producing the observed exponential-like slowdown.

Although aspect ratio effectively tracks fabrication progression, it does not uniquely describe the final optical geometry. We therefore characterized the resulting tip shapes using the conic constant *K* extracted from conic fitting of experimentally measured profiles (Fig. 3c, d). Higher-resolution SEM images were used for this analysis because the determination of *K* is sensitive to the local surface profile near the apex.

The conic constant provides a geometry-based descriptor independent of fabrication duration and enables classification of lens profiles as hyperbolic (*K* < − 1), parabolic (*K* = − 1), or prolate elliptical ( − 1 < *K* < 0)^48,49^. Accordingly, *K* is presented as a function of measured tip radius rather than etching time to compare final geometries independent of fabrication kinetics.

Experimental measurements reveal that the accessible conic geometry depends strongly on the initial TA. For smaller TAs ( ≤ 11°), *K* increases systematically with increasing tip radius, enabling continuous transitions from hyperbolic through parabolic and ultimately to prolate elliptical geometries. In contrast, fibers with larger TA ( ≥ 14°) remain within the hyperbolic regime even as the tip radius increases. Although variations in K within the hyperbolic region may appear moderate, crossing *K* = − 1 represents a qualitative transition in curvature distribution and therefore a distinct optical interface.

To interpret this taper-angle dependence, we used the geometry-evolution framework established in Fig. 2. Although the time-dependent multiphysics model reproduces progressive tip rounding, direct quantitative extraction of the conic constant *K* from simulated geometries is not sufficiently robust for reliable conic classification. This limitation arises from the modeling approximations required for computational tractability, including steady-state thermal assumptions and apex-anchored surface evolution, which introduce small geometric uncertainties near the apex where conic fitting is particularly sensitive, especially close to the parabolic transition.

To avoid over-interpreting fitted conic parameters, we instead introduce a geometry-based metric that quantifies how etching redistributes curvature along the fiber axis without imposing conic classification on the simulated profiles. Since parabolic and prolate elliptical geometries require curvature to extend over a broader axial region, whereas hyperbolic geometries remain dominated by near-apex curvature, the axial extent of rounding provides a more robust descriptor of geometry evolution^48^.

Specifically, we define the normalized rounding magnitude, (${r}_{0}-{r}_{{etched}}/{r}_{0}$), as a function of the apex-anchored axial coordinate *ζ* (Fig. 3e). This metric isolates the axial redistribution of curvature induced by localized etching and reveals that smaller TAs generate rounding over substantially longer axial distances than steeper tapers.

To quantify this behavior, we define the propagation length, ${{L}}_{p}$, as the axial distance over which the normalized rounding magnitude exceeds 10% of its maximum value (Fig. 3f). Smaller TAs exhibit longer propagation lengths, indicating that curvature redistribution extends farther from the apex and influences a larger portion of the tip profile. This extended redistribution enables curvature to spread over sufficient axial distance to support transitions toward parabolic and prolate elliptical geometries.

### Versatility of LCWCE across various fiber types, sizes, and structures with optimized sensing performance

Multimode fibers offer inherently higher light-coupling efficiency than single-mode fibers due to their larger numerical aperture and greater number of supported modes, making them advantageous for short-range optical communications, sensing, and biomedical applications^50^ In many of these applications, introducing optical functionality while preserving the original core geometry is beneficial because it minimizes transmission loss associated with abrupt mode transformations.

Because LCWCE forms the ML through localized surface recession confined to the optical emission region, tip functionality can be introduced without altering the original core geometry. This extends LCWCE beyond single-mode fibers and demonstrates that the fabrication mechanism is broadly compatible with different fiber geometries and configurations.

**Fig. 4:**
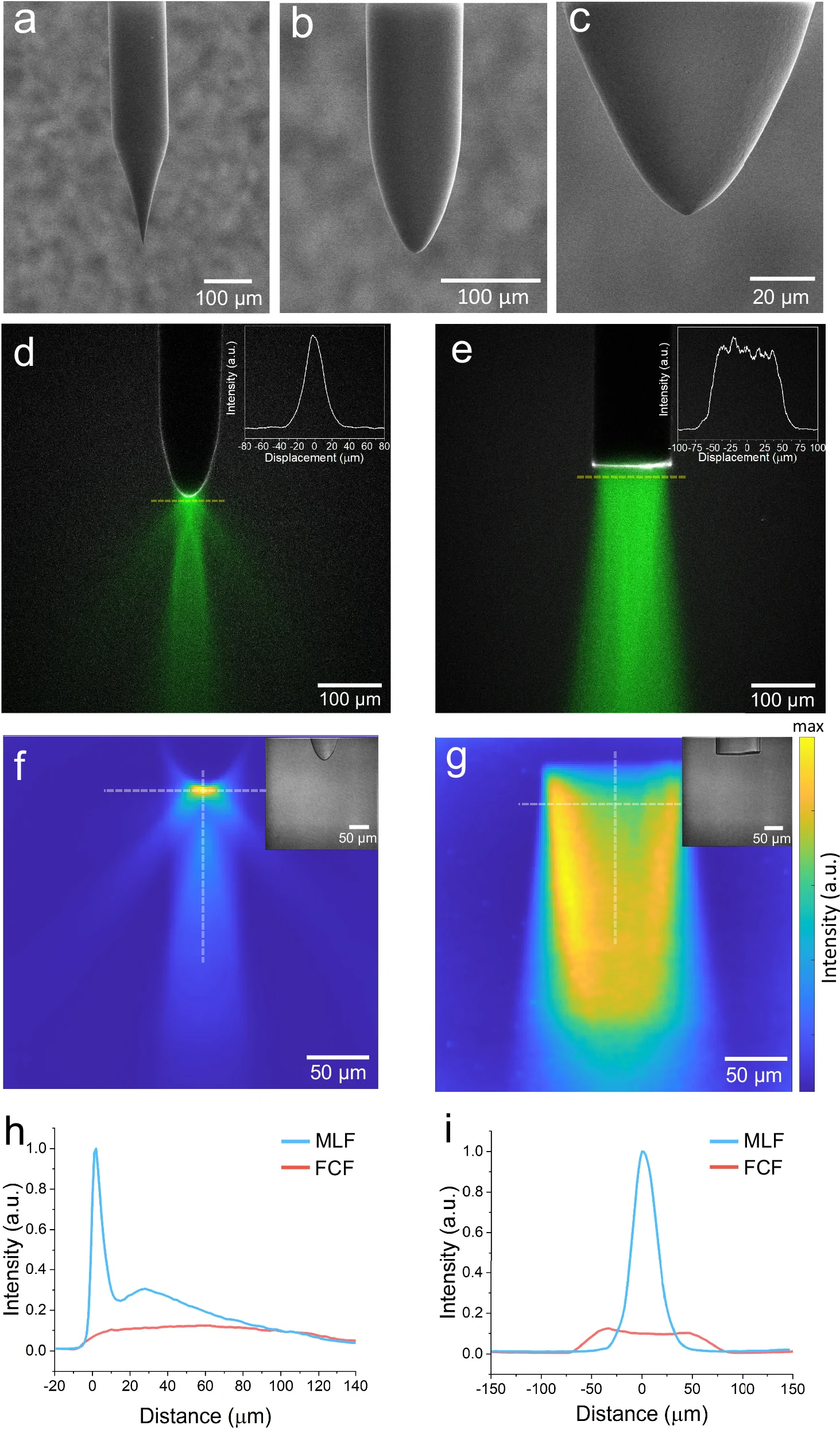
Multimode microlensed fiber (MLF) fabricated by laser-controlled wet-chemical etching (LCWCE) and comparing its radiation profile with flat-cleaved fiber (FCF). **a**–**c** Representative SEM images of the fiber tip structure before (**a**) and after (**b**, **c**) the LCWCE. The initial taper angle is 31°. Laser power and etching time: 500 mW for 10 min in 10.9 wt% HF. **d**,** e** Representative confocal fluorescence images excited by the emitted light from MLF(**d**) and FCF(**e**). The inset shows fluorescence intensity along the transverse and axial lines. Fluorescent dye: 50 µM Fluorescein in water; excitation wavelength: 488 nm. **f**, **g** Representative collection profiles of the MLF (**f**) and FCF (**g**) generated by capturing the fluorescence signal through the fiber, with fluorescence excitation achieved via two-photon microscopy ($\lambda :\,$976  nm). Fluorescent dye: 50 µM Fluorescein in water. **h**, **i** Fluorescence intensity along the transverse and axial lines in (**f**, **g**).

To demonstrate this versatility, we fabricated MLFs at the end of a 105 µm-core multimode fiber using an entirely chemical process (Fig. 4a–c). Confocal fluorescence imaging revealed a focused emission profile with a beamwidth of 24.4 µm $({\lambda }_{{ex}}=488{nm})$, substantially smaller than that of a flat-cleaved fiber (102 µm; Fig. 4d, e). The resulting geometry reduced the collection volume by 4.4-fold radially and more than 16-fold axially, producing an approximately eight-fold increase in collection efficiency near the fiber tip (Fig. 4h, i). These results demonstrate that deterministic tip shaping by LCWCE translates directly into improved optical functionality. Additional examples fabricated from smaller TAs are shown in Supplementary Fig. 5.

For applications requiring minimal invasiveness and enhanced spatial resolution, fiber diameter and taper geometry can be reduced prior to lens formation while maintaining efficient optical coupling^3,51^. To demonstrate the applicability of LCWCE to thermally pre-tapered fibers, multimode fibers were thermally pulled and subsequently shaped using LCWCE, enabling independent control of tip diameter, TA, and final lens geometry.

**Fig. 5:**
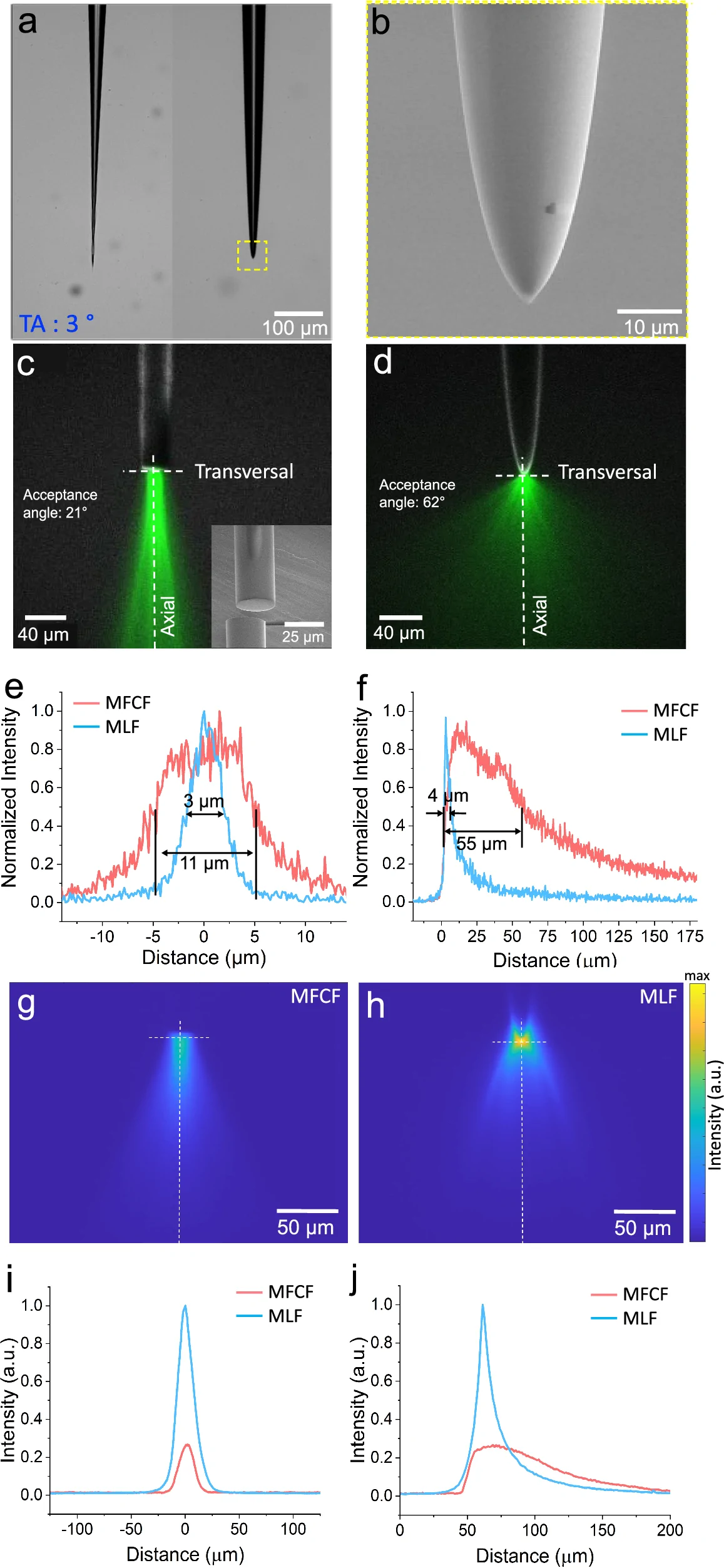
Miniaturized multimode microlensed fiber (MLF) fabricated using laser-controlled wet-chemical etching (LCWCE) and its optical characterization compared to a conventional micro flat-cleaved fiber (MFCF). **a** Representative optical microscope image of the tapered multimode fiber before and after LCWCE. Laser power and etching time: 300 mW for 10 mins in 10.9 wt% HF. **b** Representative high magnification SEM image of the MLF fiber tip. **c**, **d** Representative confocal fluorescence images excited by the emitted light from the fused ion beam-cut MFCF (**c**) and MLF(**d**). **e**, **f** Comparison of fluorescence intensity along the transverse and axial lines in (**c**) and (**d**). Fluorescent dye: 50 µM Fluorescein in water; excitation wavelength: 488 nm. **g**, **h** Representative collection profiles of the MFCF (**g**) and MLF (**h**) generated by capturing the fluorescence signal through the fiber, with fluorescence excitation achieved via two-photon microscopy ($\lambda :\,$976  nm). Fluorescent dye: 50 µM Fluorescein in water (**i**, **j**) Comparison of collected fluorescence intensity along the transverse and axial lines in (**g**, **h**).

Figure 5a, b shows a tapered multimode fiber with a 3° TA before and after LCWCE, illustrating the formation of a precisely shaped ML. Confocal fluorescence imaging reveals that the resulting emission converges to a focal spot positioned a few micrometers from the tip, achieving both transverse and axial confinement and substantially reducing the illumination volume near the fiber tip relative to a micro-tapered flat-cleaved fiber (MFCF; Fig. 5c–f). The illumination volume of the MLF is approximately 18.9 µm³, about 184 × smaller than that of a flat tip, while the collection volume is reduced to 2722 µm³, corresponding to a 5.5-fold improvement in spatial resolution and a 3.8-fold increase in collection efficiency (Fig. 5g–j), in close agreement with simulations (Supplementary Fig. 6).

Analysis indicates that the illumination profile predominantly limits spatial resolution. Enhanced focusing arises from preferential coupling of low-order modes enabled by the small TA and controlled lens geometry, whereas during collection, isotropic fluorescence excites higher-order modes, increasing collection volume and reducing resolution. Divergent emission observed when the laser is coupled near the fiber’s maximum acceptance angle further supports this interpretation (Supplementary Fig. 7).

Additional comparison of MLF and MFCF under more application-relevant conditions using fluorescent microbeads and ex vivo imaging of neuronal axon collaterals is provided in the Supplementary Information (Supplementary Figs. 8, 9), evaluating the integrated effects of the emission and collection profiles and further validating the improvements in sensitivity and spatial resolution achieved by the MLF.

Unlike single-mode fibers, multimode emission arises from a superposition of multiple propagation modes and therefore cannot be uniquely described by a single conic parameter. Accordingly, multimode MLF performance was evaluated directly through experimentally measured illumination and collection profiles rather than conic classification. This performance-based evaluation provides a more physically relevant characterization of multimode ML and avoids over-interpretation of geometric descriptors that are not well defined in the multimode regime.

### Deterministic MLF enables access to minimal power optical trapping

Fiber-based optical tweezers provide a powerful platform for manipulating microscopic objects, with applications ranging from biological systems^52,53^ to nanotechnology^54^. Compared with microscope-based tweezers, fiber-based implementations offer superior flexibility, portability, and compatibility with confined or complex environments, including microfluidic channels and in vivo settings, where bulky optical assemblies are impractical^21^.

A wide variety of fiber geometries, including tapered and lensed designs^28,55,56^, have been explored to enhance trapping performance. Among these, lens geometries that concentrate optical power into a steep axial intensity gradient near the tip are particularly effective, as trapping efficiency in aqueous environments is governed primarily by the local field gradient rather than absolute intensity. In this context, hyperbolic and near-parabolic profiles are especially attractive because they naturally generate strong, tightly confined gradients with short working distances, which are ideal for stable trapping at low optical power. In this short-working-distance regime, micron-scale particles may enter a contact or quasi-contact state after being localized near the tip, which is a widely used operating mode for fiber-based optical tweezers.

In practice, however, most reported fiber-based optical tweezers rely on assumed lens geometries derived from idealized simulations, while the actual fabricated tip shape and focal field distribution are rarely verified experimentally. Limited control over tip geometry during fabrication makes it difficult to reproducibly realize or optimize hyperbolic profiles and thus obscures the direct relationship between geometry and trapping performance.

The LCWCE process overcomes this limitation by enabling reproducible realization of tailored ML geometries. This level of geometric control is particularly important for trapping applications involving sensitive samples, where minimizing optical power is essential to reduce photothermal and photochemical perturbations. A strongly confined near-tip field gradient suppresses Brownian motion, enhances particle localization, and reduces the incident power required for stable trapping, requirements that are uniquely addressed by LCWCE-fabricated hyperbolic MLF (HMLF) fibers.

**Fig. 6:**
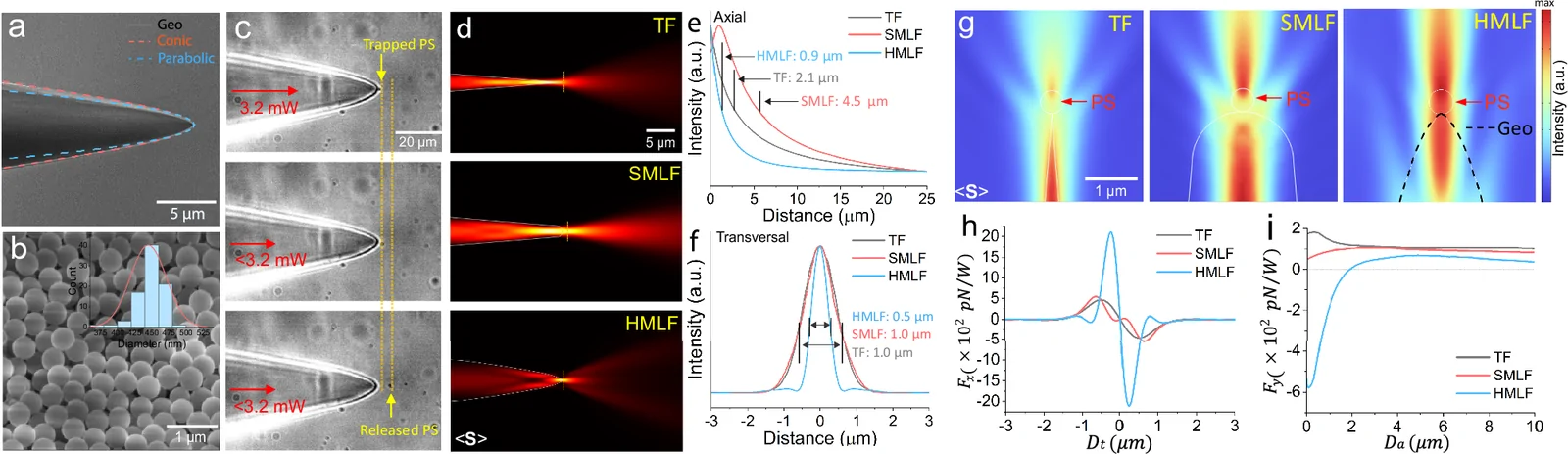
Minimal power optical trapping enabled by precision hyperbolic micro-lensed fibers (HMLFs). **a** Representative SEM image of a laser-controlled wet-chemical etching (LCWCE)-fabricated HMLF, with fitted hyperbolic (conic constant (*K)* = − 1.012) and parabolic profiles overlaid. **b** Representative SEM image of sub-wavelength polystyrene (PS) nanoparticle (diameter 0.45 µm) trapped at the HMLF tip. Inset: size distribution of PS nanoparticles. **c** Representative stable trapping at 3.2 mW (λ = 980 nm) and controlled release upon reducing the incident power. **d** Simulated optical intensity distributions in water for fiber-based optical tweezers using a tapered fiber (TF), spherical micro-lensed fiber (SMLF), and HMLF. **e**, **f** Transverse (**e**) and axial (**f**) intensity profiles extracted from d; the transverse profile is taken at the focal plane along the dashed line. **g** Simulated Poynting-vector flow, <**S** > , around a PS nanoparticle at the fiber tip, highlighting enhanced field confinement for the HMLF; the dashed outline corresponds to the experimentally measured geometry in (**a**). **h**, **i** Calculated transverse (**h**, *F*_*x*_) and axial (**i**, *F*_*y*_) optical forces acting on the PS nanoparticles as a function of particle–tip separation (*D*_*t*_: Transverse displacement; *D*_*a*_: Axial displacement). The transverse force *F*_*x*_ is evaluated at zero axial offset.

To demonstrate this capability, we fabricated HMLFs (*K* = − 1.012) optimized for optical tweezer operation (Fig. 6a) and evaluated their trapping performance using the fiber-based tweezer system shown in Supplementary Fig. 10. Trapping efficiency was tested with near-half-wavelength polystyrene nanoparticles (PS, diameter: 445 ± 26 nm) at 980 nm (Fig. 6b inset). These particles closely mimic biologically relevant vesicles in both size and density. Although initial capture required higher power due to Brownian motion, stable trapping was maintained at an incident power of only 3.2 ± 1.2 mW (*n* = 4) (Fig. 6c), more than 5× lower than typical fiber-based optical tweezers reported previously^30,56^.

This result directly demonstrates that LCWCE enables not only efficient fabrication of MLFs, but also deterministic realization of hyperbolic geometries optimized for low-power optical trapping. The ability to predictably engineer and verify lens geometry establishes LCWCE as a powerful platform for precision fiber-based optical manipulation in biologically sensitive and space-constrained environments.

To benchmark trapping performance, we compared the emission characteristics of HMLFs fabricated by LCWCE with those of well-established fiber geometries, including tapered fibers^57,58^ and spherically rounded MLF (SMLFs)^31^, produced using established chemical and laser-assisted techniques. The LCWCE process enables precise control over lens curvature, allowing access to well-defined hyperbolic geometries that are difficult to achieve consistently with conventional approaches.

Axial and transverse profiles (Fig. 6d–f) show enhanced optical confinement in both directions relative to the comparison geometries. Corresponding optical force calculations for 450 nm PS nanoparticles indicate increased axial and transverse trapping forces (*F*_*y*_ and *F*_*x*_, respectively) consistent with stronger field gradients near the tip (Fig. 6g–i). This enhanced axial attraction facilitates sub-wavelength localization at reduced optical power and promotes stable particle positioning near the fiber apex. In contrast, conventional MLFs fabricated by melting-based rounding were observed to trap only larger particles (radius > 0.94 µm; Supplementary Fig. 11), reflecting their more limited field confinement at comparable powers.

To further assess trapping behavior under reduced Brownian motion, we also examined trapping of borosilicate microbeads (diameter, 7.34  ±  0.87 µm), whose refractive index ( ~ 1.5) is comparable to that of many soft-matter and biologically relevant materials^3^ (Supplementary Fig. 12). Stable trapping was achieved at an average incident power of 0.625  ±  0.35 mW (*n*  =  4), with successful trapping observed at powers as low as 0.5 mW. The lower required power relative to nanoparticle trapping is consistent with the reduced influence of Brownian motion for larger particles. Trapped microbeads could be translated over distances exceeding 60 µm in both axial and transverse directions and were released immediately upon laser shutoff (Supplementary Fig. 12g, h).

When compared with representative values reported for heat-and-pull fiber-based optical tweezers^30,59^, the trapping powers required here are reduced by approximately one order of magnitude. This reduction is consistent with the enhanced optical confinement enabled by the hyperbolic tip geometry. Together, these results highlight the importance of geometric control in reducing trapping power requirements and demonstrate the potential of LCWCE-fabricated HMLFs for low-power optical manipulation. Video recordings of representative trapping experiments are provided in the Supplementary videos.

### Lens formation on ultrathin two-step tapered fibers enables high-resolution neural probing deep within live brains

Microfiber optic probes hold exceptional promise for neuroscience, biosensing, and biomedical applications. They can deliver light deep into tissue and enable single-cell-level modulation and sensing beyond 1.5 mm, depths where many optical methods fail^3,60^. Their scalability into multi-fiber arrays allows simultaneous measurements and manipulations over wide areas, making them indispensable for large-scale biosensing and medical research^61–63^. This capability could transform our understanding of complex biophysiology. For example, minimally invasive monitoring of independent neural activity within the same genetic type^64–66^ demands optical neural interfaces with enhanced spatial resolution and sensitivity approaching the single-cell scale. Recent advances in optical neural interfaces have demonstrated increasingly localized recording and modulation of neural circuits, including deep-brain interrogation with subcellular spatial resolution and stimulation of individual neuronal compartments^67,68^. Nevertheless, translating such precision into practical neural interfaces remains challenging because achieving high spatial selectivity often comes at the expense of optical throughput, scalability, and reliable operation at depth. These limitations create increasing demand for optical probes capable of maintaining micron-scale optical confinement while preserving efficient light delivery in deep tissue. Back propagating action potential along micron-diameter neural dendrites, spikes are now recognized as critical regulators of neural computation and output^69–71^, further emphasizing the need for optical interfaces that enable precise interrogation of subcellular structures deep within the brain.

**Fig. 7:**
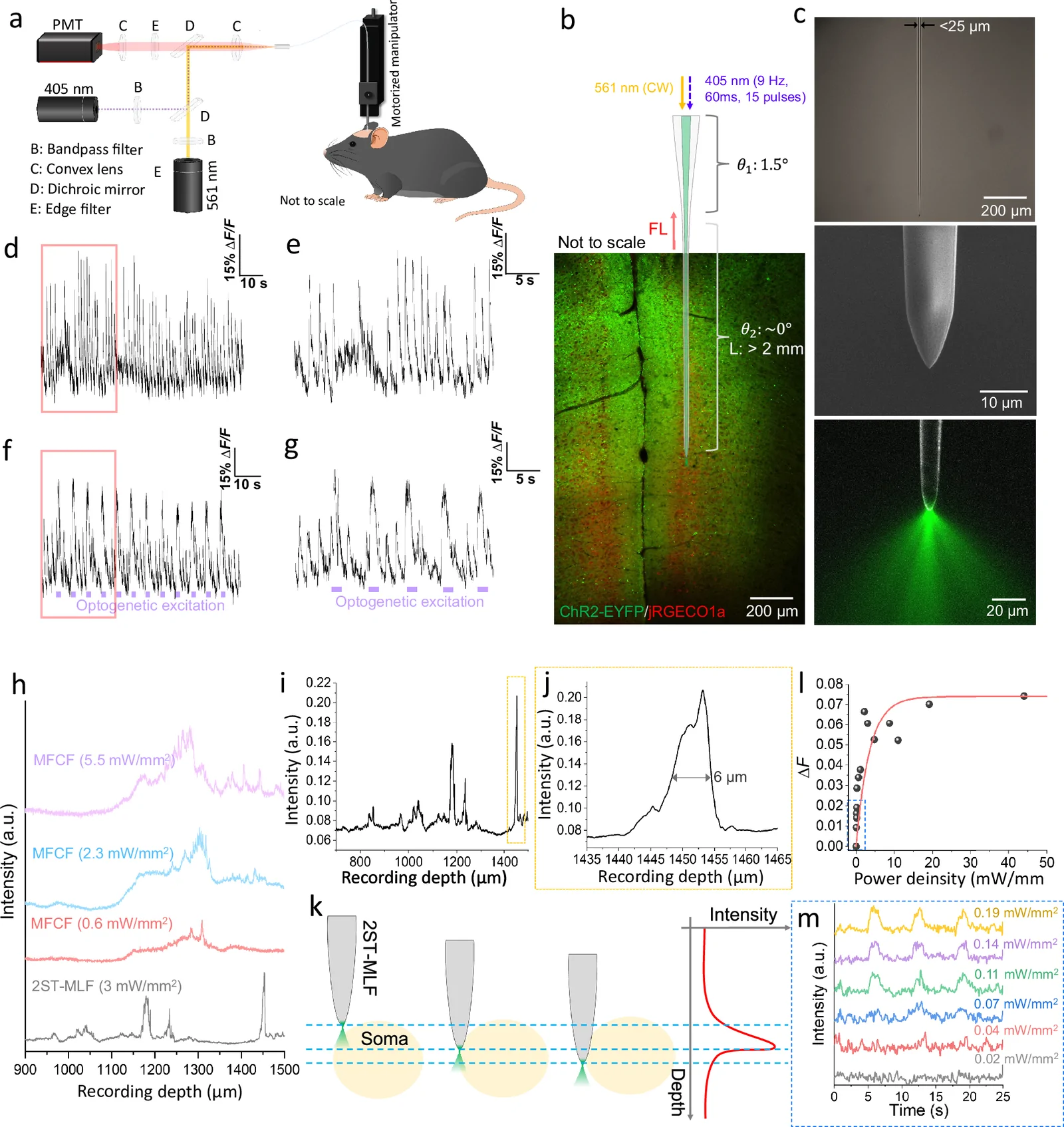
“All-optical” modulation and recording of single-cell activity using a two-step tapered microlensed fiber (2ST-MLF) in vivo. **a** Schematic of the optical setup. **b** Schematic of the two-step tapered 2ST-MLF inserted into a mouse brain. **c** Representative optical (i), SEM (ii), and confocal fluorescence (iii) images of the two-step tapered 2ST-MLF tip. Fluorescent dye: 50 µM Fluorescein in water. Excitation wavelength: 488 nm. **d** Spontaneous Ca^2+^ fluctuations in a hippocampal neuron (Depth: 1.6 mm; Ca^2+^ indicator: jRGECO1a; excitation wavelength: 561 nm; photomultiplier tube (PMT) gain: 10^6^. **e** Close-up view of the highlighted region in (**d**). **f** Ca^2+^ fluctuations in a hippocampal neuron induced by spontaneous and repeated optogenetic photoactivation. (Depth: 1.6 mm; Ca^2+^ indicator: jRGECO1a (561 nm, 3 mW/mm^2^); Optogenetics: ChR2 (405 nm, 20 mW/mm^2^, 9 Hz train of 15 pulses, 60 ms pulse duration, 5 s delay between pulse trains)) (**g**) Close-up view of the highlighted region in (**f**). **h** Representative enhanced signal-to-noise ratio (SNR) of 2ST-MLF compared to a conventional micro flat-cleaved fiber (MFCF) by excluding background noise from untargeted cells. **i** Close-up view of PMT outputs for the 2ST-MLF as a function of recording depth. **j**, **k** Asymmetric intensity variation of jRGECO1a fluorescence with respect to the approach and pass-by of the 2ST-MLF. **l** Representative fluorescence intensity changes as a function of laser power, showing saturation and confirming the single cell recording volume of the 2ST-MLF. **m** Close-up view of the blue dashed box in (**l**), demonstrating the high sensitivity of the 2ST-MLF.

Reducing fiber diameters to a few micrometers decreases invasiveness and narrows lateral emission and collection profiles to subcellular dimensions^3,60^. However, this improvement comes with trade-offs: a narrow optical window limits fluorescence collection, and poor axial confinement reduces excitation specificity and SNR. For example, the MFCF in Fig. 5 can resolve adjacent cells laterally, but its 55 µm axial confinement, larger than an average cell, yields low SNR in densely packed regions. To achieve true single-cell precision, the illumination and recording volumes must be comparable to the target cellular compartment ( ~ 5 µm diameter, ~ 75 µm³ volume^72^). Incorporating an ML at the fiber tip confines both illumination and collection, extends the effective acceptance angle beyond the intrinsic NA of the fiber, and significantly improves SNR. Achieving minimal invasiveness together with enhanced spatial resolution and sensitivity requires a probe geometry that combines an ML with an elongated shaft ( > 1.5 mm) and an ultrathin diameter. Conventional fabrication methods cannot reliably produce such small, long, and precisely shaped fiber tips. LCWCE overcomes this limitation by enabling independent control over TA and tip size, allowing the fabrication of two-step tapered MLFs (2ST-MLFs) optimized for high-resolution operation in confined environments such as deep-brain tissue and microfluidic systems. In this design, the fiber diameter is first reduced from 125 µm to 25 µm using a shallow 1.5° taper to ensure low-loss mode transformation^73^, followed by a near-zero-angle taper that yields tip diameters below 15 µm with lengths exceeding 2 mm (Fig. 7b, c). These ultrathin tapers are subsequently sculpted into MLs via LCWCE without practical constraints imposed by the reduced fiber diameter.

In vivo, 2ST-MLFs were inserted into the brains of anesthetized mice in regions where neurons were transduced with the blue light activatable opsin channelrhodopsin (ChR2-EYFP) for optogenetic stimulation and the genetically encoded Ca^2+^ sensor jRGECO1a to monitor activity. jRGECO1a was chosen to enable Ca²⁺ imaging with red-shifted excitation ( > 561 nm) to minimize ChR2 cross-activation^74,75^. Fibers reached depths > 1.5 mm with minimal trauma. Single-cell targeting in the cortex yielded over 47 Ca²⁺ transients in one minute, averaging 40% Δ*F/F* (Fig. 7d,e). Photostimulation at 405 nm (9 Hz) increased responses to 57% Δ*F/F* (Fig. 7f, g), marking the first single-cell optical recording during photostimulation with a single fiber probe, achieved without pre-photobleaching^76,77^ and despite the lower brightness and dynamic range of jRGECO1a compared to GCaMP^70^, often used in previous studies.

Axial scanning revealed a depth resolution of 6 µm when passing a soma (Fig. 7h–j). Fluorescence intensity increased as the probe approached a neuron and dropped sharply after passing, producing an asymmetric profile attributed to the 2ST-MLF’s highly focused illumination and smooth, rounded tip, which allows gentle displacement without damage. Approach-retraction experiments confirmed reproducibility, and peaks as narrow as 1.6 µm were detected at 1.6 mm depth (Supplementary Fig. 13), highlighting the ability to resolve fine neural structures deep in the brain where other techniques risk tissue injury.

In vitro tests (Supplementary Figs. 14–16) confirmed that photomultiplier tube (PMT) output changes were driven by Ca²⁺-indicator fluorescence from spontaneous and photo-evoked activity. Dendritic recordings (Supplementary Fig. 15) showed intensity changes correlating with action potentials. The MLF consistently isolated single-cell peaks while suppressing background noise, in contrast to MFCFs, which exhibited higher noise in dense regions. Reducing optical power lowered noise but also peak amplitude, whereas the MLF maintained high SNR.

Recording volume was further assessed by varying photostimulation power. If the probe illuminates multiple cells, fluorescence intensity should increase proportionally with incident power. In contrast, when illumination and collection are confined to a single cell, the signal should plateau once all ChR2 channels are activated, and membrane depolarization reaches its maximum. In our measurements, fluorescence saturated at 20 mW/mm², confirming single-cell confinement, and remained detectable at powers as low as 70 µW/mm² (Fig. 7l, m). This exceptional sensitivity, combined with the ability to detect subtle ionic fluctuations and resolve fine structural detail, enables a more comprehensive and precise analysis of neural circuits.

The 2ST-MLF minimizes invasiveness. Its footprint is 66× smaller than a 125 µm fiber and 244 × smaller than a 250 µm fiber (Supplementary Fig. 17a), suggesting that 66 2ST-MLFs would cause the same tissue impact as one 125 µm fiber, and 244 2ST-MLFs as one 250 µm fiber, allowing high-density arrays with minimal harm. Implanting four 2ST-MLFs into the striatum at 3-4 mm depth caused negligible astrocyte (GFAP) and microglia (Iba-1) activation compared to conventional fibers (Supplementary Fig. 17b), which still produced noticeable damage, especially at 250 µm (Supplementary Fig. 17c, d). Quantitative fluorescence analysis confirmed the reduced tissue response, even with multiple probes (Supplementary Fig. 17e, f).

These results establish the 2ST-MLFs as a powerful neural probe, capable of precise single-cell recordings, exceptional spatial resolution, and ultra-low invasiveness. Combined with LCWCE’s batch-fabrication capability, ensuring precise tip control regardless of taper geometry, this approach uniquely enables high-throughput production of specialized lensed fibers for neuroscience, biosensing, imaging, and minimally invasive deep-tissue light delivery, offering high spatial resolution, superior SNR, and scalability for large-area applications.

## Discussion

We introduce LCWCE as a deterministic and scalable strategy for forming aspheric MLFs with sub-micrometer control over tip geometry. Localized optical heating at the fiber apex establishes an axially confined temperature field that generates a decaying etching-rate profile along the surface. This apex-localized, anisotropic etching enables continuous reshaping of the fiber tip and systematic access to hyperbolic, parabolic, and prolate elliptical geometries through etching time alone.

To elucidate the underlying mechanism, we developed a physics-separated multiphysics framework integrating wave optics, steady-state heat transfer, and temperature-dependent etching kinetics coupled with moving-boundary geometry evolution. This reduced-order model captures the essential features of the process and reproduces the experimentally observed transition from sharp tapers to smoothly rounded lenses. The agreement confirms that LCWCE-driven lens formation arises from apex-localized optical heating rather than isotropic material removal, validating the physical mechanism without reliance on fully coupled transient simulations.

While laser-assisted wet etching itself is not novel, prior studies have largely relied on fixed or nominal starting geometries and have not explicitly analyzed the microscale thermal transport regime in liquid etchants or used it to guide geometry evolution. In contrast, our experiments and physics-based modeling demonstrate that under LCWCE conditions, localized temperature gradients persist and directly govern deterministic convex ML formation. This understanding enables a reverse, geometry-informed design strategy, in which the initial taper geometry is intentionally selected to achieve a targeted class of final lens shapes.

Within this framework, the final lens geometry is governed jointly by the etching duration and the initial TA. Shallow tapers allow etching-induced rounding to propagate over longer axial distances, enabling transitions toward parabolic and prolate elliptical profiles, whereas steeper tapers confine rounding near the apex and favor the persistence of hyperbolic geometries. Together, these parameters define a compact and experimentally accessible design space for deterministic ML engineering.

This level of geometric control enables two previously inaccessible regimes. First, LCWCE allows deterministic optimization of HMLF for minimal-power optical trapping by enhancing optical confinement and field gradients at the fiber tip. Second, LCWCE uniquely enables lens formation on ultrathin, two-step tapered fibers that support deep, minimally invasive, high-resolution neural probing beyond 1.5 mm.

At present, LCWCE operates in an open-loop configuration without real-time etching feedback. While high geometric quality and reproducibility are achieved under moderate processing conditions, aggressive operation aimed at maximizing throughput, such as excessive laser power or etchant concentration, can compromise process stability. These limitations motivate future integration of closed-loop control, in situ optical or thermal monitoring, and more advanced inverse-design modeling to further enhance precision, robustness, functional versatility, and throughput.

## Methods

### Fabrication of microprobes with a different tip structure (MLF and FC)

The creation of MLFs using LCWCE processes begins with the initial modification of the fiber tip structure from a flat surface to a taper through controlled chemical etching. A uniformly and smoothly tapered structure is essential to obtain a precision MLF in the following step. To begin, the fiber jacket is carefully removed at one end, followed by cleaning the residues through sonication in acetone, ethanol, and DI water for 5 min each to guarantee a clean surface, then it is completely dried. For the initial tapering process, we utilized a diluted solution (25.9 wt%) of hydrofluoric acid (HF, 48 wt%, Fisher Scientific) created by mixing 48% HF with deionized (DI) water in a 1:1 volume ratio. Iso-octane (99%, Fisher Scientific) was added to the etchant to prevent HF vaporization, ensuring a stable etching environment.

The desired TA was achieved by precisely controlling the etchant container’s height using a motorized vertical translation stage (MLJ250, Thorlabs) placed on vibration-isolating Sorbothane feet. The travel speed of the stage is from 0 − 0.7 μm/sec, providing a TA of 30 to 7 °. (Supplementary Fig. 18) This approach allowed fine-tuning of the taper geometry, enabling precise control over the TA and tip dimensions, which are critical for tailoring the MLF to specific applications^58,78^. After removing the fiber from the etchant, the fiber was thoroughly washed using DI water.

For MLF fabrication, chemically pre-tapered fibers were immersed in a 10.9 wt% HF solution and subjected to continuous laser illumination throughout the etching process. A 1550 nm laser, generated by an erbium-doped fiber amplifier (Center for Optics, Photonics, and Lasers, Quebec City, Canada) and pumped by a 976 nm laser diode (K976DA3RN-30W), was coupled into the fiber to induce localized heating at the tip and drive LCWCE (Fig. 1). The laser power was set to 150 mW for single-mode fibers (F-SMF-28, Newport) and 500 mW for multimode fibers (FG105UCA, Thorlabs). The laser remained on continuously for the entire etching duration, during which the fiber-tip geometry evolved under laser-induced temperature gradients. Immediately upon completion of the LCWCE process, the laser was turned off, and the fiber was removed from the etchant, thereby terminating the etching reaction. The fiber was then rinsed with deionized water to remove residual HF. No further etching occurred after laser shutdown. Prior to immersion, the laser output was stabilized in air for at least 15 min to ensure consistent optical power delivery during the shaping process.

For the MLF for its biosensing applications, we used the multimode fiber with a 50 μm core diameter (FG050UGA, Thorlabs). To prepare the tapered fiber, we first cut a 1.5 m long segment and then removed a portion of the polymer jacket from the middle. We cleaned any remaining residues using cotton soaked in acetone and ethanol. Next, the fiber underwent CO_2_ laser splicing (LZM 100, AFL) to create a TA of either 1.5° or 3°, a taper waist diameter ranging from 20 μm to 35 μm, and a taper waist length of 20 mm. The specific tip size and target depth for recording purposes influenced the dimensions chosen during the tapering process. Finally, the taper waist was cleaved using a ruby fiber scribe. We used the FCF directly without needing LCWCE.

For the single tapered MLF for its biosensing applications, a tapered fiber with a taper waist diameter of 20 μm and TA of 3° was used. The tapered fiber end underwent a cleaning process involving acetone, ethanol, and water under gentle sonication. Subsequently, the fiber was immersed in a 48 wt% HF solution for 8 minutes, leading to the complete etching of the taper waist and the creation of a sharp tip, as depicted in Fig. 5a. Next, the fiber was transferred to a 10.9 wt% HF solution, and a 300 mW 1550 nm laser (erbium-doped fiber amplifier, COPL) was guided through the fiber tip for 10 min to create the ML at the end, followed by a rinse with DI water (Fig. 5a).

In the case of the 2ST-MLF, a tapered fiber with a taper waist diameter of < 25 μm and TA of 1.5° was utilized, following the same cleaning procedure as for the single-tapered MLF. The tapered waist of 4 mm was then immersed in a 25.9 wt% HF solution for 47 min while the fiber was drawn out at a constant speed of 0.7 μm/sec to create a sharp tip (TA: 9°). To save time, multiple fibers were prepared at the same immersion using our custom-built automated system. Afterward, the fiber was returned to the 25.9 wt% HF solution, and a 200 mW 1550 nm laser was guided through the fiber tip for 7 min, followed by a rinse with DI water.

### Trapping particles using MLF

A MPC-200 micromanipulator (Sutter Instrument) was positioned under an optical microscope equipped with a 40x objective lens (NA: 0.75) to observe the manipulation process (Supplementary Fig. 10). A charge-coupled device (CS2100M, Thorlabs) was used for real-time monitoring and image capture. A 980 nm laser (2000 CHP, 3SP) from the laser source was coupled into an optical splitter. The optical power was measured at the 10% output, which was connected to an optical power meter, while light from the 90% output was directed into the MLF immersed in a water suspension of borosilicate (Duke Standards^TM^) or polystyrene (carboxylate-modified, Sigma Aldrich) particles on a PDMS substrate. The fiber, enclosed in a glass capillary, was secured onto the 3D micromanipulator to allow precise positioning. The suspension was prepared by dispersing the particles in deionized water and treating them ultrasonically for 10 min to achieve a uniform particle distribution. The suspension was diluted until only a few particles were visible within the field of view, corresponding to an approximate particle-to-water weight ratio of 1:1000. After preparation, the suspension was applied onto a PDMS substrate.

### Emission profile of MLF, MFCF, and FCF

To analyze the emission profile of the MLF, FCF, and MFCF, we submerged the fiber ends in a fluorescent dye solution (50 μM Fluorescein in DI water). The Fluorescein in the solution was excited using a 488 nm laser (FCD488 20 mW 488 nm, JDS Uniphase) that was guided through the fiber. We then captured fluorescent images of Fluorescein using a confocal microscope (Zeiss LSM 880) equipped with a 40 x water immersion lens. (Supplementary Fig. 19a).

### Collection profile of MLF, MFCF, and FCF

To evaluate the collection profiles of the MLF, MFCF, and FCF, we conducted an experiment by submerging the fiber ends in a fluorescent dye solution containing 50 μM Fluorescein dissolved in deionized water. The excitation of Fluorescein was achieved using a two-photon microscopy system equipped with an Acousto-Optic Deflector (Karthala), and a 976 nm laser (Supplementary Fig. 19b). The scanning was performed at a resolution of 200 × 200 pixels, with a pixel dwell time of 500 microseconds. Fluorescence signals emitted from the dye were collected through the optical fibers, which were filtered using two filter sets (AT 494lp and ET525/50 M, Chroma) and focused on a PMT (H6780-20, Hamamatsu) detector using a plano-convex lens (focal length = 50 mm, LA1131-A, Thorlabs). The PMT output was amplified (10^6^). The collected PMT signals using Spike2 were subsequently processed in MATLAB to construct 2D map profiles, which represent the spatial distribution of fluorescence corresponding to the two-photon scanning data.

### Assessing spatial resolution of MLF and FCF using fluorescent microbeads

To prepare the Agar gel, we added 0.05 g of Agar (Sigma Aldrich) powder into a beaker containing 20 ml of DI water while stirring continuously. The mixture was heated on a hot plate with continuous stirring until the Agar powder completely dissolved. After complete dissolution, we removed the solution from the heat source and allowed it to cool slightly^79^. Then, we added 2 μl of 1 μm latex bead solution (Carboxylate-modified polystyrene, fluorescent yellow-green, Sigma-Aldrich) to the Agar solution, stirring thoroughly. The combined solution was poured into a 35 mm petri dish and left to solidify. Subsequently, the gel was sectioned perpendicular to the probe axis, exposing microbeads located near the cut surface. Fluorescence was excited and collected through the MLF while the probe was systematically scanned laterally and axially relative to the immobilized bead (Supplementary Fig. 8). These measurements were compared with those obtained using a tapered MFCF of similar tip diameter (Supplementary Fig. 8a, c).”

The microbead was excited using a laser as the excitation light source (FCD488 20 mW 488 nm, JDS Uniphase). The laser light passed through a laser line filter (FBH488-3, Thorlabs) and a neutral density filter set (N.D. 1-3, NEK01, Thorlabs), and then it was reflected on a dichroic beam splitter (FF495-Di03, Semrock). To direct the beam into the fiber end, we used a fiber collimator (F220FC-532, Thorlabs). The fluorescence of the microbead was excited by the emitted light from the microprobe (30 μW). The emitted fluorescence was collected through the fiber tip, propagated back, collimated by the collimator, and then passed through the dichroic beam splitter. The fluorescent light was filtered using two filter sets (AT 494lp and ET525/50 M, Chroma) and focused on a PMT detector using a plano-convex lens (focal length = 50 mm, LA1131-A, Thorlabs). To prevent external light interference, the entire optical system was enclosed in a light isolator box. The PMT output was amplified and filtered with a low-pass filter set at 25 Hz.

### Experiments with cells and animals

#### Animals

The rodent experiments were carried out on adult male C57BL/6 J mice purchased from the Jackson Laboratory (RRID:IMSR_JAX:000664). Male Gad2-IRES-Cre (Gad2^tm2(cre)Zjh^/J) were purchased from the Jackson Laboratory (RRID:IMSR_JAX:010802) and bred with female Ai32(RCL-ChR2(H134R)/EYFP) mice^80^ (RRID:IMSR_JAX:024109) to produce the Gad2-CreXRosa-CAG-ChR2 mice used to obtain brain sections. Mice were housed on a 12 h light/dark cycle with *ad libitum* access to food and water at 21 ± 1 °C and 40–45% relative humidity. All experimental protocols were approved by the Committee for Animal Protection of Universite Laval (CPAUL) in accordance with the guidelines from the Canadian Council for Animal Care.

### Viral constructs

The AAV expressing ChR2-EYFP (AAV2/1.hSyn.hChR2(H134R)-EYFP; titer 1.8 × 10^13^ GC mL^−1^) was produced by the Canadian Neurophotonics Platform Viral Vector Core Facility (RRID: SCR_016477). The AAV expressing jRGECO1a (AAV9.SynNES-.jRGECO1a.WPRE.SV40; titer 1 × 10^13^ VG mL^−1^)^74^ was a gift from Douglas Kim and the GENIE project (Addgene viral prep # 100854-AAV9).

### Assessing the sensitivity of MLF and MFCF using brain slices

Brain slices were obtained from adult male transgenic mice expressing ChR2 and EYFP under the control of the GAD65 promoter. The experimental procedures followed the guidelines of the Canadian Council on Animal Care and were approved by the Animal Care Committee at Laval University. For the experiments, the mice were anesthetized with 30% urethane (dose of 0.1 ml/10 g) before undergoing intracardiac perfusion with 4% paraformaldehyde. Brains were then extracted and preserved overnight in 4% paraformaldehyde at 4 °C. Fixed brains were cut into 100 μm slices using a vibratome (VT 1000 s, Leica) and submerged in 1 x PBS.

The neurons in the prelimbic area were observed under 470 LED light sources using a 40 × water-immersion objective and a charge-coupled device (CCD) camera (Retiga ELECTRP, Teledyne Photometrics). The MLF and MFCF were mounted and manipulated with an MPC-200 micromanipulator (Sutter Instrument). Comparative measurements between MLF and MFCF were performed on the same region of the same brain slice whenever possible to minimize sample-dependent biological variability. Spatial resolution and sensitivity were assessed by translating the probes transversally at a constant speed while recording from the same area. The fluorescence in the brain slices was excited and collected using the same optical setup used for the fluorescence microbead test.

### Single-cell optical stimulation and recording of neural activity using MLF and patch-clamp with cultured cells

#### 4.1) Neuronal primary cultures

Dissociated cortical neurons were obtained from the Neuronal cultures platform at the CERVO Brain Research Center (PCN-CERVO) following their standardized procedures^81^. Briefly, postnatal (P0-P1) Sprague-Dawley pups were anesthetized in ice and decapitated. The neocortex was then dissected and dissociated enzymatically, with 12 U/mL papain solution (Worthington Biochemical Corporation) and mechanically with a Pasteur pipette. Subsequently, neurons were washed, centrifuged, and plated in clear coverslips (12 mm) at a density of 200 K cells per coverslip. Cells were then maintained using neurobasal media (Gibco) supplemented with B27 (50:1), 50 U/ml penicillin/ 50 µg/ml streptomycin mixtures, 0.5 mM L-glutamax and 2% FBS-HI. To reduce the number of non-neuronal cells, 5 μM of cytosine arabinofuranoside (ARA-C; Sigma) was added on day in vitro 5 (DIV5), and cells were kept in culture for 4 additional days in the presence of ARA-C. Twice a week thereon, half of the growth medium was replaced with medium without ARA-C. Neurons were maintained at 37 °C in a humidified atmosphere of 95% air and 5% CO_2_.

#### 4.2) Viral transfection of Neuronal primary cultures

Neuronal primary cultures were virally transfected with channelrhodopsin 2 (AAV2/1-hSyn-hChR2(H134R)-EYFP; Molecular Tools Platform - CERVO, lot 925, titer 1.8E13) and/or JR-GECO (pAAV.Syn.NES-jRGECO1a.WPRE.SV40; Addgene, #100854-AAV9, lot V23799, titer 1.8E13) on DIV9. Neurons were maintained in 500 μL of growth neurobasal media supplemented with 0.2 μL of virus for 1.5-3 h. After that, the cultures were supplemented with 500 µL of new growth media and kept being fed twice per week until the experimental use.

#### 4.3) Photostimulation systems

For photostimulation of ChR2, we utilized a 405 nm laser diode (LP405C1, 30 mW, Thorlabs). The laser power was regulated using a laser diode driver (LDC210C, Thorlabs) and a neutral density filter set (N.D. 1-3, NEK01, Thorlabs). The laser light passed through a single band pass filter (FF01-406/15-25, Semrock) and was then reflected on dichroic beam splitters (FF495-Di03, Semrock) and (FF573-DiO1, Semrock). For the excitation of jRGECO1a, a 561 nm laser (MGL-FN-561-50mW, Ultralasers) was used, and the laser passed through a single band pass filter (FBH560-10, Thorlabs), and the dichroic beam splitters (FF4590-Di03) before being reflected on a dichroic beam splitter (FF573-DiO1, Semrock). A fiber collimator (F220FC-A, Thorlabs) was used to direct the beam into the fiber end. The fluorescence of jRGECO1a collected through the fiber propagated back and was collimated by the collimator before passing through the dichroic beam splitter. The fluorescent light was filtered using an edge filter (BLP01-568R) and focused on a PMT detector (photosensor module H6780-20, Hamamatsu) using a plano-convex lens (focal length = 50 mm, LA1131-A, Thorlabs). To prevent external light interference, the entire optical system was enclosed in a light isolator box. The PMT output was then amplified with a gain of 10^6^ and filtered with a numerical filtering followed by a fast Fourier transform low pass filter at 25 Hz (Supplementary Fig. 20). The output power at the tip of the MLF and MFCF in the range of µW and milliwatts/mm^2^ was given by (P_out_ × T_ND_)/(πr^2^), where P_out_ is the laser output power measured at the end of the tip without neutral density (ND) filter, T_ND_ is the transmission coefficient of the ND filters, and r is the radius of the beamwidth at the focal plane of the MLF or MFCF.

#### 4.4) Patch-clamp electrophysiological recordings

Whole-cell patch-clamp recordings were performed on the cortical neuronal cultures at least 8 days after the viral transfection (DIV17-DIV22). The cultures were recorded using a circulating HEPES-buffered Ringer’s solution (2 mL/min) containing (in mM): 126 NaCl, 2.5 KCl, 2 MgCl_2_, 2 CaCl_2_, 10 glucose, 10 HEPES (pH adjusted to 7.35). Whole-cell patch-clamp recordings were performed using borosilicate patch pipettes with a resistance of 3 to 4 MΩ when filled with a solution containing (in mM): 119 K-methylsulfate, 1.7 MgCl_2_, 4.25 KCl, 8.5 HEPES, 3.4 ATPNa, 0.35 GTPNa (pH 7.2 adjusted with KOH). The neurons efficiently expressing ChR2 or JRGECO1a fluorescence were targeted for the recordings, and only neurons with stable access resistance (Ra) lower than 20 MΩ and good physiological properties were used for further analysis.

Once the patch clamp was effectively established and on open configuration, the optical probe was gently placed close to the soma or neuronal proximal dendrites. During the recordings, the neurons were illuminated with light at 405 nm (9 Hz train of 15 pulses with 60 ms duration) and/or 561 nm (continuous wave, 30 s duration) using the MLF. Data were low‑pass filtered during acquisition at 3 kHz and sampled at 10 kHz using a Multiclamp 700B amplifier and pClamp11 (Molecular Devices).

#### 4.5) In vivo “all-optical” single-cell stimulation and recording

The all-optical single-cell stimulation and recording were performed in the CA1 of the hippocampus or the medial prefrontal cortex (mPFC) layer 2/3 of male adult C57BL/6 J mice previously transduced with jRGECO1a and ChR2-EYFP using the 2ST-MLF (18–21 weeks old at the time of recording). Briefly, mice were anesthetized with 1.5-3% isoflurane and placed in a stereotaxic frame (Stoelting Co.) on a heating pad. Under aseptic conditions, two craniotomies were drilled to target the CA1, at Rosto/Caudal (R/C) −2.3 mm and Medial/Lateral (M/L) ± 2.0 mm from Bregma, or the mPFC at R/C + 0.8 mm, and M/L ± 0.3 mm, bilaterally. 400 nL of a 1:1 mix of the AAV9.hSyn.jRGECO1a and the AAV2/1-hSyn-hChR2(H134R)-EYFP were injected into the CA1 or the mPFC using a glass capillary and a NANOLITER2020 injector (World Precision Instruments LLC) at Dorso/Ventral (D/V) 1.5 mm. Recordings started at least 5 weeks after the viral injection.

On the day of the recording, mice were anesthetized with a mix of 100 mg ketamine, 15 mg xylazine, and 2.5 acepromazine per kg and placed in a stereotaxic frame as before. A craniotomy was drilled again at the same coordinates, targeting the CA1 or the mPFC. A 2ST-MLF was lowered vertically into the brain.

The photostimulation and recording of jRGECO1a were performed using the same optical setup used for the fluorescence microbead test. A set of different neural density filters was employed to regulate the strength of the light stimulus, in combination with adjustment of the driving current of the laser diode driver.

For comparison of sensitivity and axial spatial resolution between 2ST-MLF and MFCF, measurements were performed at adjacent locations within the same animal using a small lateral or anterior–posterior offset ( ~ 100 μm), exceeding the probe diameter ( < 25 μm), to minimize interference from the previous insertion site. For sensitivity characterization using 2ST-MLF alone, representative data are shown.

### Invasiveness of fiber photometry

Stereotaxic surgeries were performed as described above. Two craniotomies were drilled at Rosto/Caudal (R/C) − 0.2 mm and Medial/Lateral (M/L) ± 2.0 mm from Bregma, targeting the left and right hemisphere striatum, respectively. The mice ages were 35–42 week old. The 2ST-MLF array and the FCF were lowered 3-4 mm into the one and the other craniotomy within the same animal to minimize inter-animal variability during comparison of tissue response. The fibers were glued in place (Loctite Super Glue), and the skull was covered with C&B Metabond® (Parkell Inc.). Following the surgery, mice were single-housed and sacrificed for analysis 7 days later.

To assess the inflammatory response and tissue damage around the fibers, we performed immunofluorescence analysis for astrocytic and microglia markers. Briefly, the implanted mice were anesthetized with 30% urethane in saline and perfused intracardially with 0.1 M PBS, followed by 4% paraformaldehyde in PBS. Brains were excised and stored in the same fixative for 15 hrs and then in 30% sucrose in PBS. Coronal sections of the striatum were cut 60 μm with a Leica Vibratome VT1220S (Leica Microsystems). Sections were first permeabilized in PBS with 0.2 % Triton X-100 (PBST) and then incubated with the chicken polyclonal anti-GFAP (1:2000, Abcam ab4674), the rabbit polyclonal anti-Iba1 (1:2000, Wako 019-19741) and the mouse monoclonal anti-NeuN (1:1000, Millipore MAB377) diluted in PBST containing 10 % normal goat serum. After washing in PBST, sections were incubated with the secondary antibodies AlexaFluor™ 647-conjugated goat anti-chicken (1:500, Invitrogen A21449), AlexaFluor™ 488-conjugated goat anti-rabbit (1:500, Invitrogen A11008) and AlexaFluor™ 405-conjugated goat anti-mouse (1:500, Invitrogen A31553) for 2 hours, rinsed in PBS, and mounted on SuperFrost™ slides (Thermo Fisher Scientific 12-550-15) using fluorescence mounting media (Agilent S302380).

Tile scans of the sections were acquired at 12 bits and 1024×1024 pixels (with 1.785 μm pixel size) with a Zeiss LSM710 laser scanning microscope using a 5x EC-Plan-Neofluar objective (0.16 NA) objective. All acquisitions were performed using the same settings (laser power, photomultiplier tube settings, and scanning time). Quantification of fluorescence intensity for GFAP and Iba1 was performed with ImageJ within selected Regions of Interest (ROIs) below the implants’ insertion sites. No additional data exclusions were applied during fluorescence quantification.

### Numerical calculation of radiation and collection profiles

The wave optics and ray tracing modules in COMSOL Multiphysics were employed to compute the radiation and collection profiles of the MLF and MFCF, respectively. The refractive indices of the optical cores and claddings were assigned as 1.46 and 1.443, respectively. The fibers were surrounded by either air (*n* = 1) or water (*n* = 1.33)^82^. In our simulations, the incident light had a wavelength of 488, 561, or 980 nm and a power of 1 W. For the MLF, we based the fiber’s geometry on the SEM image shown in Figs. 1, 3–7 with a fixed core diameter of 10 μm across all the simulations. As for the FCF, the cladding thickness was set at 5 μm. To analyze the collection profile, we evaluated the ratio of the guided and collected rays through the 3D optical core to the emitted rays (5000) from the point source at the tip of the fiber, as illustrated in Supplementary Fig. 6c.

### Reporting summary

Further information on research design is available in the Nature Portfolio Reporting Summary linked to this article.

## Supplementary information

Supplementary Information

Description of Additional Supplementary Files

Supplementary Video 1

Supplementary Video 2

Supplementary Video 3

Supplementary Video 4

Supplementary Video 5

Reporting Summary

Transparent Peer Review file

## Source data

Source Data for Main Figures

Source Data for Supplementary Information

## Data Availability

Data supporting the findings of this study are provided in the main article, Supplementary Information, and Source Data files provided with this paper. Data necessary to interpret, verify, and reproduce the reported findings are available through these materials. Source data are provided in this paper.

## Code availability

MATLAB codes implementing the physics-separated multiphysics framework and the geometry-agnostic axial rounding metric have been deposited in Code Ocean (https://doi.org/10.24433/CO.9453164.v1).
