# Supplementary material for: Deterministic wet etching of aspheric fiber microlenses with tunable conic geometry for tailored optical functionality
Source: Nat Commun. 2026 Aug 7;17:9569. doi: 10.1038/s41467-026-75331-y (PMC13550405; doi:10.1038/s41467-026-75331-y)
Supplement: Supplementary file 1 [file 41467_2026_75331_MOESM1_ESM.pdf]

Supplementary Information

Deterministic Wet Etching of Aspheric Fiber Microlenses with Tunable Conic Geometry for Tailored Optical Functionality

Sanggon Kim[1,2,], Iason Keramidis[2], Isabel Plasencia-Fernandez[2], Johanna Alonso[2], Louison Brochoire[2], Cyril Bories[2], Annie Barbeau[2], Younes Messaddeq[1,2,*], and Yves De Koninck[1,2,3,*]*

Addresses:

1 Center for Optics, Photonics and Lasers (COPL), Department of Physics, Université Laval, Québec, QC G1V 0A6, Canada

2 CERVO Brain Research Centre, Quebec Mental Health Institute, Québec, QC, G1J 2G3, Canada

3 Departments of Psychiatry & Neuroscience and Anesthesiology & Intensive Care, Université Laval, Québec, QC, G1V 0A6, Canada

Corresponding author information: Younes.Messaddeq@copl.ulaval.ca, yves.dekoninck@neuro.ulaval.ca, sanggon.kim.1@ulaval.ca

Supplementary Note 1. Characterization of sensitivity and spatial resolution using fluorescent microbeads and neuronal axon branches

To evaluate the practical sensing performance resulting from the combined emission and collection characteristics of the MLF, we compared MLF and MFCF performance under application-relevant conditions using fluorescent microbeads and *ex vivo* imaging of neuronal axon collaterals.

Spatial resolution was first quantified using a 1 µm fluorescent microbead embedded in agar gel (Supplementary Fig. 8). Lateral and axial scanning yielded spatial resolutions of 2.9 µm and 3.1 µm, respectively, corresponding to an effective sensing volume of 13.7 μm^3. Compared with the tapered MFCF, this represents improvements of 130% and 250% in lateral and axial resolution, respectively (Supplementary Fig. 8b,d). In addition to improved spatial confinement, the MLF produced photomultiplier tube (PMT) signals more than 25× stronger than those obtained using a flat-cleaved fiber under identical excitation conditions, demonstrating that enhanced spatial selectivity was achieved without compromising signal sensitivity.

To evaluate performance in biologically relevant structures, the sensing capability was further examined by scanning micron-scale axon collaterals in *ex vivo* brain slices (Supplementary Fig. 9). Compared with the MFCF, the MLF resolved distinct fluorescence peaks corresponding to individual neuronal branches that were not distinguishable using the broader sensing profile of the MFCF. These results demonstrate that the improved optical confinement achieved through deterministic tip shaping translates into enhanced practical sensitivity and spatial resolution in structurally complex environments.

Supplementary Note 2. *In vitro* verification of MLF for "all-optical" interrogation of neural activity beyond the cellular level

To verify that the fluorescence fluctuations observed during *in vivo* tests were solely attributable to Ca^{2+} dynamics and optogenetically induced changes under MLF stimulation for all-optical interrogation of single-cell dynamics, we performed photo-activation and Ca^{2+} transient recordings on a cultured single cell expressing both ChR2-EYFP and jRGECO1a using microscopy (Supplementary Fig. 14a). We first captured fluorescence images of the cells under wide-field illumination using 470 nm and 530 nm microscope LED light sources, confirming the presence of optogenetics (Supplementary Fig. 14b) and Ca^{2+} fluorescent indicators (Supplementary Fig. 14c). Subsequently, a MLF tip was positioned near the cell, emitting a 561 nm laser with a power density of 3 mW/mm^2. A 405 nm laser, coupled through the same fiber at 20 mW/mm^2, recorded fluorescence fluctuations. To mitigate fiber autofluorescence caused by the 405 nm laser, the photoactivation process involved a series of 9 Hz light pulses, each 60 ms long, with a 5-second

delay between pulses[1]. This power density is well below the photodamage threshold of 405 nm laser exposure (200 mW/mm²)[2]. Post-photoactivation, the PMT outputs (Supplementary Fig. 14f) were processed using numerical and low-pass filters to remove autofluorescence peaks (Supplementary Fig. 20).

During experiments, we initially targeted the soma of a hippocampal neuron to stimulate and record neural activity, followed by directing the MLF tip towards a dendrite far from the soma, demonstrating the fiber's capability to activate and record neural signals from dendrites (Supplementary Fig. 14e). ChR2 activation induced noticeable PMT output changes, monitoring fluorescence fluctuations associated with Ca^{2+} influx with a substantial SNR (Supplementary Fig. 14f,g). Despite the dendritic signal amplitude being 32% of that observed in the soma, it remained distinctly detectable, highlighting the MLF's unique ability to capture neural activity from solitary dendrites, a significant achievement in fiber photometry.

Further investigations confirmed the absence of fluorescence intensity changes upon 405 nm photostimulation of cells expressing only ChR2 (Supplementary Fig. 14h). In cells expressing only jRGECO1a, transient fluorescence changes were observed during 405 nm illumination onto the soma, but the amplitude was about 20% of that in cells co-expressing ChR2 and jRGECO1a (Supplementary Fig. 14i), indicating there is a slight crosstalk.

To understand the responses of ChR2 and jRGECO1a to 561 nm and 405 nm light, we used a patch clamp technique to monitor intracellular current and membrane potential changes under these specific wavelengths (Supplementary Fig. 15a). First, we focused on assessing ChR2's response to continuous 561 nm and pulsed 405 nm laser illumination independently in hippocampal neurons expressing only ChR2. ChR2 expression was confirmed by detecting EYFP fluorescence under 470 nm wide-field illumination (Supplementary Fig. 15b). We monitored the intracellular current while illuminating the cells using a MLF (Supplementary Fig. 15c). ChR2 showed a negligible response to 561 nm light, but a significant current change occurred with 405 nm light exposure, demonstrating ChR2's specific activation by 405 nm light (Supplementary Fig. 15d,e).

In a control experiment, we examined whether illumination of the soma or dendrite could induce action potentials (APs) by targeting regions with ChR2. Our findings confirmed that the MLF's focal spot could effectively activate sufficient ChR2 to initiate APs, even when targeting dendrites

(Supplementary Fig. 15g,i). These results support MLF's potential for "all-optical" neural activity interrogation beyond the single-cell level.

To further explore the effects of 561 nm and 405 nm light on cells expressing only jRGECO1a, we conducted intracellular monitoring (Supplementary Fig. 16). Exposing the cells to both lasers showed no significant changes in membrane potential. This result suggests that the fluorescence fluctuations observed in Supplementary Fig. 14h,i under 405 nm illumination in the absence of ChR2 were attributed to jRGECO1a's intrinsic response rather than being caused by Ca^{2+} transients. These findings align with previous reports of minor effects seen with Red GECO[3], though the exact cause remains unclear. Newer indicators such as Lyn-GECO1a, which do not exhibit Ca^{2+}-independent fluorescence increases[3], and FR-GECO1c, characterized by fast rise and decay times, stand out from action potential-evoked Ca^{2+} fluorescence[4] and could help mitigate these effects.

Supplementary Note 3. Numerical calculation of the local temperature increase induced by the absorption of the light by the etchant

Supplementary Note 3.1. Geometry extraction and model construction*:* Fiber geometries used in Multiphysics simulations were directly extracted from high-resolution scanning electron microscopy (SEM) images of experimentally fabricated tapered fibers. SEM images were calibrated using known pixel-to-length conversion factors, and the fiber boundary was manually traced to obtain the two-dimensional surface profile $x(y)$. The extracted profiles were smoothed only to remove pixel-scale digitization noise, without altering the apex curvature or taper angle. These experimentally derived geometries were imported into COMSOL Multiphysics.

Because the fiber geometry and heating profile are axisymmetric about the fiber axis, simulations were performed in a two-dimensional Cartesian domain representing a longitudinal cross-section of the fiber and surrounding liquid. This approach captures the dominant optical absorption and heat-transfer behavior while remaining computationally tractable.

Supplementary Note 3.2. Wave-optics and Heat Transfer Multiphysics Simulation: Optical emission from the tapered fiber and other geometries was simulated using the Wave Optics and Stationary heat-transfer Multiphysics Module in COMSOL. The experimentally extracted fiber geometry was assigned a refractive index corresponding to silica (1.46) at the laser wavelength,

while the surrounding medium was assigned the refractive index and extinction coefficient of water, 1.33 and 7.28×10^{-9} (@ $\lambda = 1550\ nm$) respectively. A scattering boundary was set for all the outer layers, and a 1 μm of perfectly matched layer was set at the port to mitigate the reflection. A guided optical fundamental mode was launched from the fiber core, and electromagnetic field propagation and absorption were solved in the frequency domain. To simplify the thermal model, the solid–liquid interface was treated as thermally insulating. At the outer boundary of the water domain, an effective heat-flux boundary condition was applied:

$$-q = h\,(T - T_{infinite}),$$

where $T_{infinite}$ is the ambient temperature and h is an effective microscale heat-transfer coefficient. The heat transfer coefficient h for this boundary was estimated from the experimentally validated microscale Nusselt correlation reported by Guan et al.[5]:

$$Nu = 4.325(GrPr)^{0.1069},\ \ Nu = \frac{hD_{eff}}{k}$$

To define the characteristic length scale, we extracted the optical heating footprint from the electromagnetic absorption field using $Q_e \geq Q_{e,max}/e$ (Supplementary Fig. 3), yielding $A_{hot} = 8.15 \times 10^{-12} m^2$. An equivalent heating diameter was then defined as $D_{eff} = 2\sqrt{A_{hot}/\pi} \approx 3.6\ \mu m$, and substituted into the correlation. Using ambient condition water properties gave $Nu \approx 1.23$, corresponding to $h \approx 2.05 \times 10^5\ Wm^{-2}K^{-1}$. This value is consistent with reported microscale heater convection magnitude, reflecting efficient heat removal from micron-scale hotspots in water.

We further tested the robustness of this choice by repeating simulations using the coefficient reported by Guan et al. for a larger heater (~30 μm diameter). The resulting temperature difference was within a few degrees (<3 °C) and did not change peak temperature location, temperature-gradient shape, or etching-rate trends. This confirms that the thermal predictions are insensitive to moderate variation in h and validates the use of the upper-bound value as a conservative and physically justified choice for our geometry.

In our simulations, the outer water boundary was positioned >200 μm from the heated region (> $66\times D_{eff}$), ensuring a conduction-dominated regime where the Guan correlation applies. The

boundary distance was selected such that doubling it changed the surface temperature gradient profile by <5%. Because LCWCE is governed by temperature along the fiber boundary, we extracted temperature profiles along the surface anchor coordinate s_{local}(Fig. 2b). Simulations were conducted in 2D; therefore, the optical input power was specified in units of Wm^{-1} and adjusted so that the predicted steady-state temperatures remained in the physically relevant range of 293.15–363.15 K (moderate heating well below boiling).

Supplementary Note 3.3. ***Extraction of surface temperature and etching-rate kernels:*** Temperature profiles were extracted along the fiber surface using the surface anchor coordinate s_{local}, defined as the arc length measured from the apex. Regions near the outer heat-flux boundary, where temperatures relax toward ambient, were excluded from analysis; specifically, anchor lengths beyond the point where $T \leq T_{max}/e$ ($s_{cut} = 256.6\ \mu m$) were omitted to avoid boundary artifacts.

Supplementary Note 3.4. Numerical implementation of staged normal-etching surface evolution: The fiber surface was represented by the experimentally extracted right-hand branch and parameterized using the axial coordinate ζ = -*y*. Prior to time integration, both the surface geometry and the stage-specific normal etching-rate kernels were resampled onto a high-resolution uniform ζ-grid (0.1 µm spacing) to ensure accurate and stable evaluation of surface normals and curvature throughout the evolution. The normal angle used during geometry evolution, $\theta_n(\zeta)$, was defined with respect to the axial coordinate to enforce a monotonic and numerically well-behaved representation during tip rounding. Because the evolution was performed on a half geometry and the apex constitutes a boundary point, extrapolated points were introduced beyond the physical apex to enable smooth derivative estimation and avoid numerical artifacts associated with endpoint differentiation. To further improve numerical stability near the apex, surface normals were optionally evaluated with a small axial offset toward negative ζ, thereby avoiding singular behavior exactly at the apex; this offset does not alter the physical etching trends and is consistent with the finite spatial resolution of the chemical etching process. To eliminate artificial time-step dependence during surface evolution, both the evolving geometry and the applied etching-rate kernels were anchored at the instantaneous apex position (*y*=0) at each iteration. This anchoring procedure ensures that the numerical evolution reflects genuine changes in surface orientation and curvature rather than accumulated coordinate drift, allowing stable simulations without requiring

impractically small time steps. Switching between stage-specific etching-rate kernels was governed by a fixed probe-depth criterion: the instantaneous lateral radius at $x = 5\ \mu m$ was monitored, and transitions between kernels were triggered when this radius reached the corresponding COMSOL-derived values for the intermediate and advanced geometries. This criterion provides a physically motivated and numerically robust mapping between continuous surface evolution and the discrete Multiphysics stages used in the staged approximation.

Supplementary Note 4. Quantitative Conic Analysis of SEM-Derived Tip Profiles

The SEM-derived tip profile was quantified by fitting the axial sag s as a function of radial coordinate r to a conic-section model. Using the apex as the origin, the distance from the apex is defined as $s=-y\geq 0$ and the radius as $r = |x|$. The conic sag equation[6] is

$$s(r) = \frac{r^2/R}{1+\sqrt{1-\frac{(1+K)r^2}{R^2}}},$$

Where R is the radius of curvature and K is the conic constant. R and K were obtained by minimizing the squared residuals between the measured and modeled sag values (nonlinear least-squares). Fit quality was evaluated using the coefficient of determination,

$$R^2 = 1 - \frac{\sum_i (s_i - \hat{s}_i)^2}{\sum_i (s_i - \bar{s}_i)^2},$$

where s_i denotes the measured sag values, $\hat{s}_i$ the fitted values, and $\bar{s}_i$ their mean. Values of R^2 approaching unity indicate excellent agreement with the conic model. In practice, the fits consistently yielded $R^2 \geq 0.995$, confirming the high fidelity of the conic representation for the SEM-derived tip profiles. Because the effective optical interaction is confined to the single-mode fiber core, the fitting was restricted to $r \leq 5\ \mu$m.

Supplementary Note 5. Definition and implementation of the geometry-agnostic axial rounding metric

To quantify how LCWCE redistributes material along the fiber axis without imposing a specific lens classification, we introduce a geometry-agnostic axial rounding metric that captures the spatial extent of etching-induced rounding relative to the initial fiber geometry. This metric isolates axial

redistribution of curvature independent of absolute tip size, fitting window selection, or conic classification, enabling direct comparison of rounding behavior across different initial taper angles.

For a given initial tapered fiber geometry, the local radius profile prior to etching is denoted as $r_0(\zeta)$, where $\zeta \geq 0$, is the axial coordinate measured from the apex along the fiber axis. After application of the etching-rate kernel, the updated radius profile is $r_{etched}(\zeta)$. We define the normalized axial rounding magnitude as

$$M(\zeta)=\frac{r_0(\zeta)-r_{etched}(\zeta)}{r_0(\zeta)}$$

This normalization removes trivial scaling associated with the local cone radius and allows comparison between fibers with different taper angles and absolute dimensions.

The axial propagation length of rounding, L_p, is defined as the axial distance from the apex over which $M(\zeta)$ exceeds a fixed fractional threshold (10% of the maximum value observed near the apex). This criterion provides a robust measure of how far rounding effects extend along the fiber relative to the initial geometry.

For this analysis, a single surface-temperature–derived etching-rate kernel was applied to each initial geometry. The kernel is obtained from steady-state multiphysics simulations of optical heating and temperature-dependent wet etching and is held fixed across geometries. This ensures that differences in $M(\zeta)$ and L_p arise solely from variations in the initial taper angle.

Surface evolution is computed by advancing each surface point along its local outward normal according to the applied etching rate. The resulting radius profiles are then used to calculate $M(\zeta)$ and extract L_p.

The geometry-agnostic metric thus provides a physically transparent and numerically stable means to compare axial rounding behavior across taper angles and to interpret how initial geometry influences the pathway toward different lens shapes.

Supplementary Note 6. Numerical calculation of the optical force exerted on a single polystyrene (PS) particle.

We used the 2D wave optics modules in COMSOL Multiphysics to compute the radiation profiles of the PMLF, MLF, and TF optical tweezers without particles. The refractive indices of the

tweezers and the surrounding medium were set to 1.46 and 1.33, respectively. A 980 nm laser with a power of 1 W was launched into the fiber end port, with a perfectly matched layer positioned behind the port to minimize reflections. For the PMLF, the fiber's geometry was modeled based on the SEM image, while MLF and TF optical tweezers, a taper angle of 9 º was employed, with the MLF featuring a tip radius of 1 μm.

To calculate the optical force (**F**) exerted on the particle, 450 nm PS with a refractive index of 1.57 was placed at the tip of the optical tweezer, changing its position axially and transversally. The optical force **F**, exerted on a particle can be calculated by integrating the time-dependent Maxwell stress tensor **T** around the particle over a closed surface *S* that encloses the particle, expressed as[7,8]

$$\mathbf{F} = \oint_S \mathbf{T} \cdot n dS$$

where **n** is the surface normal vector, and the components *T* are defined as

$$T_{ij} = \epsilon_0 \left(E_i E_j - \frac{1}{2} \delta_{ij} E^2 \right) + \frac{1}{\mu_0} \left(H_i H_j - \frac{1}{2} \delta_{ij} H^2 \right)$$

Where **E** and **H** are the electric and magnetic field vectors, respectively. ϵ_0 is the permittivity of free space, μ_0 is the permeability of free space, δ_{ij} is the Kronecker delta.

The excitation was set to transverse-electric (TE) polarization at a wavelength of 980 nm to calculate the optical force **F** on the particle. The electromagnetic field components are $E_z, H_x, and\ H_y$ were obtained from the 2D simulation. Using these components, the axial force F_y, along the x-direction) and the transverse force (F_x, along the y-direction) exerted on the particle can be calculated using the following expressions[9]:

$$F_x = \frac{1}{2} \oint_S \left[-\frac{1}{2} \left(\epsilon_0 \epsilon_r |E_z|^2 + \mu_0 \mu_r |H_y|^2 - \mu_0 \mu_r |H_x|^2 \right) dy + \mu_0 \mu_r Re\left(H_y H_x^* \right) dx \right]$$

$$F_y = \frac{1}{2} \oint_S \left[-\frac{1}{2} \left(\epsilon_0 \epsilon_r |E_z|^2 + \mu_0 \mu_r |H_x|^2 - \mu_0 \mu_r |H_y|^2 \right) dx + \mu_0 \mu_r Re\left(H_y H_x^* \right) dy \right]$$

Supplementary Note 7. Statistics and Reproducibility

Details of statistical analysis and reproducibility assessment are described below. Optical microscope, SEM, and fluorescence images shown throughout the manuscript are representative of independently prepared samples. Fiber structures and fluorescent images were characterized from a single side view unless otherwise stated.

Figs. 1 and 3 present geometry evolution obtained through systematic parameter mapping rather than repeated measurements under identical conditions. For time-dependent studies (Fig. 1), independent fibers were fabricated and processed for predefined etching durations and characterized after completion of etching. For taper-angle-dependent studies (Fig. 3), fibers with different initial taper angles were independently prepared and processed under identical LCWCE conditions except for etching duration. Representative optical and SEM images are shown to illustrate geometry evolution under each condition. These experiments were designed to establish deterministic relationships between processing parameters and resulting geometry rather than estimate variability for every individual condition.

Independent reproducibility was evaluated separately under selected processing conditions (Supplementary Fig. 2). Six independently tapered fibers with matched initial geometries were processed under identical LCWCE conditions using freshly prepared etchant. Similar geometry evolution and final tip characteristics were observed across independent preparations.

For beam-width characterization (Fig. 1d), fluorescence emission profiles were measured at two orthogonal rotational orientations of a single MLF and averaged at the focal plane to improve measurement accuracy. Emission and collection profile characterizations in other figures were performed using a single rotational orientation because these measurements were less sensitive to rotational alignment and did not require subwavelength spatial resolution.

Supplementary Figures

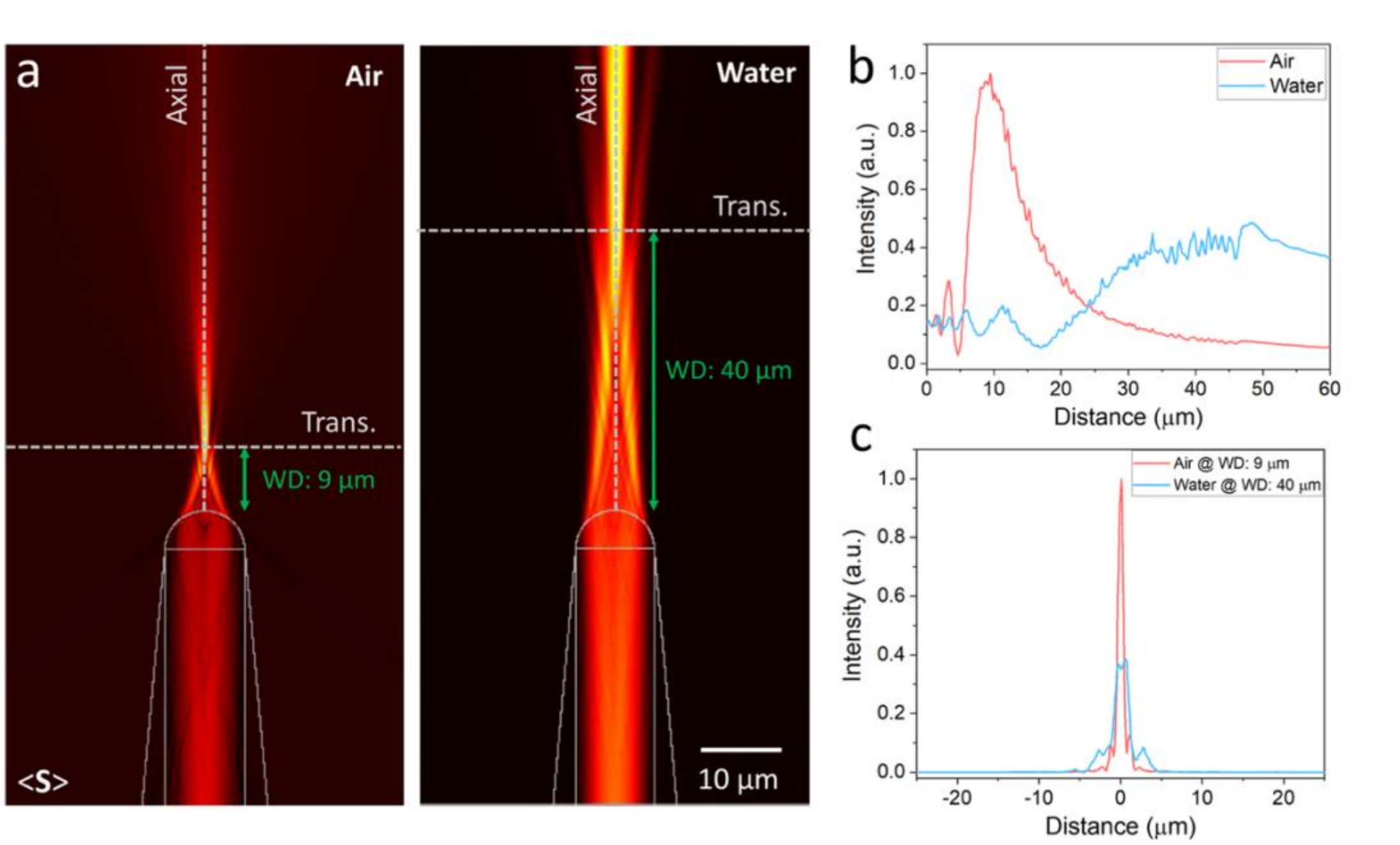

Supplementary Figure 1. Radiation patterns of a spherical microlensed fiber in air and water. (a) Poynting vector flow (<**S**>) of light emitted from the spherical fiber tip in air and water. Incident light wavelength: 561 nm. (b-c) Axial (b) and transversal (c) emission profiles along the dashed lines in (a). WD: Working Distance

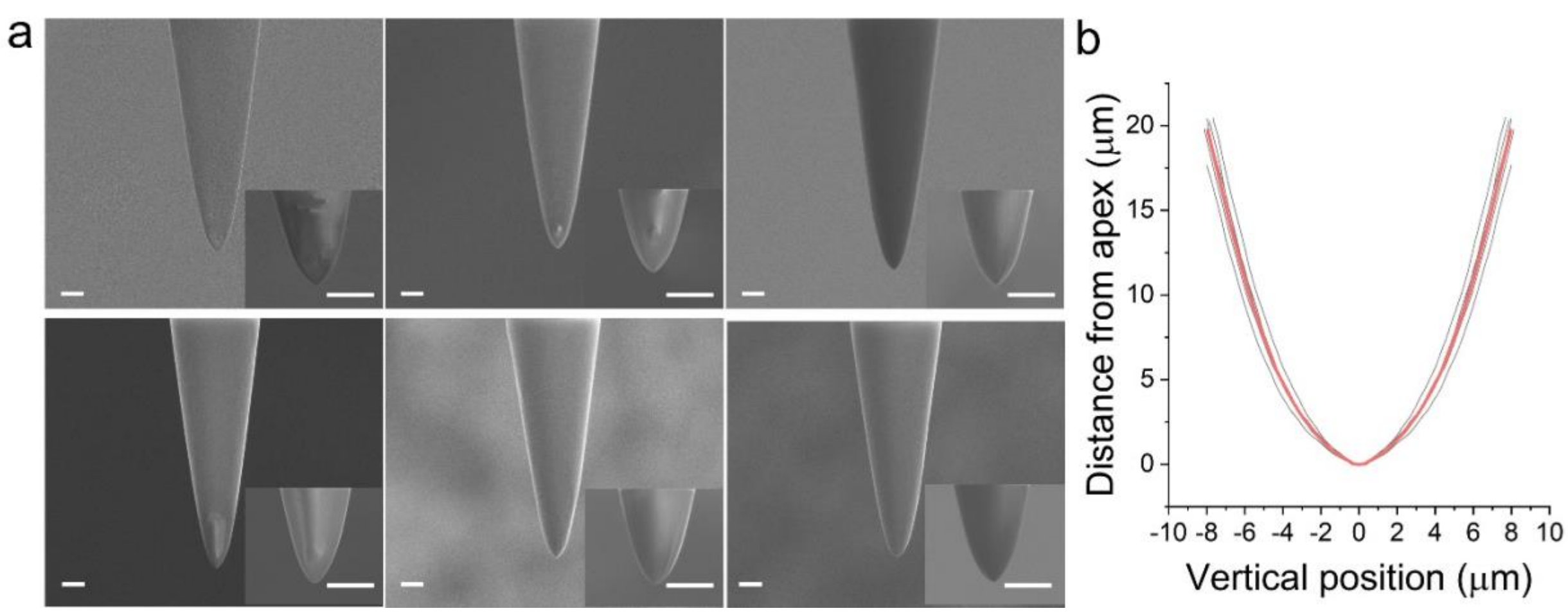

Supplementary Figure 2. Micro-lensed fiber tips fabricated in six independent laser-controlled wet-chemical etching (LCWCE) runs under identical conditions to assess reproducibility. The average tip radius of curvature is 1.49 μm with a standard deviation of 0.24 μm. The scale bar is 10 μm.

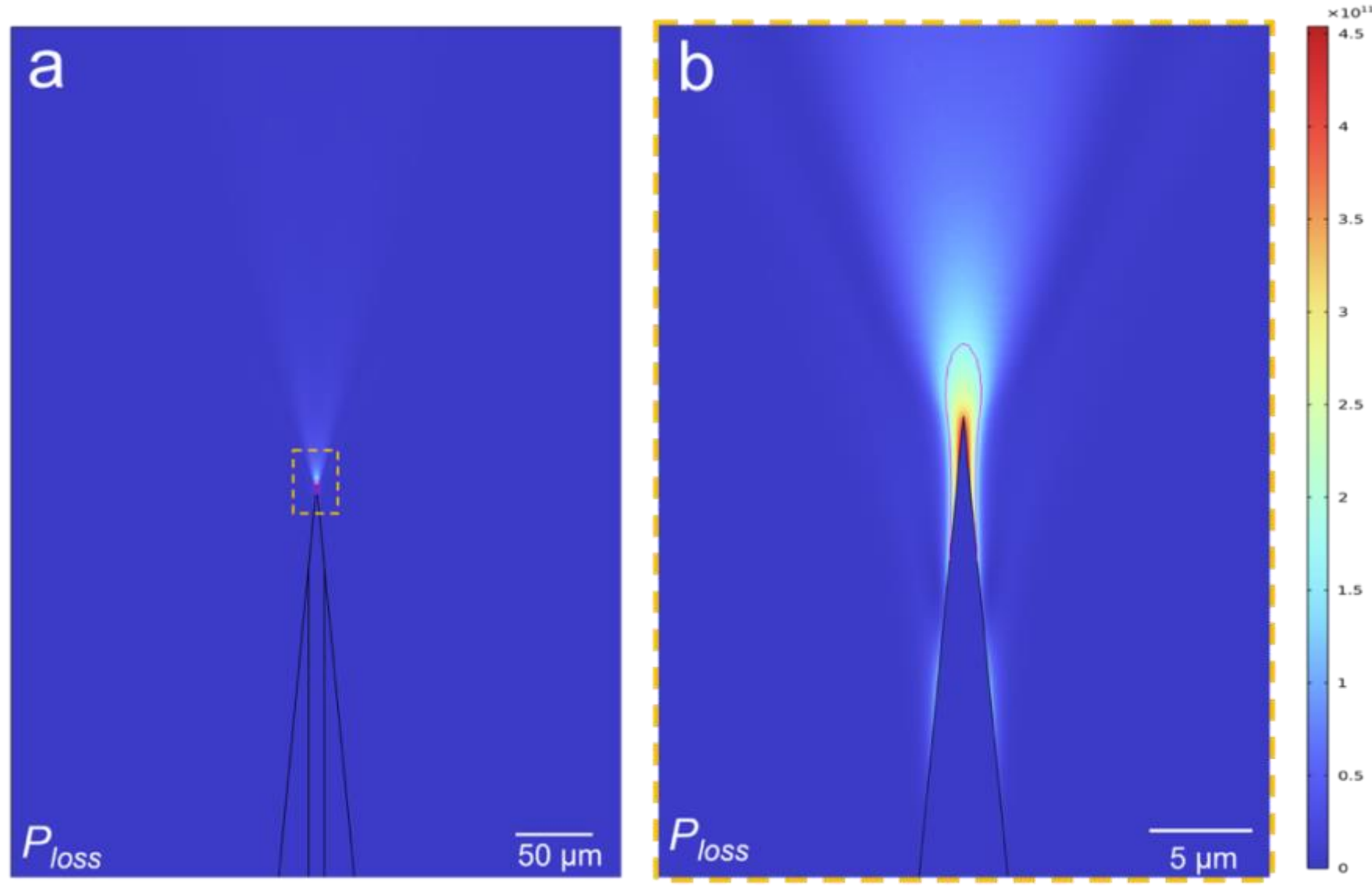

Supplementary Figure 3. Electromagnetic power loss distribution used to define the effective heating area and microscale heat transfer coefficient. Two-dimensional electromagnetic simulation showing the spatial distribution of optical power loss density P_{loss} in water induced by 1550 nm light emitted from the tapered fiber tip. (a) full simulation domain illustrating the strong localization of energy dissipation near the fiber apex. (b) magnified view of the boxed region highlighting the confined heating zone adjacent to the tip. The contour delineates the region, where $P_{loss} \geq {}^{P_{loss,max}}/_{e}$, which is defined as the effective optically heated area A_{hot}.

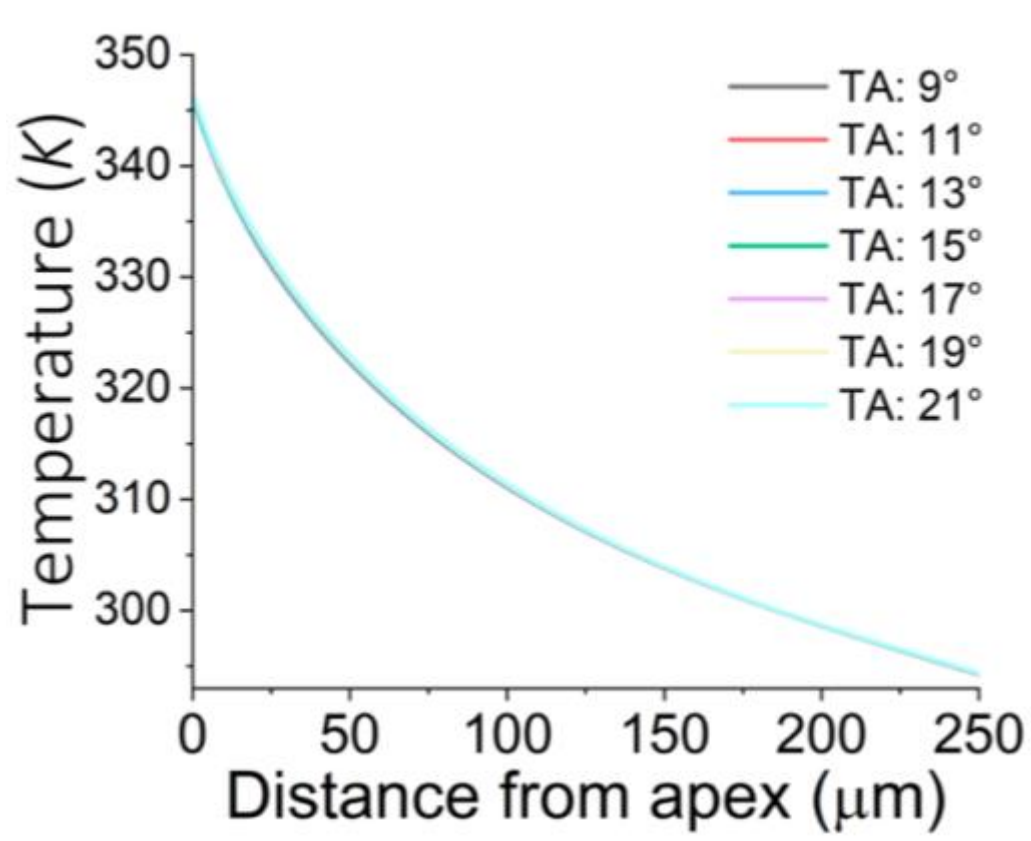

Supplementary Figure 4. Surface temperature $T(s_{local})$ as a function of distance from the apex for fibers with varying taper angles (TAs), demonstrating negligible dependence of the thermal profile on taper angle.

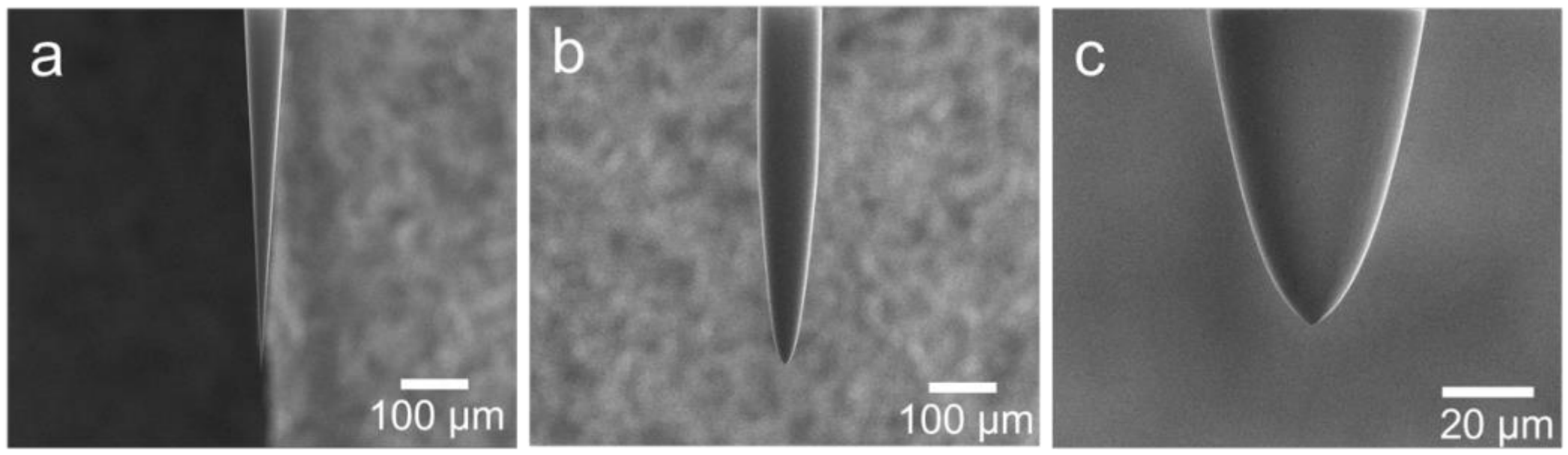

Supplementary Figure 5. Representative micro-lensed multimode fiber with a 105 μm core diameter, fabricated by laser-controlled wet-chemical etching (LCWCE) with a small initial taper angle. (a-c) SEM images of the fiber tip structure before (a) and after (b-c) the LCWCE. The initial taper angle is 6.5°. Laser power and etching time: 500 mW for 25 min in 10% hydrofluoric acid.

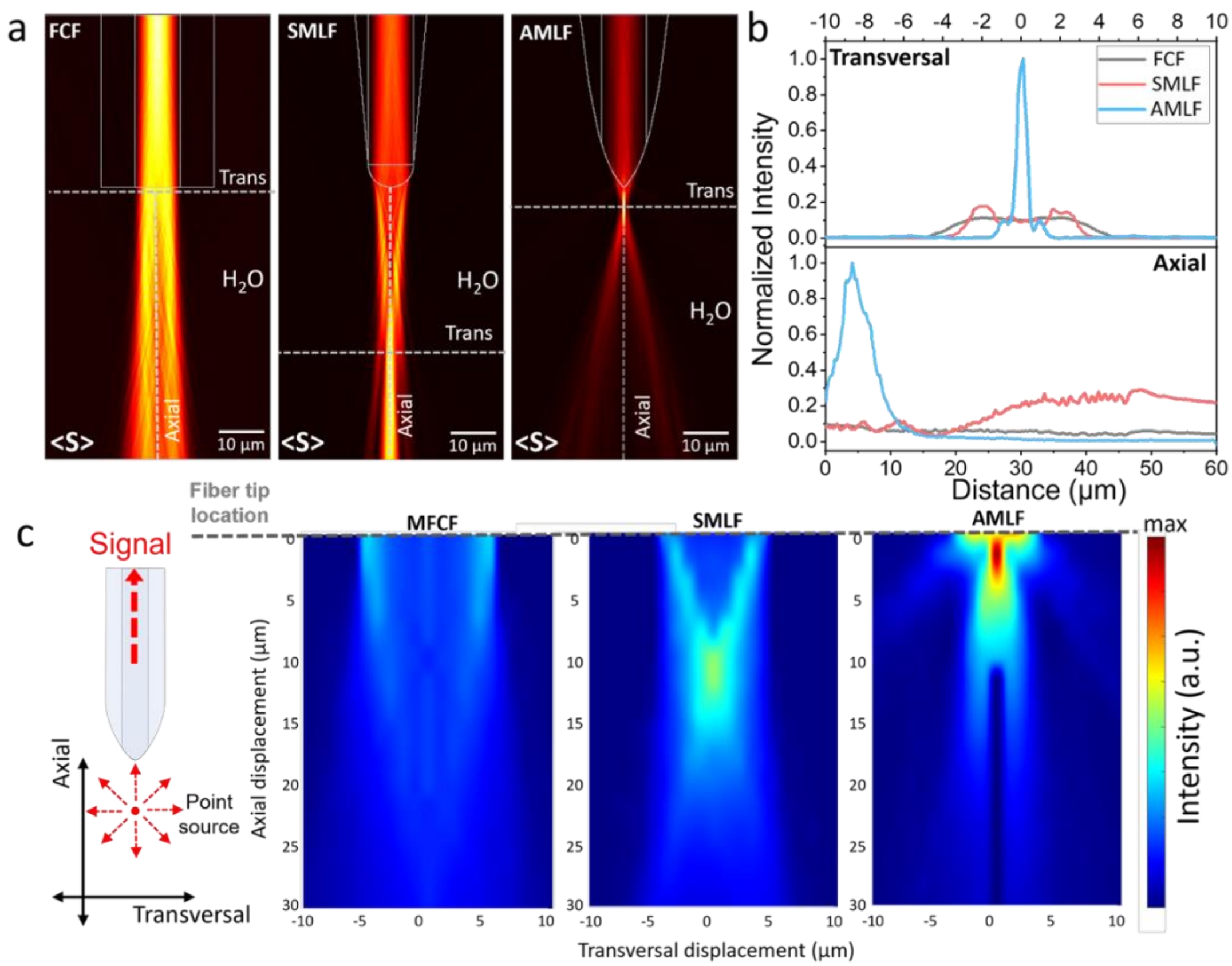

Supplementary Figure 6. Superior focusing and collection capabilities of aspheric microlensed fiber (AMLF) in a high-refractive-index medium compared to micro flat-cleaved fiber (MFCF) and spherical MLF (SMLF), simulated using COMSOL Multiphysics. (a) Beam profile of light emitted from a microprobe with different tip structures in water. (b) Axial and transversal intensity distribution at the focal plane in (a). (c) Collection profiles of rays emitted from a point source at the tip of a microprobe with different tip structures. λ_{ex}: 488 nm.

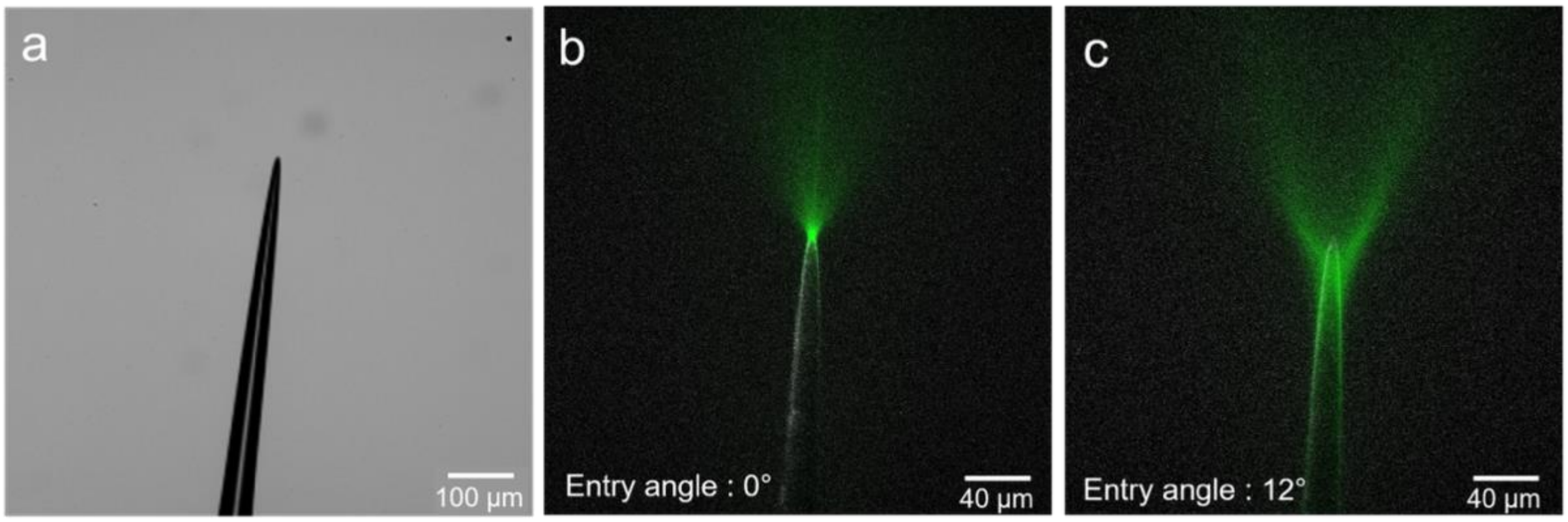

Supplementary Figure 7. Dynamic control of emission profile from a MLF by varying the light entry angle at the input end of the fiber. Representative optical microscope images of the MLF (a) and the light emission profile of the fiber with a different entry angle (b and c). λ_{ex}: 488 nm. Fluorescent dye: 50 μM Fluorescein in water.

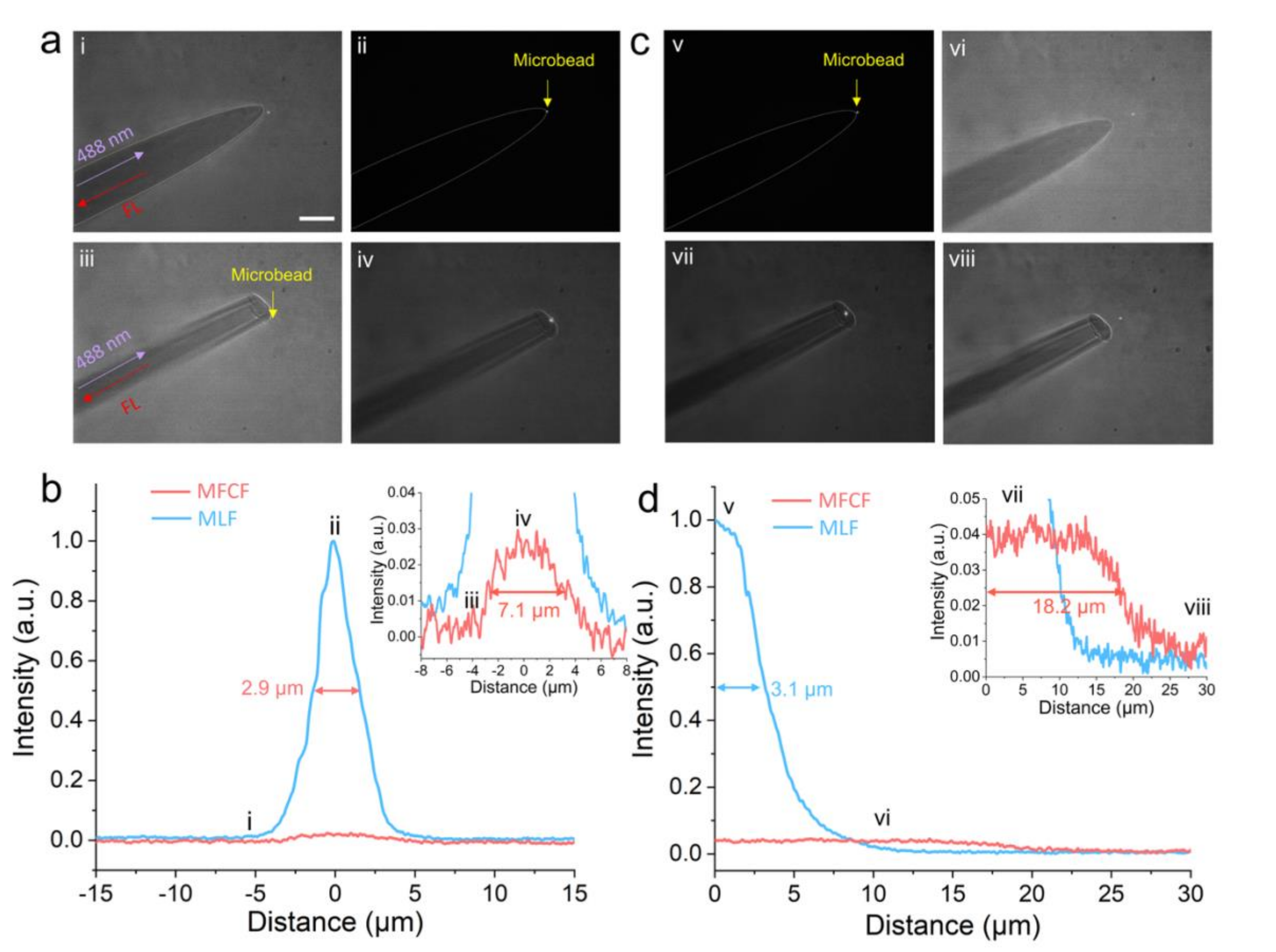

Supplementary Figure 8. Comparison of fluorescent signal intensity and spatial resolution between microlensed fiber (MLF) and micro flat-cleaved fiber (MFCF). (a) Representative optical microscope images of the fibers and a fluorescent microbead taken during the transversal probe displacement. (b) Representative fluorescence intensity profile as a function of transversal probe displacement around a single microbead. The inset shows a zoomed-in fluorescence intensity profile for the MFCF. Labeled points (i–iv) correspond to the images in (a). (c) Representative optical microscope images of the fibers and a microbead captured during axial probe displacement. (d) Representative fluorescence intensity profile as a function of axial probe displacement around a single microbead. The inset shows a zoomed-in fluorescence intensity profile for the MFCF. Labeled points (v–viii) correspond to the images in (c). Scale bar: 30 µm. Excitation wavelength and output power: 488 nm and 30 µW.

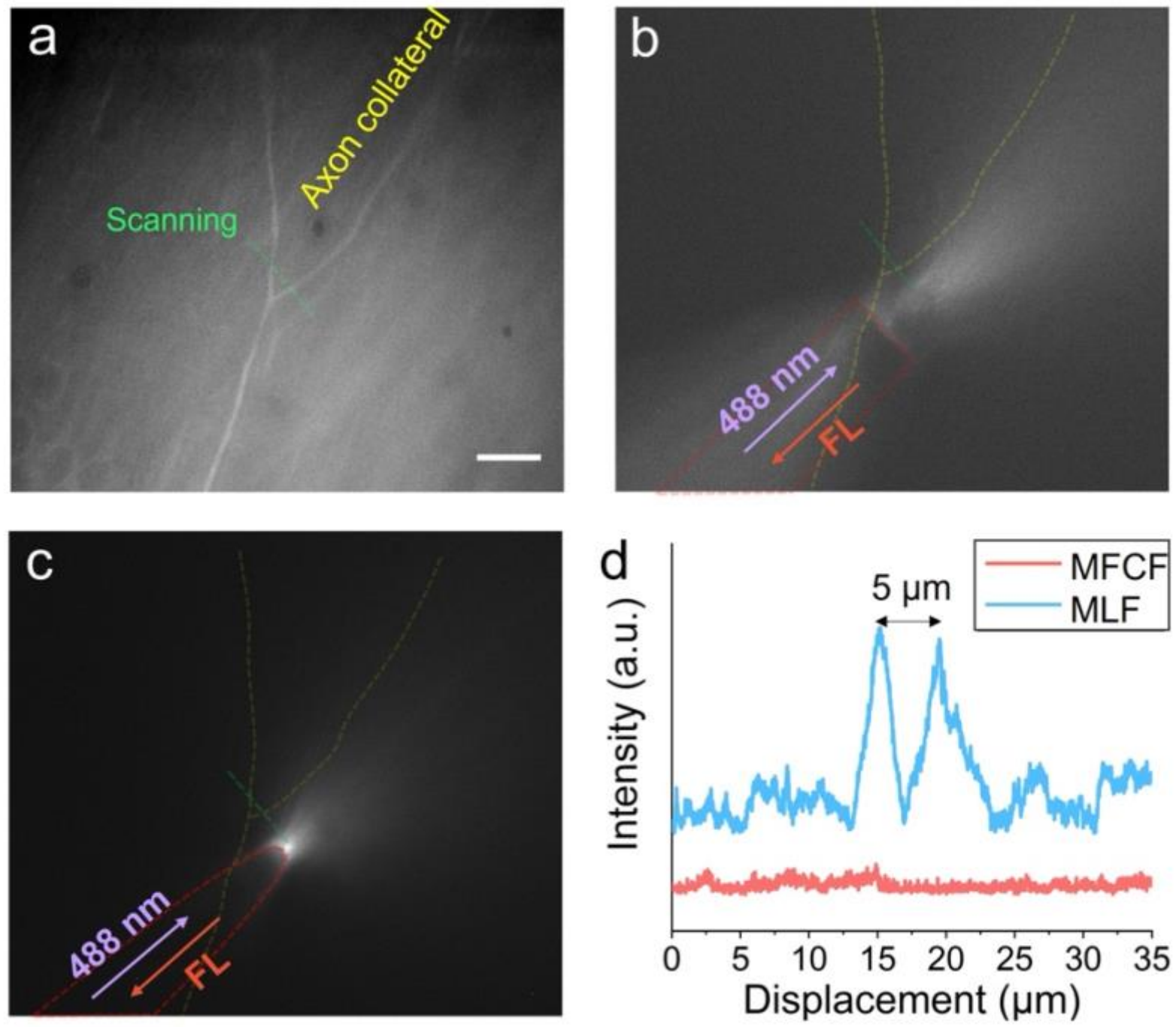

Supplementary Figure 9. Axon collateral sensing using micro flat-cleaved fiber (MFCF) and microlensed fiber (MLF). (a) Representative EYFP fluorescence image of an axon excited by a 470 nm LED through a 40× water immersion objective lens. (b-c) Representative emission from MFCF (b) and MLF (c) upon fiber-coupled laser excitation (488 nm) and corresponding EYFP fluorescence emission. The fiber tip was moved along the green dashed line in (a). (d) photomultiplier tube (PMT) output along the green dashed line. Scale bar: 20 μm. FL: Fluorescence.

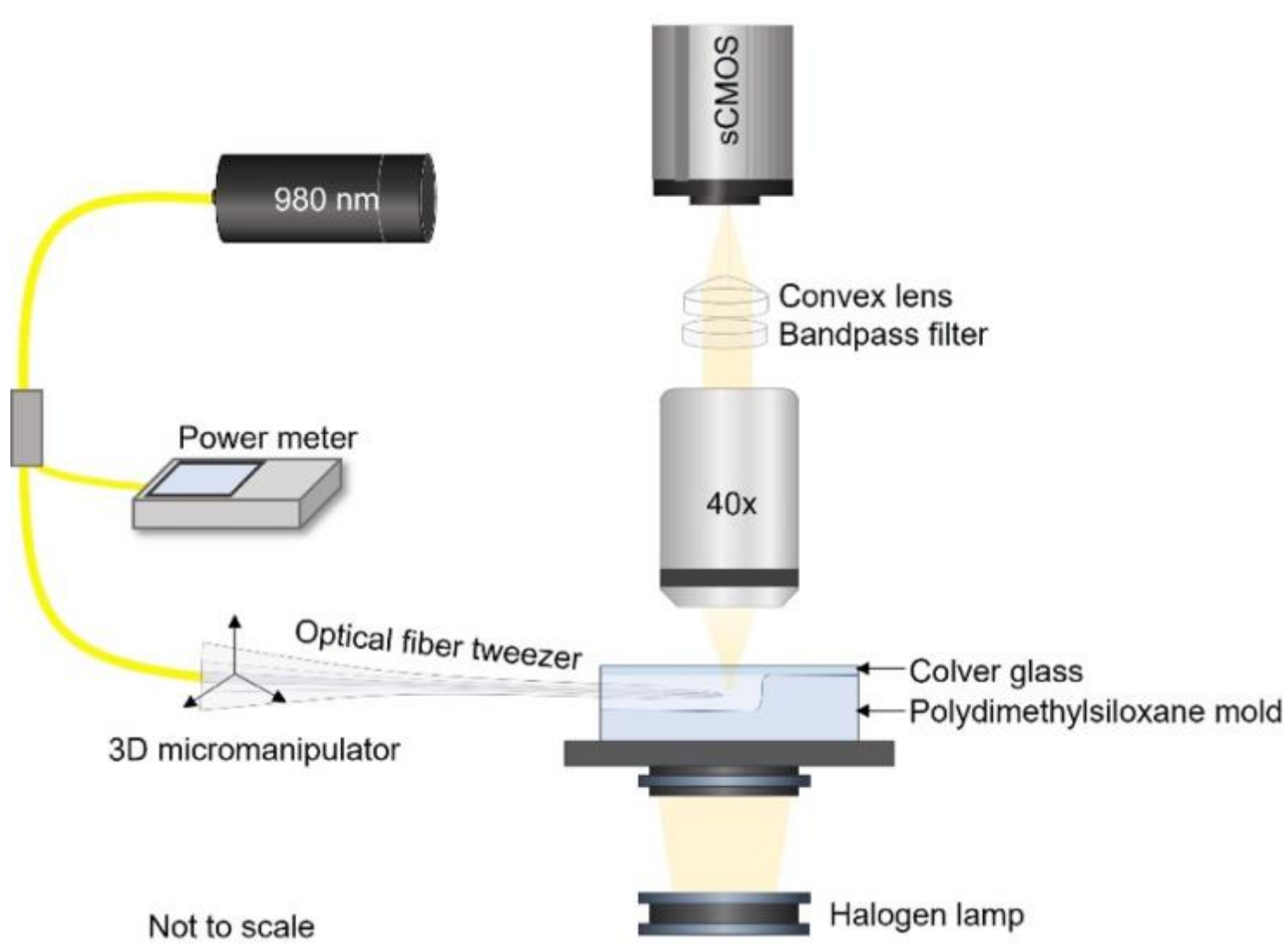

Supplementary Figure 10. Schematic illustration of the optical set-up to trap borosilicate microbead and polystyrene nanoparticles.

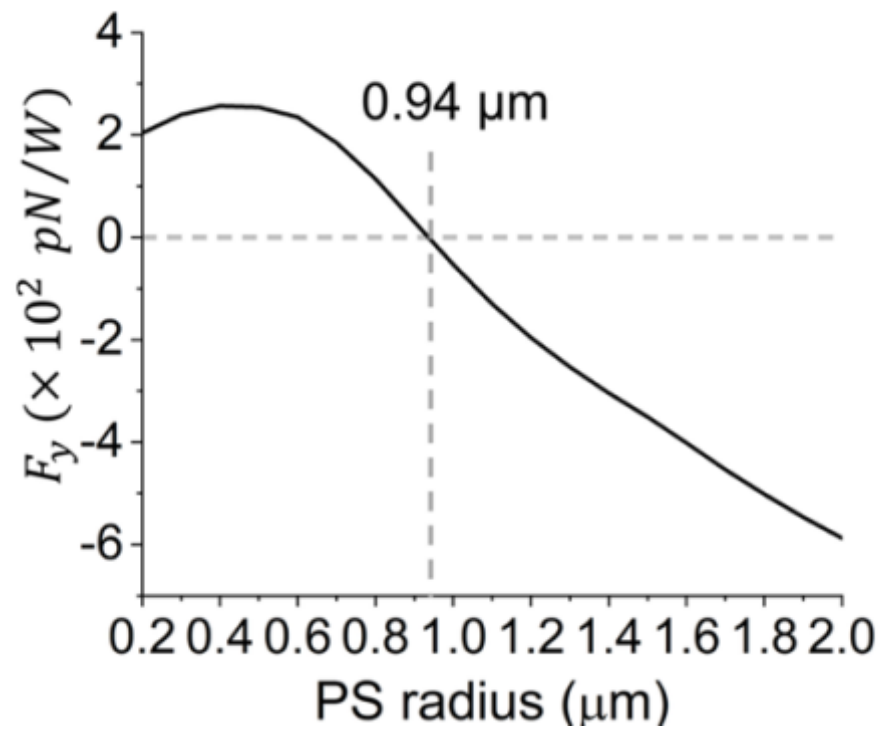

Supplementary Figure 11. Optical force (F_y) of the spherical micro-lensed fiber (SMLF) as a function of the polystyrene (PS) particle radius.

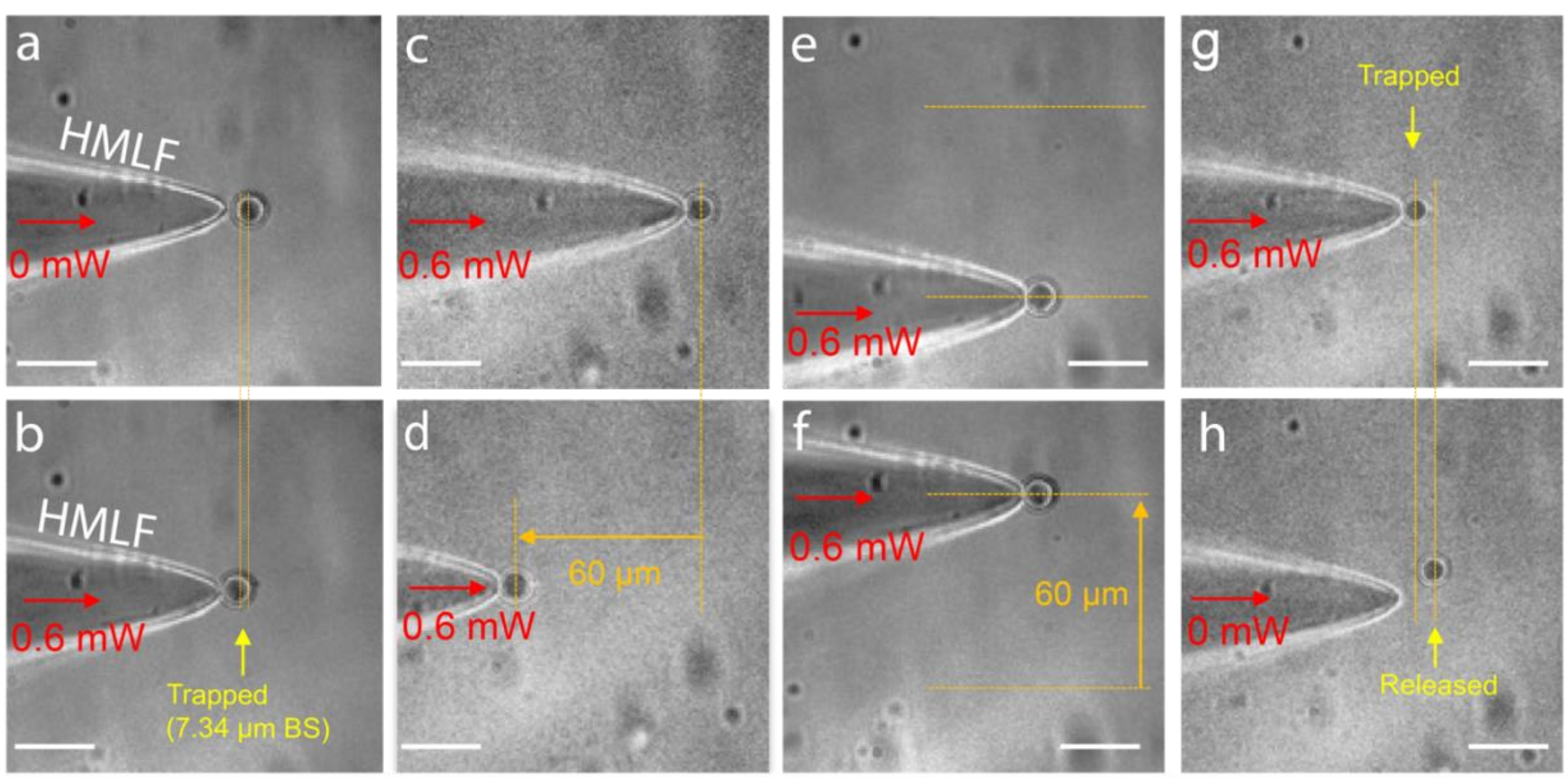

Supplementary Figure 12. Representative trapping strength of the precision hyperbolic micro-lensed fiber (HMLF) for microbeads (7.34 µm borosilicate microbead (BS)). (a-b) Trapping of a silica bead using a 980 nm laser at 0.6 mW. (c-d) Axial movement of the bead using the HMLF with an output power of 0.6 mW. (e-f) Transversal movement of the bead using the HMLF with an output power of 0.6 mW. (g-h) Release of the silica bead from the tip upon turning off the laser. Scale bar: 20 µm

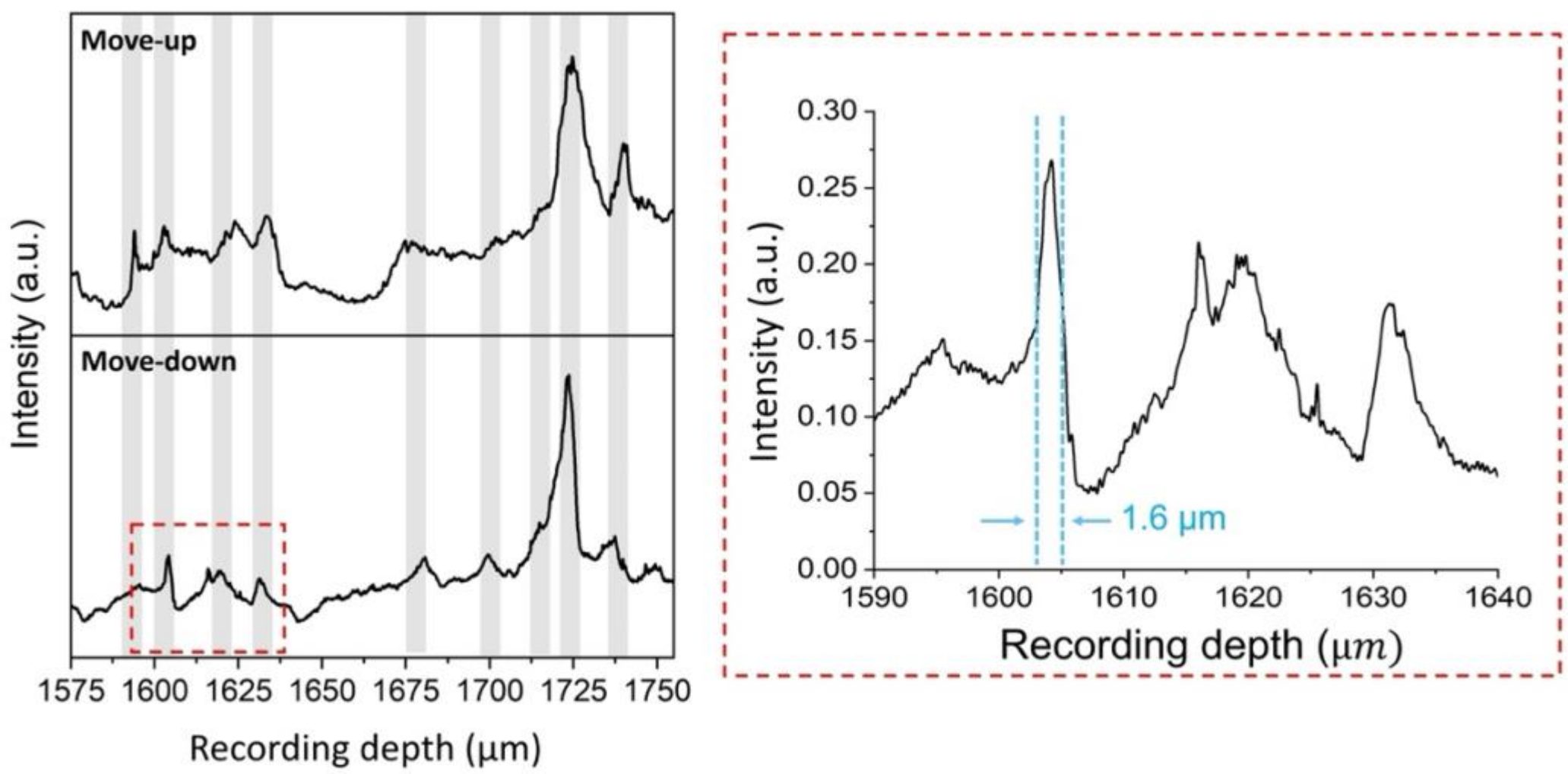

Supplementary Figure 13. Repeated observation of fluorescent peaks (jRGECO1a, λ_{ex}: $561\,nm$ (3mW/mm^2)) during probe movement, demonstrating the effectiveness of the small, smooth, and rounded PMLF tip. This design enables gentle cell displacement without causing damage as the probe passes. The enlarged image in the red box highlights the exceptional capability of identifying single-neuron branches *in vivo*.

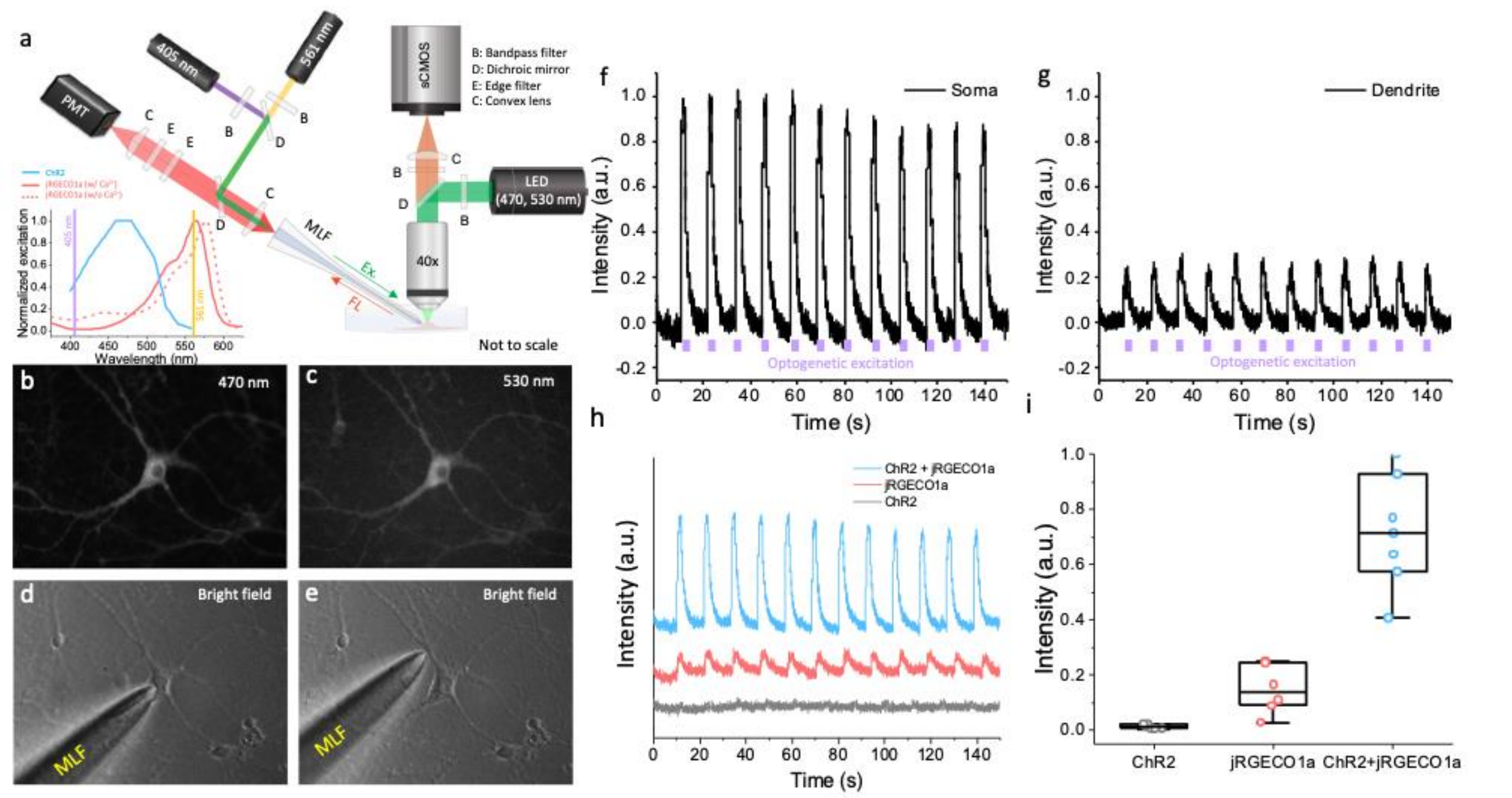

Supplementary Figure 14. Detection of Ca^{2+} transients in hippocampal neural cells with optogenetics. (a) Schematic illustration of the fiber-in/fiber-out all-optical system for ChR2 stimulation and Ca^{2+} recording from the soma and single dendrite using a microlensed fiber (MLF). The inset shows the absorption spectra of ChR2 and jRGECO1a together with the 405 nm and 561 nm excitation laser lines[10,11]. (b,c) Representative fluorescence images of hippocampal neural cells co-expressing ChR2-EYFP and jRGECO1a under LED excitation (470 and 530 nm, respectively). (d,e) Representative bright-field images showing the MLF tip positioned near the soma and dendrite. (f,g) Representative fluorescence enhancement associated with photostimulation-induced Ca^{2+} transients after removal of fiber autofluorescence (405 nm: 20 mW/mm², 9 Hz, 12 pulses, 60 ms duration, 5 s inter-train delay; 561 nm: 3 mW/mm², CW). (h) Representative photomultiplier tube (PMT) traces in cells expressing ChR2 only, jRGECO1a only, or both proteins upon 405 nm stimulation (PMT gain: 10^6; fast Fourier transform low-pass filter, 25 Hz). (i) Box plots indicate the median (center line), interquartile range (IQR) (box), and 1.5 × IQR (whiskers). Individual points represent independent cells (ChR2, n = 6; jRGECO1a, n = 6; ChR2+jRGECO1a, n = 7). Statistical significance was evaluated using one-way ANOVA followed by Tukey's multiple-comparison test (two-sided): ChR2 vs jRGECO1a, P = 0.226; ChR2 vs ChR2+jRGECO1a, P < 0.0001; jRGECO1a vs ChR2+jRGECO1a, P < 0.0001.

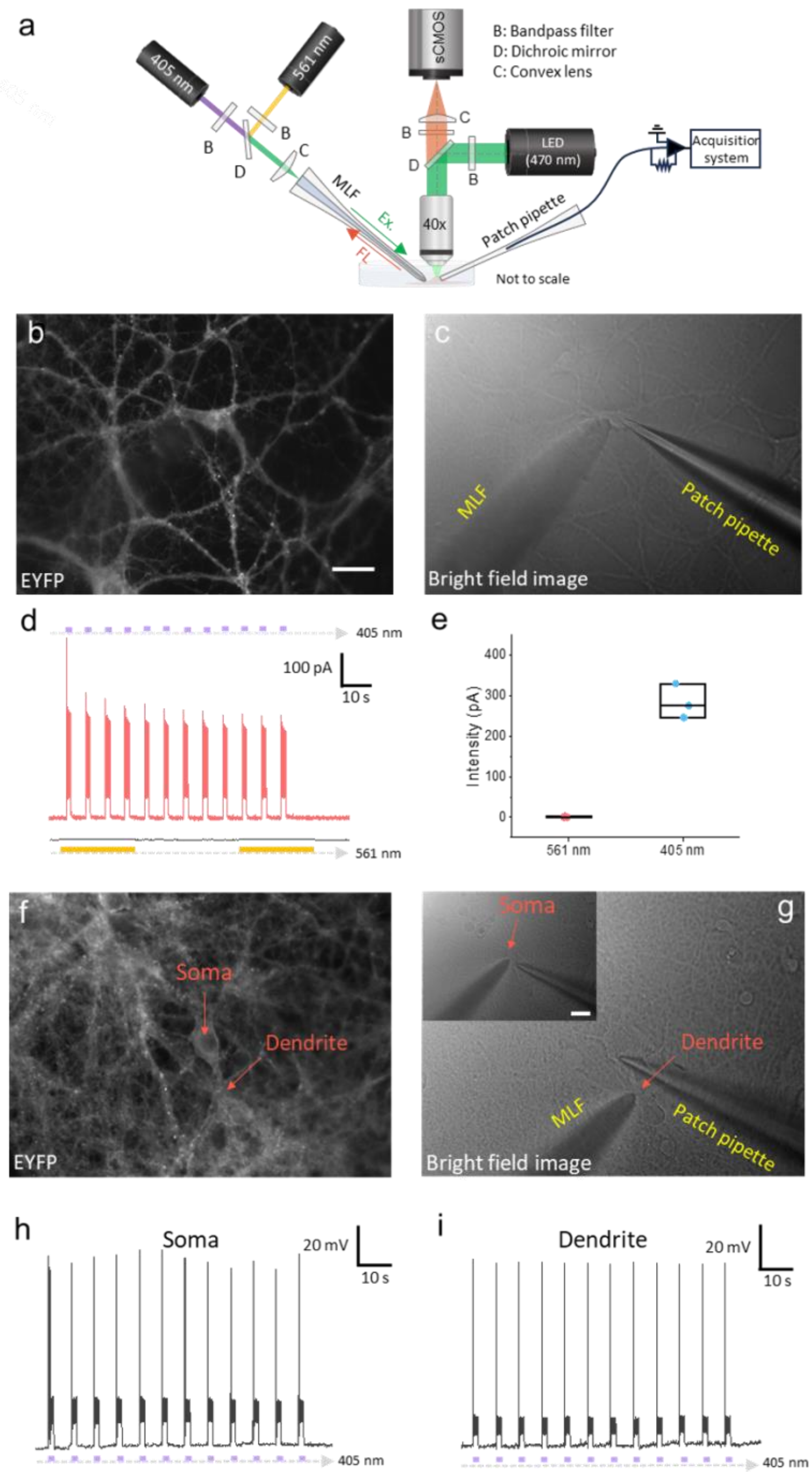

Supplementary Figure 15. Contrasting ChR2 responses to 405 nm and 561 nm laser and their subsequent impact on cellular activity. (a) Schematic representation of the optical and patch clamp setup used for photostimulation and intracellular recording. (b) Fluorescence images of hippocampal neural cells expressing ChR2-EYFP upon illumination with a microscope LED light source (470 nm). (c) Representative bright-field image showing the microlensed fiber (MLF) tip and a glass micropipette electrode near the cell soma. (d,e) Representative intracellular current

responses induced by 405 nm (20 mW/mm², 9 Hz train of 15 pulses, 60 ms duration, 5 s inter-train delay) and 561 nm (3 mW/mm², CW, 30 s), with quantification shown in (e). Box plots indicate median (center line), interquartile range (IQR) (box), and whiskers (1.5 × IQR). Individual points represent independent cells (n = 3 per group). Differences were assessed using a two-sided Mann–Whitney U test (P = 0.0765). (f) Fluorescence images of hippocampal neural cells expressing ChR2-EYFP upon illumination with a microscope LED light source (470 nm). (g) Representative bright-field image of a glass micropipette electrode positioned near the cell soma, while the MLF is positioned either in the dendrite or soma (Inset) for photostimulation. (h,i) Recorded alterations in membrane potential resulting from the photostimulation (20 mW/mm^2, 9 Hz train of 15 pulses with 60 ms duration and 5s delay between the train of the pulses) illuminated at the soma (h) and dendrite (i) sites. FL: Fluorescence

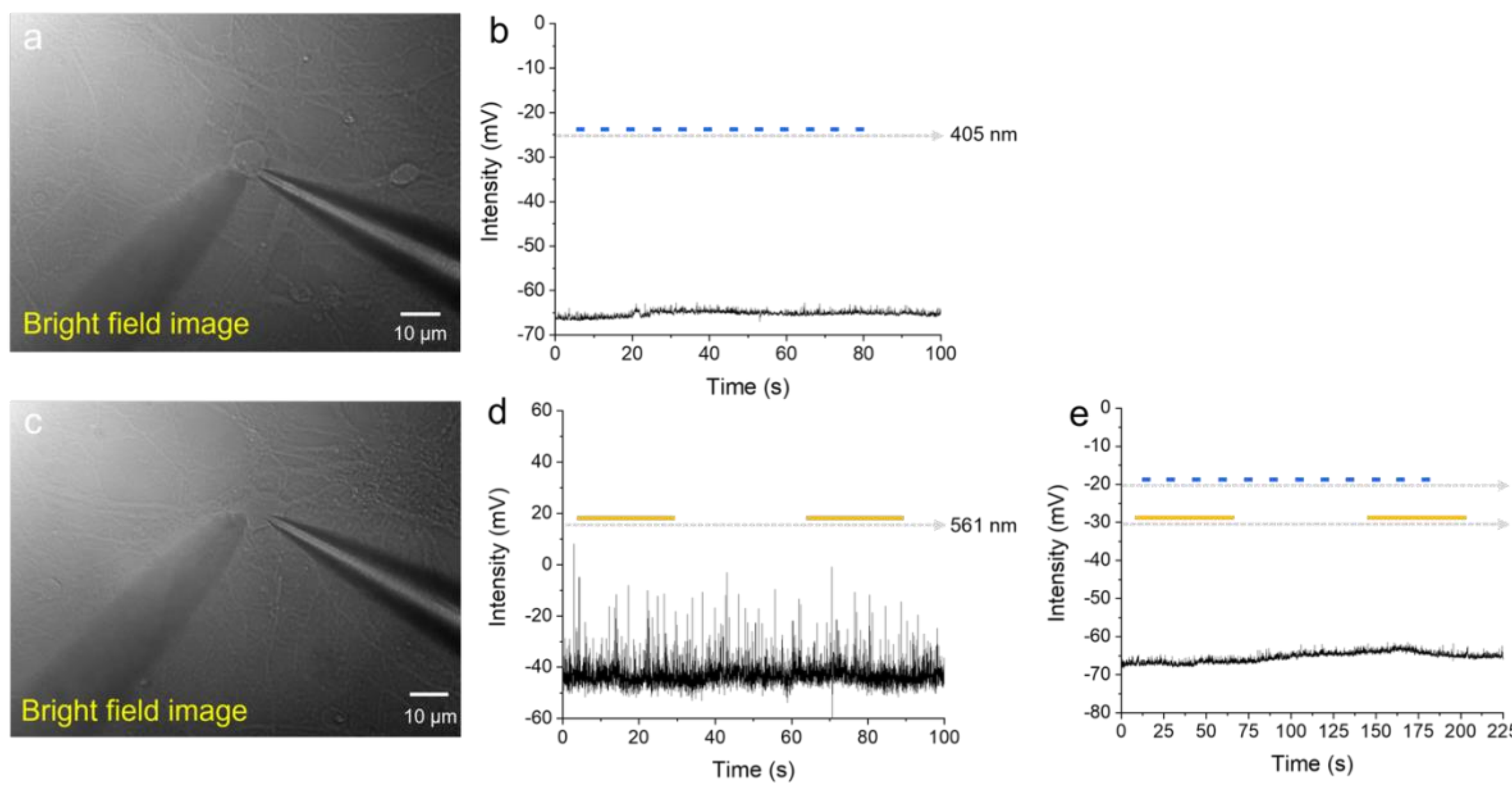

Supplementary Figure 16. Control trials involving a cell expressing solely jRGECO1a and corresponding response comparison (membrane potential) to 405 nm (20 mW/mm^2, 9 Hz train of 15 pulses with 60 ms duration and 5s delay between the train of the pulses), 561 nm (3 mW/mm^2, continuous wave for 30 seconds), and concurrent 405 nm and 561 nm laser stimulation using identical illumination protocols.

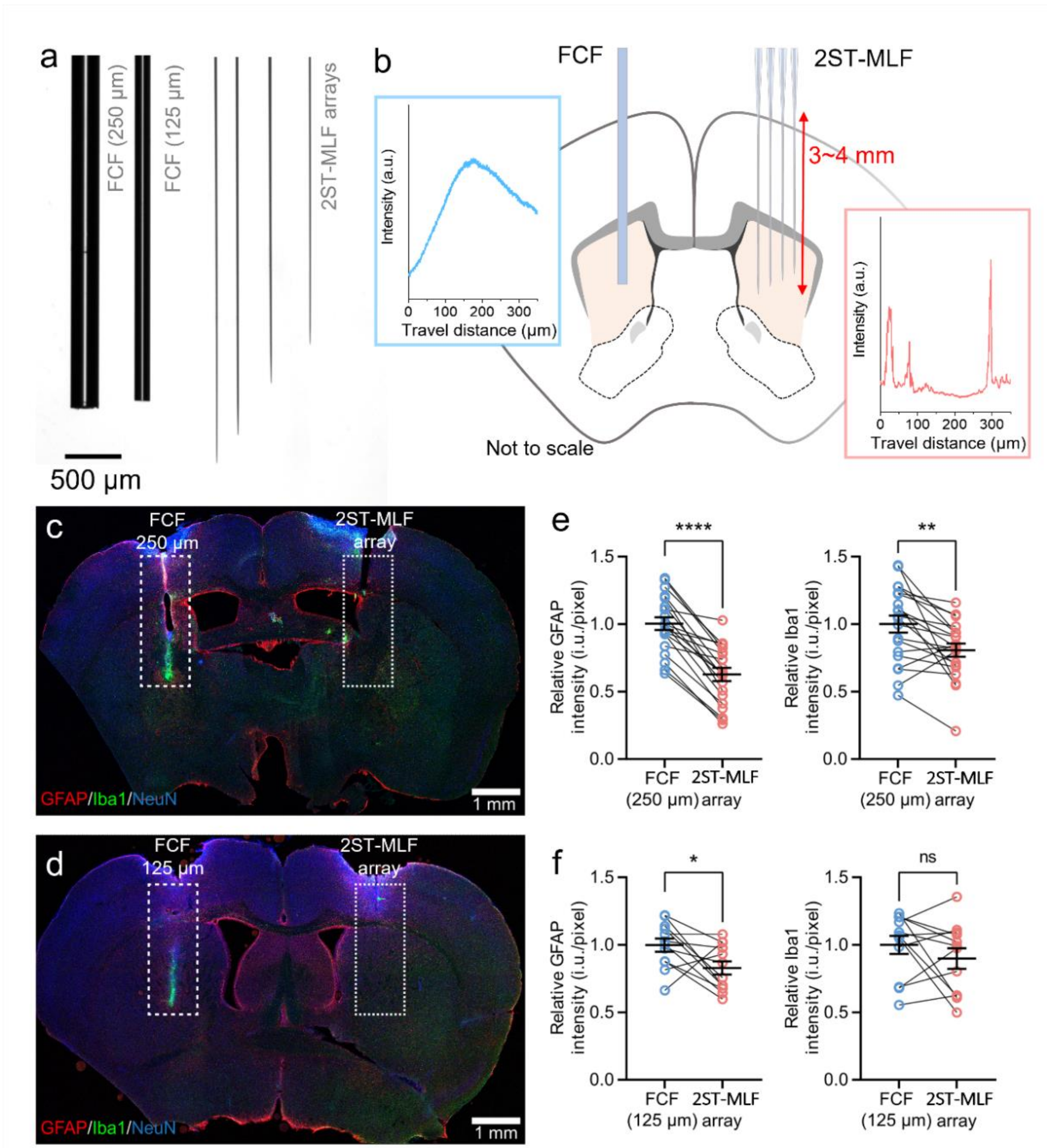

Supplementary Figure 17. Minimal invasiveness of two-step tapered microlensed fibers (2ST-MLF) with high spatial resolution and SNR compared to conventional FCFs (125 and 250 µm). (a) Visual comparison of the three fiber probes, illustrating their relative sizes. (b) Illustration of a single FCF and four 2ST-MLF arrays (tip and shank diameters: 10 µm and 25 µm, respectively) implanted in mouse brains at a depth of 3~4 mm for the invasiveness test. The inset compares jRGECO1a fluorescence captured using a single 125 µm FCF and a single 2ST-MLF (reused from Fig. 7i), highlighting 2ST-MLF's superior spatial resolution and SNR. (c-d) Representative merged fluorescence images of glial fibrillary acidic protein (GFAP), ionized calcium-binding adapter molecule 1 (Iba-1), and neuronal nuclei (NeuN), with dashed lines indicating fiber implantation sites. (e–f) Quantification of GFAP and Iba-1 fluorescence surrounding the

implantation sites. Quantification was performed using 12 regions of interest (ROIs; slices) from 3 mice per group in (e) and 20 ROIs (slices) from 4 mice per group in (f) (FCF and 2ST-MLF). Data are presented as mean ± standard deviation. Differences were assessed using a two-sided paired t-test without adjustment for multiple comparisons. Exact P values: (e), GFAP $P = 0.0383$ and Iba-1 $P = 0.265$; (f), GFAP $P = 1.51 \times 10^{-8}$ and Iba-1 $P = 0.0033$. Significance levels: ns, $P \geq 0.05$; *$P < 0.05$; **$P < 0.01$; ***$P < 0.001$; ****$P < 0.0001$.

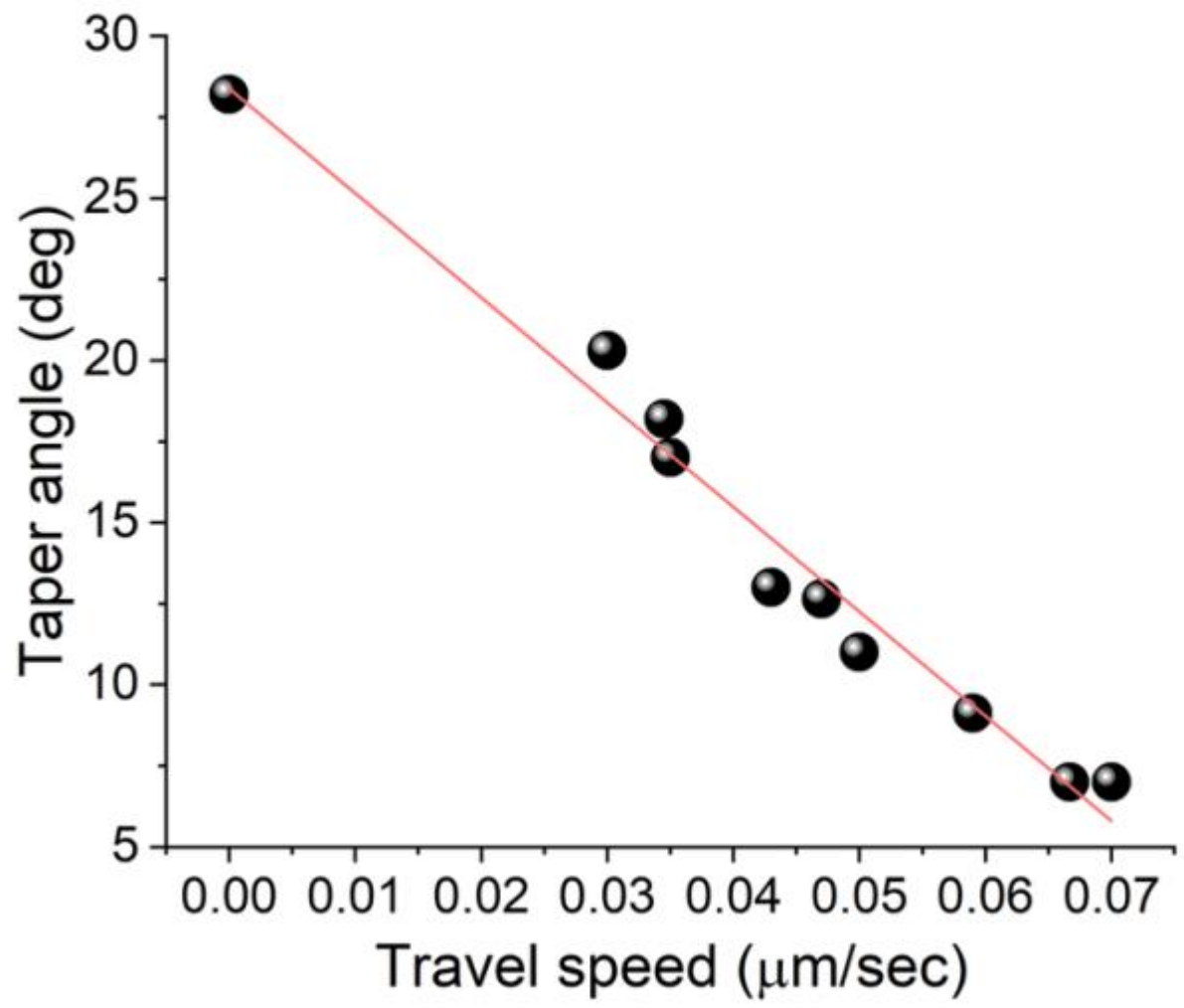

Supplementary Figure 18. Taper angle as a function of the travel speed of the stage motor in 25.9 wt% HF.

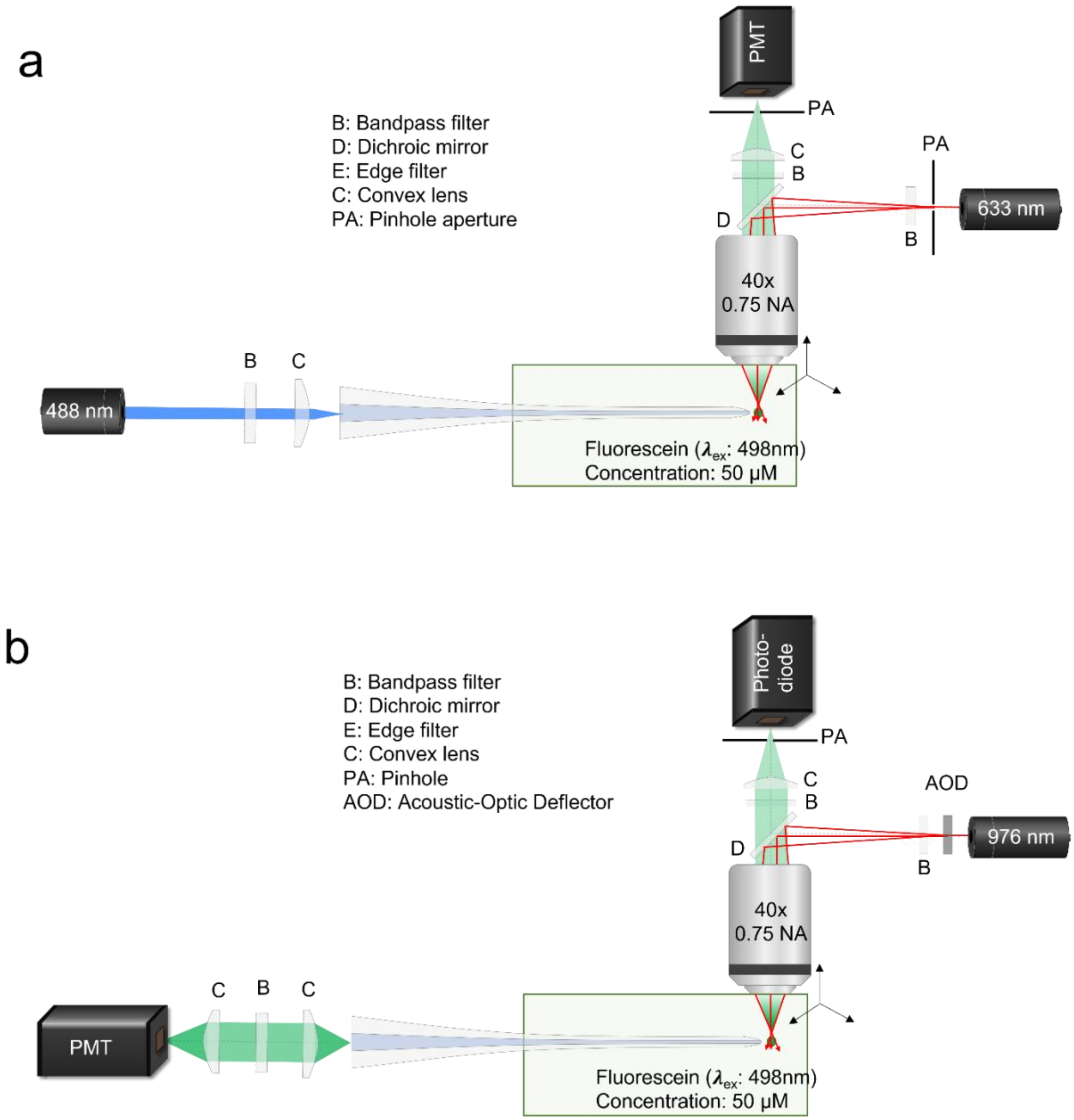

Supplementary Figure 19. Schematic illustration of the optical setup used to analyze the emission (a) and collection (b) profiles of the microlensed fiber (MLF). For the emission profile analysis, a 633 nm laser was employed to capture the fiber geometry, and fluorescence was collected with out-of-focus light minimized by the confocal scanning pinhole for enhanced image resolution. For the collection profile analysis, two-photon scanning microscopy equipped with an acousto-optic deflector was used, with a 500 μs dwell time to excite fluorescence within the focal plane. The emitted fluorescence was then collected through the fiber and detected using a photomultiplier tube (PMT).

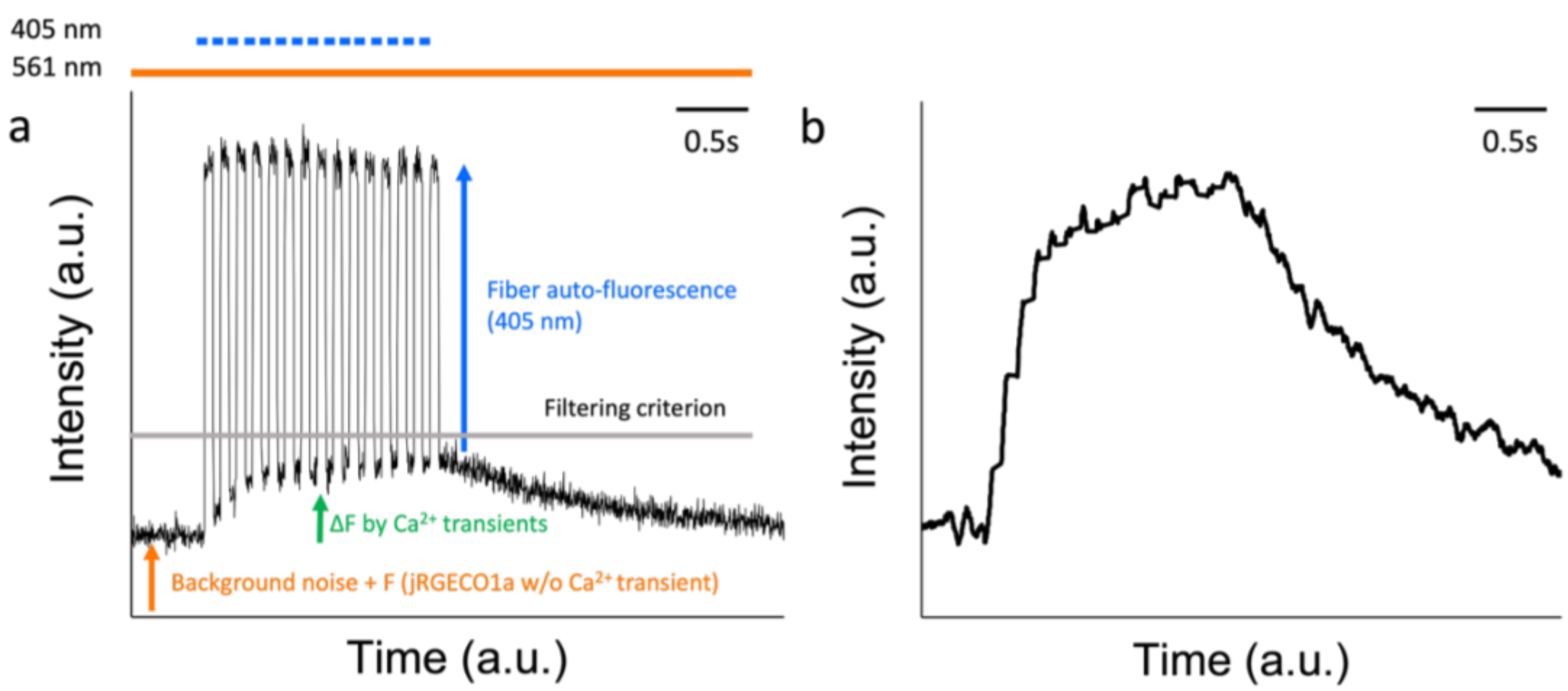

Supplementary Figure 20. Extraction of fluorescence enhancement by Ca^{2+} transients from the fiber auto-fluorescence. (a-b) ChR2-dependent fluorescence vs. time traces for cells expressing ChR2 and jRGECO1a using MLF before (a) and after filtering out the auto-fluorescence generated by 405 nm (9 Hz train of 15 pulses with 60 ms duration) and 561 nm laser illumination via numerical filtering values greater than the filtering criterion and subsequent low pass filter (<25Hz).

Supplementary Table

Supplementary Table 1. Material properties of the etchant used to calculate the local temperature increase induced by the laser emanating from a tapered fiber at $\lambda = 1550$ nm.

Properties	Values
Bulk thermal conductivity $(W/(m \cdot K)$	0.6
Heat capacity at constant pressure $J/(kg \cdot K))$	4184
Ratio of specific heats	1.33
Density (kg/m^3)	997
Refractive index, real part	1.33[12]
Refractive index, imaginary part	0.000098625[12]
Initial temperature (℃)	20

Supplementary References

1. Szabo, V., Ventalon, C., De Sars, V., Bradley, J. & Emiliani, V. Spatially selective holographic photoactivation and functional fluorescence imaging in freely behaving mice with a fiberscope. *Neuron* **84**, 1157–1169 (2014).

2. Wäldchen, S., Lehmann, J., Klein, T., van de Linde, S. & Sauer, M. Light-induced cell damage in live-cell super-resolution microscopy. *Sci. Rep.* **5**, 15348 (2015).

3. Wu, J. *et al.* Improved orange and red $Ca^{2}\pm$ indicators and photophysical considerations for optogenetic applications. *ACS Chem. Neurosci.* **4**, 963–972 (2013).

4. Dalangin, R. *et al.* Far-red fluorescent genetically encoded calcium ion indicators. *bioRxiv* (2020) doi:10.1101/2020.11.12.380089.

5. Guan, N., Liu, Z., Zhang, C. & Jiang, G. Natural convection heat transfer on surfaces of copper micro-wires. *Heat Mass Transf.* **50**, 275–284 (2014).

6. Born, M. & Wolf, E. *Principles of Optics*. (Cambridge University Press, Cambridge, England, 2000).

7. Schmidt, B. S., Yang, A. H., Erickson, D. & Lipson, M. Optofluidic trapping and transport on solid core waveguides within a microfluidic device. *Opt. Express* **15**, 14322–14334 (2007).

8. Xin, H., Xu, R. & Li, B. Optical trapping, driving, and arrangement of particles using a tapered fibre probe. *Sci. Rep.* **2**, 818 (2012).

9. Liu, Z., Guo, C., Yang, J. & Yuan, L. Tapered fiber optical tweezers for microscopic particle trapping: fabrication and application. *Opt. Express* **14**, 12510–12516 (2006).

10. Dana, H. *et al.* Sensitive red protein calcium indicators for imaging neural activity. *Elife* **5**, e12727 (2016).

11. Nagel, G. *et al.* Channelrhodopsin-2, a directly light-gated cation-selective membrane channel. *Proc. Natl. Acad. Sci. U. S. A.* **100**, 13940–13945 (2003).

12. Hale, G. M. & Querry, M. R. Optical constants of water in the 200-nm to 200-µm wavelength region. *Appl. Opt.* **12**, 555–563 (1973).
