# Supplementary material for: Deterministic wet etching of aspheric fiber microlenses with tunable conic geometry for tailored optical functionality
Source: Nat Commun. 2026 Aug 7;17:9569. doi: 10.1038/s41467-026-75331-y (PMC13550405; doi:10.1038/s41467-026-75331-y)
Supplement: Supplementary file 2 [file 41467_2026_75331_MOESM2_ESM.pdf]

Description of Additional Supplementary Files

Supplementary Video 1. Trapping of a borosilicate microbead (7.34 ± 0.87 μm)

A borosilicate microbead is optically trapped using the hyperbolic microlensed fiber (HMLF) with a 980 nm trapping laser (~0.6 mW). The video shows stable trapping of the microbead under laser illumination.

Supplementary Video 2. Axial movement of a borosilicate microbead

A borosilicate microbead is translated along the optical axis while remaining optically trapped by the HMLF, demonstrating axial manipulation.

Supplementary Video 3. Radial movement of a borosilicate microbead

A borosilicate microbead is translated in the radial direction while remaining optically trapped by the HMLF, demonstrating lateral manipulation.

Supplementary Video 4. Release of a borosilicate microbead

The trapped borosilicate microbead is released by switching off the trapping laser, demonstrating controlled particle release.

Supplementary Video 5. Release of a polystyrene (PS) nanoparticle (445 ± 26 nm)

A trapped polystyrene (PS) nanoparticle is released by reducing the power of the 980 nm trapping laser below 3.2 mW, demonstrating controlled optical release of a nanoscale particle.
