# Supplementary material for: Deterministic wet etching of aspheric fiber microlenses with tunable conic geometry for tailored optical functionality
Source: Nat Commun. 2026 Aug 7;17:9569. doi: 10.1038/s41467-026-75331-y (PMC13550405; doi:10.1038/s41467-026-75331-y)
Supplement: Supplementary file 3 [file 41467_2026_75331_MOESM8_ESM.pdf]

nature portfolio

Corresponding author(s): Sanggon Kim

Last updated by author(s): May 21, 2026

Reporting Summary

Nature Portfolio wishes to improve the reproducibility of the work that we publish. This form provides structure for consistency and transparency in reporting. For further information on Nature Portfolio policies, see our Editorial Policies and the Editorial Policy Checklist.

Statistics

For all statistical analyses, confirm that the following items are present in the figure legend, table legend, main text, or Methods section.

n/a	Confirmed	
☐	☒	The exact sample size (n) for each experimental group/condition, given as a discrete number and unit of measurement
☐	☒	A statement on whether measurements were taken from distinct samples or whether the same sample was measured repeatedly
☐	☒	The statistical test(s) used AND whether they are one- or two-sided<br>*Only common tests should be described solely by name; describe more complex techniques in the Methods section.*
☒	☐	A description of all covariates tested
☒	☐	A description of any assumptions or corrections, such as tests of normality and adjustment for multiple comparisons
☐	☒	A full description of the statistical parameters including central tendency (e.g. means) or other basic estimates (e.g. regression coefficient) AND variation (e.g. standard deviation) or associated estimates of uncertainty (e.g. confidence intervals)
☐	☒	For null hypothesis testing, the test statistic (e.g. F, t, r) with confidence intervals, effect sizes, degrees of freedom and P value noted<br>*Give P values as exact values whenever suitable.*
☒	☐	For Bayesian analysis, information on the choice of priors and Markov chain Monte Carlo settings
☒	☐	For hierarchical and complex designs, identification of the appropriate level for tests and full reporting of outcomes
☒	☐	Estimates of effect sizes (e.g. Cohen's d, Pearson's r), indicating how they were calculated

Our web collection on statistics for biologists contains articles on many of the points above.

Software and code

Policy information about availability of computer code

Data collection	PMT signals were acquired using Spike2 software (Cambridge Electronic Design). Optical and thermal simulation data used for modeling were generated using COMSOL Multiphysics (version 6.2). No custom code was used for data collection.
Data analysis	Data analysis was performed using MATLAB(R2022a), ImageJ, and Origin (2022). Custom MATLAB code was used to process COMSOL-exported geometry and temperature profiles, calculate etch-rate distributions, simulate tapered-fiber geometry evolution, and perform taper-geometry analyses. ImageJ was used for image processing and fluorescence quantification, and Origin was used for data visualization and graph preparation.

For manuscripts utilizing custom algorithms or software that are central to the research but not yet described in published literature, software must be made available to editors and reviewers. We strongly encourage code deposition in a community repository (e.g. GitHub). See the Nature Portfolio guidelines for submitting code & software for further information.

Data

Policy information about availability of data

All manuscripts must include a data availability statement. This statement should provide the following information, where applicable:

- Accession codes, unique identifiers, or web links for publicly available datasets
- A description of any restrictions on data availability
- For clinical datasets or third party data, please ensure that the statement adheres to our policy

Data supporting the findings of this study are provided in the main article, Supplementary Information, and Source Data files. Data necessary to interpret, verify, and

reproduce the reported findings are available through these materials. MATLAB code implementing the physics-separated multiphysics framework and the geometry-agnostic axial rounding metric is publicly available through the repository referenced in the Code Availability statement.

Research involving human participants, their data, or biological material

Policy information about studies with human participants or human data. See also policy information about sex, gender (identity/presentation), and sexual orientation and race, ethnicity and racism.

Reporting on sex and gender	Not applicable.
Reporting on race, ethnicity, or other socially relevant groupings	Not applicable.
Population characteristics	Not applicable.
Recruitment	Not applicable.
Ethics oversight	Not applicable.

Note that full information on the approval of the study protocol must also be provided in the manuscript.

Field-specific reporting

Please select the one below that is the best fit for your research. If you are not sure, read the appropriate sections before making your selection.

[x] Life sciences [ ] Behavioural & social sciences [ ] Ecological, evolutionary & environmental sciences

For a reference copy of the document with all sections, see nature.com/documents/nr-reporting-summary-flat.pdf

Life sciences study design

All studies must disclose on these points even when the disclosure is negative.

Sample size	No statistical methods were used to predetermine sample size. For biological experiments, sample sizes were selected based on experimental feasibility and the objective of evaluating optical probe performance under relevant biological conditions and were considered appropriate to evaluate fluorescence and neural recording performance. Comparative experiments between the microlensed fiber (MLF) and flat-cleaved fiber (FCF) were designed using paired measurements on the same biological preparation whenever possible (e.g., cultured-cell experiments and unilateral implantation comparisons) to reduce sample-dependent variability. Experiments intended to demonstrate MLF performance only are presented as representative measurements.
Data exclusions	No biological data were excluded from analyses except in predefined cases of technical failure that prevented reliable measurement. For intracellular potential recordings, only cells exhibiting substantial damage induced during pipette insertion were excluded. The same exclusion criterion was applied consistently across related intracellular recording experiments. No additional exclusions were applied.
Replication	Biological experiments were performed using independent biological samples where applicable. Comparative experiments between MLF and FCF were conducted using paired measurements from the same biological preparation whenever possible to reduce biological variability. Measurements requiring independent recordings were performed using independent cells or animals where appropriate. Replication information for each experiment is provided in the corresponding figure legends and Methods section.
Randomization	Randomization was not applied because the study focused primarily on technology development, performance evaluation, and validation rather than comparison of treatment groups. Biological samples were selected based on predefined experimental suitability criteria.
Blinding	Blinding was not applied because the study focused on technology validation and objective fluorescence and electrophysiological measurements rather than subjective outcome assessment.

Reporting for specific materials, systems and methods

We require information from authors about some types of materials, experimental systems and methods used in many studies. Here, indicate whether each material, system or method listed is relevant to your study. If you are not sure if a list item applies to your research, read the appropriate section before selecting a response.

Materials & experimental systems

n/a	Involved in the study	
☐	☒	Antibodies
☒	☐	Eukaryotic cell lines
☒	☐	Palaeontology and archaeology
☐	☒	Animals and other organisms
☒	☐	Clinical data
☒	☐	Dual use research of concern
☒	☐	Plants

Methods

n/a	Involved in the study	
☒	☐	ChIP-seq
☒	☐	Flow cytometry
☒	☐	MRI-based neuroimaging

Antibodies

Antibodies used

Primary antibodies:
• Chicken polyclonal anti-GFAP (glial fibrillary acidic protein), Abcam, catalogue no. ab4674, dilution 1:2000.
• Rabbit polyclonal anti-Iba1, Wako, catalogue no. 019-19741, dilution 1:2000.
• Mouse monoclonal anti-NeuN, Millipore, catalogue no. MAB377, dilution 1:1000.

Secondary antibodies:
• AlexaFluor™ 647-conjugated goat anti-chicken, Invitrogen, catalogue no. A21449, dilution 1:500.
• AlexaFluor™ 488-conjugated goat anti-rabbit, Invitrogen, catalogue no. A11008, dilution 1:500.
• AlexaFluor™ 405-conjugated goat anti-mouse, Invitrogen, catalogue no. A31553, dilution 1:500.

Lot numbers were not recorded.

Validation

All antibodies were selected based on prior validation by the manufacturers for immunofluorescence applications and established use in mouse brain tissue. Primary antibodies have been extensively used in more than 5000 peer reviewed studies, including previous studies from our lab. In this study, immunostaining was used to evaluate relative differences in tissue response between FCF and 2ST-MLF implantation rather than absolute quantification of biological markers. Comparative analysis was performed using paired implantation within the same animal and identical staining, imaging, and fluorescence acquisition conditions across groups to minimize inter-animal and experimental variability.

Animals and other research organisms

Policy information about studies involving animals; ARRIVE guidelines recommended for reporting animal research, and Sex and Gender in Research

Laboratory animals

Adult male C57BL/6J mice (Jackson Laboratory, RRID: IMSR_JAX:000664) were used for in vivo experiments. Male Gad2-IRES-Cre mice (Gad2tm2(cre)Zjh/J; RRID: IMSR_JAX:010802) were crossed with female Ai32(RCL-ChR2(H134R)/EYFP) mice (RRID: IMSR_JAX:024109) to generate Gad2-Cre×Rosa-CAG-ChR2 mice for ex vivo brain-slice experiments. Animal ages are specified in the corresponding experimental sections. Animals were housed on a 12 h light/dark cycle with ad libitum access to food and water at 21 ± 1 °C and 40–45% relative humidity.

Wild animals

This study did not involve wild animals.

Reporting on sex

Only male mice were used in this study. The study was not designed to investigate sex-specific differences, and no sex-based analyses were performed.

Field-collected samples

This study did not involve field-collected samples.

Ethics oversight

All animal procedures were approved by the Committee for Animal Protection of Université Laval (CPAUL) and conducted in accordance with the guidelines of the Canadian Council on Animal Care.

Note that full information on the approval of the study protocol must also be provided in the manuscript.

Plants

Seed stocks	Not applicable.
Novel plant genotypes	Not applicable.
Authentication	Not applicable.
