# Supplementary material for: Deterministic wet etching of aspheric fiber microlenses with tunable conic geometry for tailored optical functionality
Source: Nat Commun. 2026 Aug 7;17:9569. doi: 10.1038/s41467-026-75331-y (PMC13550405; doi:10.1038/s41467-026-75331-y)
Supplement: Supplementary file 4 [file 41467_2026_75331_MOESM9_ESM.pdf]

nature portfolio

Peer Review File

Deterministic Wet Etching of Aspheric Fiber Microlenses with Tunable Conic Geometry for Tailored Optical Functionality

Corresponding Author: Dr Sanggon Kim

This file contains all reviewer reports in order by version, followed by all author rebuttals in order by version.

Version 0:

Reviewer comments:

Reviewer #1

(Remarks to the Author)
This work demonstrates the potential application of tapered optical fibers as neural probes in vivo. However, the main focus of the paper, i.e., the fabrication of strictly parabolic fiber tips via controlled chemical etching, lacks rigorous and systematic validation. The fundamental principles and logical reasoning are flawed, with significant gaps in the technical justification and questionable transitions between arguments. Moreover, the text repeatedly overstates the paper's contributions. Overall, the quality falls below the standards of Nature Communications.

Details of my comments please find in the attachment.

Reviewer #2

(Remarks to the Author)
This manuscript by Kim et al. presents a significant advancement in fiber optic technology: Laser-Controlled Wet-Chemical Etching (LCWCE) for fabricating parabolic micro-lensed fibers (PMLFs). The method achieves unprecedented sub-micron control over lens shape and working distance, overcoming long-standing limitations of conventional techniques in precision, reproducibility, and scalability. The authors thoroughly demonstrate the superiority of their PMLFs in two demanding applications. First, they achieve record-low power optical trapping of nanoparticles and microparticles, highlighting highly efficient focusing. Second, they demonstrate groundbreaking in vivo neural interfacing, enabling non-invasive, all-optical single-cell recording and modulation at depths >1.5 mm with exceptional spatial resolution and signal-to-noise ratio. The technique's versatility across fiber types, low cost, and batch-fabrication potential make it highly impactful. This work effectively bridges a critical fabrication gap and establishes a transformative platform for biophotonics, neuroscience, and precision optical technologies. Several suggestions are supplied:
1. Suggest the authors consider explicitly mentioning the key applications (optical trapping, neural recording) and the achieved performance metrics (e.g., trapping power, spatial resolution) in the abstract to immediately highlight the practical impact.
2. Suggest the authors enhance specify the wavelength at which the "beamwidth of 24.4 μm" was measured for consistency with other beamwidth reports.
3. Suggest the authors enhance the part about more detail on how the initial taper angle quantitatively influences the resulting temperature profile and, consequently, the final lens curvature.
4. Suggest the authors enhance present quantitative data on the reproducibility of the parabolic lens shape across multiple batches.
5. Suggest the auhtors enhance the part about tests performed to assess the long-term durability of these lenses.
6. Suggest the authors enhance the part about the experimentally measured beam profile and trapping efficiency compare to simulations of an ideal parabolic lens of the same dimensions.
7. To further solidify this, did you perform post-hoc histology to confirm the physical location of the fiber tip relative to the recorded neuron(s) and verify that the recorded signals indeed originated from a single, identified cell body rather than a small, unresolved cluster?

Reviewer #3

(Remarks to the Author)

I co-reviewed this manuscript with one of the reviewers who provided the listed reports. This is part of the Nature Communications initiative to facilitate training in peer review and to provide appropriate recognition for Early Career Researchers who co-review manuscripts.

Reviewer #4

(Remarks to the Author)
The submitted manuscript proposes a fabrication method for microlensed fibers based on laser-controlled wet chemical etching (LCWCE). The study introduces a potentially useful approach that may be of interest to researchers working on optical fiber microstructuring and related applications. However, while the method could have value in specialized contexts, the manuscript in its current form does not meet the standards of broad conceptual impact and presentation quality expected for Nature Communications. The work would benefit from substantial revision to enhance the accuracy, clarity, and precision of both the language and the technical explanations, as well as from a more comprehensive contextualization within the existing literature. After these improvements, the paper may be more appropriate for publication in a specialized optics or photonics journal. Please find my detailed comments below:

•Abstract: it is not clear what "longitudinal" exactly means at this early point in the manuscript. I suggest the authors reword to better convey the intended concept

•Two-photon lithography (TPL), which is highly relevant in this context, is only indirectly addressed through a few application-focused references (e.g., 10 and 14) but is not explicitly mentioned among the fabrication methods for microlensed fibers (MLFs) in the Introduction (page 2). It should be included there for completeness and fairness in presenting the state of the art, especially since TPL is a direct competing technique that offers a much greater degree of freedom in controlling the geometrical shape compared to the other fabrication methods discussed.

•pag 4: “This approach utilizes the light emitted from the fiber to locally heat the etchant, influencing its absorption and increasing the reaction rate […]”. “Absorption" of what? Like this it seems that you mean that light is absorbed, and then heats the etchant, and that in turns changes the absorption (so this supposedly has a temperature dependence)

•pag 4: “For instance, when the flat fiber end is immersed in the etchant and light with a high absorption coefficient is emitted from it, […]”. Please rephrase as light does not have “a high absorption coefficient“

•pag 4: “This gradient causes a hole to form within the optical cores, further degrading the fibers focusing and collection efficiency.” At this stage, the fiber still has a flat end, so it is not clear what “focusing and collection efficiency” the authors are referring to

•pag 4: “[…] follows a higher-order decay than linear.” this sentence needs more precision in language. What’s a “higher-order decay’?

•pag 4: “A chemically tapered initial structure offers two key advantages”. “Advantages” vs other methods, or what? This is not clear

•pag 4: what the exact mechanism for light absorption here? At this point in the manuscript, what absorbs and at what wavelength are not clear. Some of these info seems to be only provided in figure 1 caption (also: what laser power for that temperature map in Fig. 1c?) and later in the manuscript

•pag 4: “gradual transformation […] structures.” Is there any opto/therm/fluido-dynamic modeling that enables the description of this?

•pag 5: “This equation describes the relative increase in the etching rate compared to the etching rate at room temperature (20°C) under varying incident power levels.” Rephrase for the fact that equ (3) does not directly integrate laser power as a parameter

•pag 5: “A steeper gradient slope, resulting from the exponential dependence on temperature (Fig. 1e), proves its effectiveness in forming rounded structures.” The steeper gradient can be leveraged to get those structures, but cannot “prove its effectiveness”

•pag 5: “After the LCWCE process, the sharp tip of the tapered fiber undergoes etching, yielding a smoothly rounded fiber end.” This description is not clear enough at this point in the manuscript, and needs rephrasing. I seem to interpret that the etching process proceeds up to a certain point with the 1550nm laser on (”LCWCE process”), and then at some point the laser is tuned off and the etching continues without its influence? Is that correct?

•pag 5: “effectively confining the incident light at the fiber tip (Figs. 2c,d).”. “Confining the incident light” is not what I see in Fig2. I only see a focusing output light from the etched fiber (in fig 2d)

•pag 5: “[…] marking the first experimental demonstration of subwavelength beam confinement (beamwidth: 406 nm) of lensed fiber in water.” What do the authors mean with “first experimental demonstration”? “First” with respect to what? The authors should provide more context for that claim

•pag 5: in the discussion about the evolution of the etching process with consequent different shaping of the fiber tip, the used/needed optical power is only mentioned at the end of the section, while it would be better to say it before

•pag 5-6: “Here, b was fixed at 5 µm, corresponding to a single-mode fiber with a mode field diameter of 10 µm (@ 1500 nm).” It is not clear to me why this 5 um should correspond to a SM fiber with a 10um MFD

•pag 6: “As etching proceeds, tip curvature decreases with time, […]”. Is this the etching process that happens AFTER turning the 1550nm laser off? It is currently not clearly described

•pag 7: “PMLFs fabricated via our all-wet laser-controlled etching process preserve a uniform optical core diameter along the fiber length.“. The proposed process is tapering the fiber to sizes well below the fiber core’s diameter (see Fig. 2), so this statement is hard to interpret at this point.

•Page 7: Some of the claims appear to be overstated. For example, although the proposed method may offer improved shape control compared to certain existing techniques, it cannot be considered the most accurate overall. In contrast, two-photon lithography (TPL) enables full control over all geometrical parameters of a parabolic profile ($y = ax^2 + bx + c$). The authors should clarify which specific parameters their method can actually control and to what degree.

•pag 9: “The micro-bead was suspended in agar gel, then solidified and cut perpendicular to the probe axis.” This sentence seems to mean that the bead was “solidified” and ”cut”. Is that what the authors wanted to mean?

•The reported application of fiber-based Optical Tweezers (OT) as a benchmark to demonstrate the significance of the proposed method is not particularly compelling. Fiber OT systems with the geometry presented by the authors inherently feature very short working distances in order to achieve the high numerical aperture required for full 3D optical trapping. Consequently, their applicability is intrinsically limited, as truly contactless trapping can typically be realized only for submicron-sized particles (< 1 µm in diameter). In fact, in Fig. S8, the 7 µm bead shown could well be in physical contact with the fiber tip rather than optically trapped in a contactless configuration.

•Page 13: Most of the discussion section mainly reiterates points already presented earlier in the manuscript, with redundant repetitions and little critical analysis. A substantive discussion is missing — one that addresses the current limitations of the proposed method and outlines possible directions for future improvement. For instance, the authors should comment on the reproducibility of the fabrication process and discuss whether, and how, the fabrication parameters (e.g., laser power, etching time, etc.) need to be adjusted depending on the desired final tip geometry.

•Figure 2:
- the LITWCE label should be replaced with LCWCE.
- does TA stand for “taper angle” in subfigure 2e? If yes, please specify if this the angle at the end of the LCWCE step (is my understanding correct?) and add it to figure/caption.

Version 1:

Reviewer comments:

Reviewer #1

(Remarks to the Author)
I have reviewed the revised version of this manuscript, and also carefully read the responses to other reviewers' comment. I appreciate the efforts made by the authors to fully address the comments from all reviewers, and acknowledge that the paper has been thoroughly revised.

While the study offers a technical improvement, I find that its scientific novelty does not meet the bar expected for publication in Nature Communications. The final decision regarding acceptance is at the discretion of the editorial office.

A few comments for the authors to further improve the manuscript:
1. The quality of the figures is relatively low, and the presentation of the content feels somewhat unfocused. I recommend that the authors further optimize the figures to improve clarity and readability, and reorganize the text to present the results and their significance in a more cohesive manner.
2. For the neural probing, some recent literatures for high-precision optical neural modulation (single cell or even subcellular precision) were missing, for example Light:Sci. Appl. 13, 258 (2024).

(Remarks on code availability)
No access to the code.

Reviewer #2

(Remarks to the Author)
The authors improved the draft with the comments from the reviewers, so now i can recommend it publish on Nature Communications

(Remarks on code availability)

Reviewer #3

(Remarks to the Author)
I co-reviewed this manuscript with one of the reviewers who provided the listed reports. This is part of the Nature Communications initiative to facilitate training in peer review and to provide appropriate recognition for Early Career Researchers who co-review manuscripts.

(Remarks on code availability)

Reviewer #4

(Remarks to the Author)
The authors have improved the manuscript in several respects compared with the previous version. In particular, the addition of simulations to provide a more explicit theoretical background for the proposed mechanism is a useful step toward better supporting the physical interpretation. The revised version is also more developed in parts of the presentation and contextualization.
However, my main concerns remain substantially unchanged. In my view, the work still does not reach the level of breadth, general interest, and conceptual impact expected for Nature Communications. The proposed method may be potentially useful and of interest to researchers working on optical fiber microstructuring and related applications, but the overall scope and significance still appear more suitable for a specialized optics or photonics journal rather than for a broad multidisciplinary venue.
In addition, although some sections have been revised, the manuscript still suffers at places from expressions unnecessarily repeated even within newly added text (e.g. "(10.9 wt%) and a laser power of 150 mW" is repeated at least twice), and the writing is not always as accurate and rigorous as it should be.
I also note that some comments from other reviewers, especially Reviewer #1, do not appear to have been fully resolved in a sufficiently convincing way.
Therefore, while I acknowledge the efforts made by the authors to improve the manuscript, I maintain my recommendation that the paper is not suitable for publication in Nature Communications.

(Remarks on code availability)

Open Access This Peer Review File is licensed under a Creative Commons Attribution 4.0 International License, which permits use, sharing, adaptation, distribution and reproduction in any medium or format, as long as you give appropriate credit to the original author(s) and the source, provide a link to the Creative Commons license, and indicate if changes were made.
In cases where reviewers are anonymous, credit should be given to 'Anonymous Referee' and the source.

The images or other third party material in this Peer Review File are included in the article's Creative Commons license, unless indicated otherwise in a credit line to the material. If material is not included in the article's Creative Commons license and your intended use is not permitted by statutory regulation or exceeds the permitted use, you will need to obtain permission directly from the copyright holder.
To view a copy of this license, visit https://creativecommons.org/licenses/by/4.0/

Response to Reviewers

Reviewer 1.

Response to the Reviewer's Overall Statement

Reviewer's Overall Statement: "This work demonstrates the potential application of tapered optical fibers as neural probes *in vivo*. However, the main focus of the paper, i.e., the fabrication of strictly parabolic fiber tips via controlled chemical etching, lacks rigorous and systematic validation. The fundamental principles and logical reasoning are flawed, with significant gaps in the technical justification and questionable transitions between arguments. Moreover, the text repeatedly overstates the paper's contributions. Overall, the quality falls below the standards of Nature Communications."

Author's response to the Overall Statement:
We thank the reviewer for this critical assessment. In response, we have substantially revised the manuscript to directly address all concerns regarding rigor, validation, logical consistency, and scope. The revised manuscript differs fundamentally from the original submission in both technical depth and evidentiary support.

(1) Rigorous and systematic validation of LCWCE
The revised manuscript no longer relies solely on experimental demonstration or simplified simulations to validate laser-controlled wet-chemical etching (LCWCE). We have introduced a physics-separated multiphysics framework that links wave-optics electromagnetic power deposition, steady-state heat transfer, and temperature-dependent wet-etch kinetics. This framework provides first-principles validation of how localized laser heating generates an axial etching-rate gradient that deterministically sculpts the fiber tip geometry. The numerical model captures the experimentally observed evolution of the fiber apex and directly validates the underlying etching mechanism (new Fig. 2, 3, and Supplementary Information).

Importantly, to the best of our knowledge, there are no prior studies that combine wave-optics electromagnetic simulations, microscale heat transfer, and temperature-dependent wet-chemical reaction kinetics to predict geometry evolution in fiber-based etching systems. Existing multiphysics studies typically have been limited to coupling optical fields to thermal responses or thermal fields to chemical kinetics in isolation, but do not integrate the full physics from optical power deposition to temperature-driven surface evolution due to the different required spatial and temporal resolution scales over the exceeding process time. The framework introduced here, therefore, addresses a well-recognized gap by providing a computationally tractable yet physically grounded strategy for validating LCWCE without resorting to prohibitively expensive fully coupled transient simulations.

(2) Clarification of physical principles and logical structure
We have restructured the manuscript to clearly separate (i) physical mechanism, (ii) numerical validation, and (iii) experimental realization. Transitions between sections have been rewritten to explicitly connect optical heating, microscale heat transport, and reaction kinetics to the resulting geometry evolution. Importantly, we clarify that LCWCE does not simply "round" fiber tips, but

enables controlled transitions across the full aspheric conic family governed by a spatially defined etching-rate and initial taper geometry. This revision resolves the logical gaps identified by the reviewer and establishes a clear causal chain from optical excitation to final geometry.

(3) Robust geometric verification beyond "parabolic-only" claims
We agree that the original framing placed undue emphasis on strictly parabolic geometries. In the revised manuscript, we present a systematic conic analysis based on extracted tip profiles, conic fitting, and goodness-of-fit metrics. This analysis demonstrates that LCWCE enables deterministic access to hyperbolic, parabolic, prolate elliptical geometries, rather than a single idealized shape (new Figs. 1 and 4; Supplementary Information). The consistently high fit quality across multiple tips and etching conditions provides rigorous and quantitative geometric validation.

(4) Reproducibility and robustness
To address concerns regarding reproducibility, we added a quantitative analysis of independently fabricated fiber tips prepared under identical conditions (Fig. S2). The resulting apex radius variation (1.49 ± 0.24 μm, n = 6) confirms high fabrication consistency without reliance on specialized micromachining tools.

(5) Scope refinement and moderation of claims
We have carefully revised the text to remove overstatements and sharpen the scope of the contributions. The revised manuscript emphasizes that LCWCE provides deterministic, scalable control over fiber-tip geometry rather than claiming exclusive realization of a single "ideal" parabolic lens. Application demonstrations in optical trapping and *in vivo* neural interrogation are now explicitly framed as enabled by optimized geometries validated by numerical modeling, not as standalone claims.

In summary, the revised manuscript now provides:

- A physics-based numerical validation of the LCWCE mechanism,
- Systematic geometric analysis across the full aspheric conic family,
- Quantitative reproducibility assessment, and
- A more precise and restrained framing of the work's significance.

We respectfully submit that these additions directly address the reviewer's concerns and elevate the manuscript to the level of rigor, clarity, and technical justification expected for **Nature Communications**.

Response to the Reviewer's Detailed Statement

Reviewer comment 1: What improvement does this method offer compared to other tapered optical fiber fabrication, such as thermal pulling and breaking? Based on the results presented, there appears to be no substantial advancement over existing techniques.

Author's response:
We thank the reviewer for this important question. Owing to space limitations, the original manuscript did not include a detailed comparison of LCWCE with existing fabrication approaches.

Below, we explicitly clarify the practical and technical limitations of commonly used methods and the specific advances enabled by LCWCE.

(1) Comparison with thermal pulling and breaking (including arc-based methods)
Thermal pulling and breaking are widely used because of their simplicity and accessibility; however, they face several intrinsic limitations that constrain their utility for predictable, application-specific micro-lensed fiber fabrication:

(2) Intrinsic geometric constraints: In practice, thermal pulling and arc-based fabrication methods rely on high-temperature melting followed by surface-tension–driven reflow, which inherently biases the fiber tip toward spherically rounded geometries. Formation of non-spherical or aspheric profiles such as hyperbolic, parabolic, or prolate elliptical shapes typically requires abrupt or uncontrolled events (e.g., partial tip breakage or collapse), introducing substantial variability and yielding unpredictable final geometries. While we do not claim that such conic shapes are fundamentally unattainable using these approaches, there is, to our knowledge, no prior work demonstrating predictable and controllable access to a broad range of conic geometries supported by high-resolution structural characterization and quantitative conic fitting. In particular, previous studies have not reported systematic extraction of conic constants with sufficient fitting accuracy to substantiate deterministic control across the conic family, as demonstrated in the revised manuscript.

(3) Lack of independent parameter control:
In thermal pulling-based approaches, tip curvature, final diameter, taper length, taper angle, and fiber type are strongly interdependent rather than independently tunable. This coupling makes application-specific optimization difficult or infeasible. For example, as demonstrated in our manuscript, the multi-step tapered fiber geometries required for optimized biosensing and neural applications cannot be achieved using conventional methods, owing to the strong and unavoidable coupling between taper length, taper angle, tip curvature, and fiber diameter.

(4) Limited reproducibility across systems and laboratories:
The final geometry is highly sensitive to filament temperature, heating uniformity, pulling speed, and fiber alignment, all of which vary substantially across setups. Minor variations in equipment configuration or filament condition typically require full re-optimization, resulting in poor reproducibility across laboratories. Although detailed protocols exist in some literature, these methods are inherently system-dependent and therefore difficult to reproduce reliably in other labs.

(5) Comparison with focused ion beam (FIB) milling
We fully acknowledge that focused ion beam milling provides outstanding geometric precision. However, FIB milling is inherently costly (often several hundred dollars per probe), slow, and dependent on specialized facilities. These constraints limit accessibility, scalability, and routine adoption, particularly for applications requiring batch fabrication, rapid iteration, or compatibility with diverse fiber types and geometries.

(6) Improvement offered by LCWCE
LCWCE fundamentally differs from the above approaches by providing *direct and deterministic control* over the axial etching-rate profile through localized laser heating. This enables continuous

and reproducible access to a broad family of aspheric conic geometries, including hyperbolic, parabolic, prolate elliptical profiles, using a single, physically controlled parameter. Crucially, LCWCE decouples tip geometry from fiber type, taper length, and taper angle, allowing application-specific optimization that is not achievable with thermal pulling or breaking methods, while remaining low-cost, scalable, and compatible with standard laboratory equipment.

To our knowledge, no existing fabrication method simultaneously satisfies the key requirements for widespread adoption, precision, reproducibility, versatility, accessibility, and scalability. This gap likely explains why many promising lensed-fiber concepts demonstrated in earlier literature have not translated into broad community use.

Reviewer comment 2: The temperature gradient shown in **Figure 1c** is likely an artifact of the simulation setup. The boundary conditions assume fixed temperature and a very small simulation domain, which artificially amplifies the temperature gradient. In reality, due to heat conduction and convection in an open environment, the fiber tip region would be nearly isothermal. The simulation results are therefore not credible and should not be used to support the proposed mechanism.

Author's response: Thank you for this insightful comment. We agree that the small simulation domain and fixed-temperature boundary conditions used in the original manuscript can exaggerate the apparent temperature gradient, as such constraints underestimate heat dissipation in an open environment. We therefore substantially revised the numerical framework and its presentation to address this concern directly.

Our initial three-dimensional simulation was performed to determine the steady-state optical emission and the resulting temperature distribution near the fiber tip and, using Eq. (1) under experimentally relevant laser power levels, to compute the absolute local temperature required for evaluating the etching-rate ratio via the Arrhenius relation. Because the Arrhenius kinetics depend on the *absolute temperature* rather than only on relative gradients, obtaining a physically realistic apex temperature was essential. For this purpose, a 3D thermal model that captures heat spreading in all spatial directions was employed. While fixed-temperature boundaries can influence the far-field temperature decay, their impact on the local peak temperature at the apex is modest, making the 3D model suitable for estimating the absolute etching rate.

To obtain a more physically realistic axial temperature profile along the taper under limited computational resources, we subsequently performed a two-dimensional steady-state heat-transfer simulation with a substantially enlarged liquid domain. The outer boundary was positioned more than 200 μm from the heating region (>66× the effective heating diameter), ensuring convergence of the surface temperature gradient. Instead of fixed-temperature boundaries, we applied an effective heat-flux boundary condition based on experimentally validated microscale Nusselt correlations. This approach captures heat dissipation from micron-scale hotspots without artificially constraining the temperature field and yields a stable, converged temperature gradient along the fiber surface. Sensitivity analysis shows that further expansion of the domain changes the surface temperature gradient by less than 5%.

Beyond estimating the etching-rate profile along the taper, we incorporated these temperature-derived etching-rate kernels into a reduced-order surface-evolution model to simulate the time-dependent geometry evolution. The resulting numerical predictions quantitatively reproduce the experimentally observed apex recession, curvature increase, and shape transitions, providing independent validation of the proposed LCWCE mechanism.

Regarding the reviewer's expectation of a nearly isothermal tip region, we would like to clarify that this intuition applies to bulk-scale heat transfer, where buoyancy-driven convection dominates. In contrast, when the heating region is confined to the micrometer scale, buoyancy effects are strongly suppressed and heat removal is governed by conduction and microscale natural convection, which exhibit markedly different behavior. In this regime, effective heat-transfer coefficients are substantially enhanced, yet temperature gradients remain highly localized near the heat source. Such behavior has been widely reported for micron-scale heaters in liquids and is captured by the microscale correlations adopted in our revised model. The localized axial temperature gradient is therefore a physical consequence of microscale heating rather than an artifact of the simulation setup.

We have revised the manuscript to clearly distinguish between bulk and microscale heat-transfer regimes, replaced the original boundary-limited analysis with the physically justified heat-flux formulation, and expanded the Methods and Supplementary Information to document the numerical assumptions and validation in detail. We believe these revisions fully address the reviewer's concern and strengthen the credibility of the simulation-based support for the proposed etching mechanism.

Reviewer comment 3: The central claim of the paper, i.e., the fabrication of parabolic fiber tips, is not validated by any quantitative shape analysis. The authors rely on visual inspection and SEM images rather than precise geometric fitting. Without such analysis, it is impossible to confirm that the tips are indeed parabolic rather than hyperbolic, elliptical, or simply rounded.

Author's response: We thank the reviewer for this constructive and important comment. We fully agree that rigorous quantitative shape analysis is required to substantiate claims regarding parabolic, or more generally, non-spherical, fiber-tip geometries.

In the original manuscript, our use of the term *parabolic* followed a convention commonly adopted in the fiber-optics literature, where "parabolic" or "near-parabolic" tips are defined in a curvature- and performance-based sense, rather than by strict conic constant ($K= -1$). In this context, parabolic microlenses describe fiber tips with a shortened axial radius of curvature compared to spherical caps, leading to stronger focusing, reduced working distance, and enhanced coupling or collection efficiency relative to melt-formed hemispherical tips. This usage is well established in prior work on microlensed fibers, where the emphasis is on optical performance rather than exact mathematical classification.

Consistent with this convention, we previously employed a dimensionless geometric metric, a/b, where b is the mode-field radius and a is the axial sag measured from the point at which the taper radius equals b to the apex. For a spherical cap, $a/b = 1$, whereas values $a/b > 1$ indicate reduced axial curvature characteristic of aspheric, parabolic-like microlenses. The fabricated tips in this

work consistently exhibited a/b ≥ 1 and demonstrated optical focusing behavior consistent with parabolic microlenses reported in the literature (Bouhafs et al., 2022; Piccirillo et al., 2022.)

However, we acknowledge that this description alone does not provide a rigorous geometric classification and that quantitative conic analysis is necessary for clarity and for validating the claimed advancement over existing fabrication approaches. To directly address this concern, we have substantially revised the manuscript to include explicit conic fitting of experimentally extracted tip profiles.

Specifically, we now extract the full tip geometry from high-resolution SEM images and fit the profiles to the general conic sag equation. For each fiber tip, we report the radius of curvature, conic constant (K), and goodness-of-fit metrics, with $R^2 \geq 0.9995$ across all analyzed cases. This analysis unambiguously distinguishes between hyperbolic ($K < -1$), parabolic ($K \approx -1$), prolate elliptical ($-1 < K < 0$), and near-spherical ($K \approx 0$) geometries.

Importantly, this quantitative fitting demonstrates that LCWCE does not merely produce "rounded" tips but enables deterministic access to the full family of aspheric conic geometries, including hyperbolic, parabolic, and prolate elliptical profiles. These results are now presented in the revised Figures 1 and 4 and detailed in the Supplementary Information.

We believe that this systematic geometric verification directly resolves the reviewer's concern and significantly strengthens the technical rigor of the manuscript by replacing qualitative inspection with quantitative, reproducible shape classification.

[1] Bouhafs, Zaied, et al. "Parabolic microlensed optical fiber for coupling efficiency improvement in single mode fiber." *Optics Continuum* 1.5 (2022): 1218-1231.
[2] Piccirillo, Anna Maria, Luisa Toscano, and S. Toscano. "Blow-up results for a class of first-order nonlinear evolution inequalities." *Journal of Differential Equations* 212.2 (2005): 319-350.

Reviewer comment 4: Even if we assume that the proposed temperature gradient exists (i.e., higher temperature at the sides and lower at the tip), the resulting shape would more likely be hyperbolic, not parabolic. This is exactly contrary to the central claim of the paper.

Author's response: Thank you for this comment. We acknowledge that the original three-dimensional simulation employed a minimal computational domain with a fixed-temperature boundary positioned relatively close to the fiber tip, which led to a slight displacement (~2 μm) of the apparent temperature maximum from the apex. This displacement reflects a boundary-induced artifact of the simplified model rather than the true physical heating profile in an open liquid environment.

Importantly, even under this limitation, the simulation does not indicate sidewall-dominated heating. The heating remains highly localized near the fiber tip region and decays rapidly along the taper. The heating is therefore apex-centered, not sidewall-dominated. Because the total etched length during lens formation spans tens to hundreds of micrometers, this localized heating region cannot be interpreted as lateral heating that would preferentially remove material away from the tip. Accordingly, the reviewer's assumption of higher sidewall temperature is not consistent with the simulated conditions used to support the proposed mechanism.

The purpose of the original simulation was to support the physical hypothesis that localized optical absorption near the tip generates an axial etching-rate gradient, rather than to quantitatively predict the final lens geometry. We agree, however, that this distinction must be made clearer. In the revised manuscript, we therefore replaced the boundary-limited 3D model with an improved and physics-separated multiphysics framework and added quantitative geometric validation, which together remove ambiguity associated with the initial approximation. As discussed in our response to Comment 3, the revised analysis directly confirms the resulting geometry evolution through conic fitting.

We believe this clarification resolves the reviewer's concern while preserving the validity of the original mechanistic interpretation and substantially strengthening the rigor and transparency of the revised manuscript.

Reviewer comment 5: Furthermore, if the hypothesis holds, the shape of the fiber would evolve from conical to parabolic, and the optical (and thermal) field would change accordingly. It is unclear how the condition for temperature distribution is maintained throughout this process, particularly for multimode fibers with significant shape variations. In fact, the results for multimode fibers (Figs. 3a–3c, 4a–4b) show profiles that clearly deviate from a parabola, contradicting the paper's central claim while still being labeled as "PML."

Author's response: Thank you for this important comment. We agree that, in principle, the optical emission profile and the associated temperature distribution must evolve as the fiber geometry changes during etching, and that this coupling must be treated carefully to avoid ambiguity. We address the reviewer's concerns in three parts below.

(1) Temperature distribution during geometry evolution

We do not assume that the temperature distribution remains strictly invariant as the geometry evolves. Rather, the key physical requirement of the LCWCE mechanism is that a monotonic axial temperature gradient persists, such that the etching rate is highest near the apex and decreases with distance along the taper. Even as the tip shape evolves, this condition remains valid as long as optical absorption remains localized near the tip region.

Importantly, the hypothesis does not require the exact temperature profile to be identical at all times; it requires that higher etching persists at the apex relative to regions farther away, which is sufficient to drive controlled tip rounding and curvature evolution. This principle holds even when the detailed optical field redistributes due to geometric changes.

We agree with the reviewer that this point must be demonstrated clearly. Because fully coupled, time-dependent optical–thermal–geometric simulations are computationally prohibitive, we addressed this in the revised manuscript using a staged multiphysics approximation. Specifically, we performed steady-state wave-optics and heat-transfer simulations for three representative geometrical stages extracted from the experiment. The resulting temperature and etching-rate kernels show only modest variation across stages and consistently preserve the apex-localized gradient. This explicitly validates that the core condition for higher apex etching remains satisfied throughout geometry evolution (new Fig. 2, 3; Methods and Supplementary Information).

(2) Geometry evolution and computational limitations
We fully acknowledge that a fully self-consistent simulation continuously updating optical emission, temperature, and geometry would be ideal. However, such a simulation would require resolving sub-micron optical fields, microscale heat transport, and evolving boundaries over long time scales, which is not feasible under laboratory-scale computational resources.

To address this rigorously within practical limits, we therefore employed the three-stage reduced-order multiphysics strategy in the revised manuscript. This approach captures the dominant physics governing geometry evolution while avoiding unsupported assumptions of invariant temperature fields. We now explicitly state these assumptions and limitations, and we demonstrate consistency between staged simulations and experimentally observed geometry evolution.

(3) Multimode fibers and clarification of terminology (PML)
We agree with the reviewer that multimode fibers fundamentally differ from single-mode fibers in how optical fields interact with the tip geometry. Multimode fibers support a broad distribution of propagation angles and modal content, making the definition of a single "parabolic" or idealized conic geometry less meaningful from a strict optical standpoint.

In light of this, and based on our expanded geometric analysis, we have revised the terminology throughout the manuscript. In the revised version, we no longer label lenses fabricated on fibers as "PML." Instead, we use the more general and accurate term "microlens." This change eliminates potential misinterpretation.

Crucially, the new conic fitting and goodness-of-fit analysis demonstrates that LCWCE provides predictable and tunable access to diverse conic geometries. For multimode fibers, the significance lies not in enforcing a strict parabolic classification, but in the ability to reproducibly sculpt controlled microlens geometries that optimize optical performance for specific applications.

In summary, the revised manuscript:

- Explicitly demonstrates that an apex-localized axial temperature gradient persists during geometry evolution using staged multiphysics simulations,
- Clarifies the computational rationale and limitations of the modeling approach,
- Corrects and refines terminology to avoid misinterpretation for multimode fibers, and
- Reframes the central claim from "strict parabolic lenses" to deterministic, predictable microlens geometry control across fiber types.

We believe these revisions directly address the reviewer's concerns and significantly strengthen the physical clarity, rigor, and internal consistency of the manuscript.

Reviewer comment 6: The statement, "The confinement was experimentally verified… marking the first experimental demonstration of subwavelength beam confinement… of lensed fiber in water," overstates the significance of this work. Many previous studies have achieved comparable subwavelength beam confinement in similar settings, for example: DOI: 10.1002/smll.201770078; DOI: 10.1002/smll.201770078; DOI: 10.1021/acs.nanolett.1c04075.

Author's response: We thank the reviewer for pointing out these important references. We agree that previous studies have demonstrated strong light confinement from the lensed fibers, and we acknowledge that these studies have made meaningful contributions by showcasing the capabilities of lensed fibers and motivating further development in the field.

However, to the best of our knowledge, none of the cited studies, nor other related works, reports direct experimental measurements of the focused beam profile at the fiber tip in a liquid environment. In particular, quantitative measurements of beamwidth, focal waist, working distance, lateral FWHM, or axial emission distribution in water or similar media are not typically provided. In many prior demonstrations, beam confinement is inferred indirectly from trapping behavior rather than from direct experimental imaging of the emitted beam in liquid. While several studies include numerical simulations of the emission profile, these simulations are often based on idealized or nominal geometries, which may differ from the exact tip shapes produced experimentally. As a result, the simulated and experimental structures cannot always be compared in a one-to-one manner.

In contrast, our work provides direct experimental measurements of both the lateral and axial intensity distributions of the emitted beam in water, offering explicit evidence of subwavelength confinement at the lensed fiber tip. To avoid overstating our contribution, we have revised the manuscript to clarify that our intention is not to claim the first conceptual demonstration of subwavelength confinement from a tapered or lensed fiber. Rather, our contribution is in providing, to the best of our knowledge, the first direct experimental quantification of the beam profile, showing a subwavelength focal spot, measured in water. We believe this addresses an important gap in the existing literature by complementing prior conceptual and simulation-based studies with quantitative experimental evidence.

Reviewer comment 7: The authors emphasize that "the small refractive index contrast between fiber and medium… demands even greater accuracy in shaping for efficient focusing," and claim that "No other known fabrication method has demonstrated such precise, reproducible control over PML tips in high-refractive-index environments. " However, the fiber–water refractive index contrast is among the most common and well-studied cases, and the refractive index contrast between silica and water is not particularly challenging, making this assertion unsubstantiated.

Author's response: We thank the reviewer for raising this important point. We agree that the refractive index contrast between silica ($n \approx 1.45$) and water ($n \approx 1.33$) is among the most common and well-studied regimes in fiber optics, and we did not intend to suggest that this contrast is inherently unusual or intrinsically difficult. We have revised the manuscript to clarify this point and to remove any potentially misleading statements.

Our intended emphasis is that in moderate refractive-index-contrast environments, such as silica–water, the optical focusing and beam-shaping performance of a lensed fiber becomes particularly sensitive to the exact tip curvature and axial geometry, especially when tight confinement, short working distances, or high collection efficiency are required. Under these conditions, relatively small deviations in lens shape can lead to significant changes in focal position, spot size, and coupling efficiency. Consequently, precise and predictable control over transitions between

aspheric geometries, such as hyperbolic, parabolic, and prolate elliptical profiles, becomes important for optimizing performance.

Conventional melting- or arc-based fabrication methods inherently rely on surface-tension-driven reflow, which biases the tip geometry toward spherically rounded shapes. While non-spherical tips may occasionally arise from abrupt pulling or breakage events, such outcomes are not controllable or reproducible and therefore do not allow deterministic access to specific aspheric geometries with quantitative validation. In contrast, our chemical etching approach enables continuous, parameter-controlled shaping of the tip through an axial etching-rate gradient, providing predictable access to defined geometries that are difficult to achieve reliably with melting-based methods.

To clarify, we do not claim that other fabrication techniques are incapable of producing high-quality lensed fiber tips for operation in aqueous environments. Rather, our contribution lies in demonstrating a predictable, operator-independent, and reproducible fabrication route that enables controlled generation of specific tip geometries, including all aspheric tip geometry family and step-tapered structures, across different fiber types, operators, and laboratory settings.

We have revised the manuscript accordingly to reflect this distinction more accurately:

- Our approach emphasizes deterministic and reproducible geometry control,
- Whereas many conventional approaches, particularly thermal pulling, are highly system-dependent and difficult to reproduce across laboratories.

We appreciate the reviewer's comment, which has helped us clarify the scope of our claims and improve the precision and balance of the manuscript.

Reviewer comment 8: The authors claim a "major breakthrough in low-power optical trapping," stating that " our microparticle trapping power was 6 – 36 times lower than that of conventional heat-and-pull fiber tweezers." Yet, both the physical shape (Fig. 6c)
and the optical field distribution (Figs. 6d, 6g) of their tapered fiber are indistinguishable from conventional fiber tweezers. Variations in input/output power, detection methods, coupling efficiency, or transmission losses could easily account for the reported difference in trapping power, rather than any intrinsic improvement of the probe.

Author's response: We thank the reviewer for raising this critical point. As highlighted in the manuscript, lateral optical forces can be generated by typical tapered or rounded fiber tips; however, axial confinement and axial trapping forces are highly sensitive to the exact tip geometry, therefore, it is the most determining factor of the optical tweezer. Achieving this requires tight control over the curvature and shape of the tip. Although simulations of conventional heat-and-pull fibers in previous literature also exhibited strong confinement, our concern is that thermal pulling does not offer reliable or predictable control over the final geometry. Sudden breakage, sensitivity to filament condition, and system-dependent heating profiles often result in tips with irregular or asymmetric end-faces, as can be observed in several of the cited studies.

Importantly, none of the referenced studies reports experimental measurements of the emitted field distribution in a liquid environment. In those works, field profiles are inferred exclusively from

numerical simulations based on idealized or nominal tip geometries, which may deviate from the actual structures produced experimentally. Such discrepancies can lead to optimistic estimates of focal confinement and field gradients.

In contrast, our analysis is performed using the exact fiber geometry measured experimentally, and the corresponding lateral and axial intensity distributions are directly characterized in water. As a result, the measured field profiles are intrinsically consistent with the fabricated structure rather than relying on assumed geometries. To make this correspondence explicit, the revised **Fig. 8** includes SEM images of the specific fiber used for optical trapping, together with the corresponding geometric fits, ensuring a direct link between fabricated structure, simulated emission profile, and optical performance.

Regarding the reported trapping power, we agree that variations in coupling, transmission, and detection can influence power measurements. For this reason, we did not rely on reflected-power measurements alone. Prior to any trapping experiments, we calibrated the total output power delivered at the fiber tip, fully accounting for coupling efficiency, 2×2 coupler splitting ratio, detector calibration, and round-trip transmission.

Although system-level uncertainties can introduce modest deviations, they cannot account for the observed 6–36× reduction in trapping power. This order-of-magnitude improvement significantly exceeds what can be attributed to measurement variation alone and instead reflects the intrinsic optical behavior of the precisely controlled tip geometry, consistent with our simulations.

Finally, we note that many prior publications also report absolute trapping powers, demonstrating that such measurements are meaningful and comparable when performed under well-calibrated conditions.

Reviewer comment 9: Several figures lack scale bars.
Author's response: We thank the reviewer for this observation. For subfigures that were acquired under the same magnification, a single scale bar is provided to avoid unnecessary repetition. To ensure clarity, we have explicitly stated this in the corresponding figure captions so that readers can easily interpret the dimensions across all related subfigures.

Reviewer 2.

Response to the Reviewer's Overall Statement

Reviewer's Overall Statement: "This manuscript by Kim et al. presents a significant advancement in fiber optic technology: Laser-Controlled Wet-Chemical Etching (LCWCE) for fabricating parabolic micro-lensed fibers (PMLFs). The method achieves unprecedented sub-micron control over lens shape and working distance, overcoming long-standing limitations of conventional techniques in precision, reproducibility, and scalability. The authors thoroughly demonstrate the superiority of their PMLFs in two demanding applications. First, they achieve record-low power optical trapping of nanoparticles and microparticles, highlighting highly efficient focusing. Second, they demonstrate groundbreaking in vivo neural interfacing, enabling non-invasive, all-optical single-cell recording and modulation at depths >1.5 mm with exceptional spatial resolution and signal-to-noise ratio. The technique's versatility across fiber types, low cost, and batch-fabrication potential make it highly impactful. This work effectively bridges a critical fabrication gap and establishes a transformative platform for biophotonics, neuroscience, and precision optical technologies."

Author's response to the Overall Statement: We sincerely thank the reviewer for this thoughtful and encouraging assessment of our work. We appreciate the recognition of both the technical advances enabled by LCWCE and its broader implications for fiber-based micro-optics and biophotonics.

In response to the review process, we have substantially revised the manuscript to further strengthen the rigor, clarity, and generality of the presented results. In particular, we have added physics-separated multiphysics modeling, systematic geometric validation across the full conic family, and explicit reproducibility analyses to support the deterministic nature of the fabrication process. We have also refined the presentation to more clearly distinguish between methodological advances and application demonstrations, ensuring that the scope and claims are appropriately framed.

We are grateful for the reviewer's recognition that LCWCE addresses long-standing challenges in precision, reproducibility, scalability, and accessibility that have historically limited the broader adoption of micro-lensed fibers. We agree that the demonstrated applications, minimal power optical trapping and minimally invasive, depth-resolved neural interfacing, serve as representative examples of how predictable and tunable tip geometry can unlock new capabilities, rather than as the sole focus of the work.

Overall, we appreciate the reviewer's perspective that LCWCE constitutes a platform-level advance rather than an incremental fabrication refinement. We believe the revised manuscript now more clearly conveys how this approach bridges a critical gap between conceptual micro-optical designs and practical, reproducible fabrication, with relevance extending beyond the specific demonstrations presented here.

We thank the reviewer for their constructive engagement and for helping us improve both the technical depth and the broader framing of the manuscript.

Response to the Reviewer's Detailed Statement

Reviewer comment 1: Suggest the authors consider explicitly mentioning the key applications (optical trapping, neural recording) and the achieved performance metrics (e.g., trapping power, spatial resolution) in the abstract to immediately highlight the practical impact.

Author response: We thank the reviewer for this constructive suggestion. In response, we have revised the Abstract to explicitly highlight both the key application domains and the achieved performance metrics. Specifically, we now directly mention fiber-based optical tweezers and in vivo neural interrogation as representative applications, and we report milliwatt-scale, minimal trapping power as well as non-invasive dendritic detection and single-cell neuronal interrogation at depths exceeding 1.5 mm in brain tissue. These additions were made to ensure that the practical impact and functional significance of the proposed LCWCE approach are immediately clear to a broad readership.

Reviewer comment 2: Suggest the authors enhance specify the wavelength at which the "beamwidth of 24.4 μm" was measured for consistency with other beamwidth reports.

Author response: We thank the reviewer for this helpful suggestion. While the excitation wavelength was previously specified in the figure caption, it was not explicitly stated in the main text. We have now revised the manuscript to clearly indicate that the beamwidth of 24.4 μm was measured at an excitation wavelength of 488 nm and that the reported value corresponds to the full width at half maximum (FWHM) of the illumination profile. This revision ensures consistency with standard beamwidth reporting practices and facilitates direct comparison with prior studies. To ensure, the large beamwidth of 24.4 μm of the MLF is attributed to the large core diameter and its high NA, supporting multimodes (high total internal reflection angle of the guided mode), resulting in the extension of the beamwidth.

Reviewer comment 3: Suggest the authors enhance the part about more detail on how the initial taper angle quantitatively influences the resulting temperature profile and, consequently, the final lens curvature.

Author response: We thank the reviewer for this insightful comment. In the revised manuscript, we clarify that the initial taper angle influences the final lens curvature primarily through its effect on how a given apex-localized temperature field maps onto the evolving geometry, rather than through substantial changes in the absolute temperature profile itself.

Our steady-state wave-optics and heat-transfer simulations show that, for the range of taper angles studied, the surface temperature profiles extracted along the surface arc length are nearly identical (Fig. S3). This indicates that the laser-induced heating mechanism and the resulting axial temperature decay are largely insensitive to taper angle. Consequently, the local etching-rate kernel derived from temperature via Arrhenius kinetics is similar across different tapers.

The observed taper-angle dependence of the final lens geometry therefore arises from geometric effects: the same apex-localized etching-rate distribution acts on different initial cone angles. For smaller taper angles, the initial geometry converts this localized etching into rounding that extends

over a longer axial distance relative to the original profile, allowing curvature to redistribute over a larger portion of the tip and enabling transitions toward parabolic and prolate elliptical geometries. In contrast, steeper tapers map the same etching profile onto a shorter effective axial region, concentrating curvature near the apex and favoring the persistence of hyperbolic shapes.

We have revised the manuscript to explicitly articulate this distinction between temperature-field generation and geometry-dependent curvature redistribution, thereby clarifying how taper angle governs the final lens shape without requiring taper-specific temperature profiles. This clarification strengthens the physical interpretation of the experimental trends while remaining consistent with the scope and assumptions of the numerical framework.

Reviewer comment 4: Suggest the authors enhance present quantitative data on the reproducibility of the parabolic lens shape across multiple batches.

Author's response: We thank the reviewer for this important comment. As clarified in our response to Reviewer 1, the term *parabolic* in the original manuscript followed a convention widely used in the fiber-optics literature, where parabolic or near-parabolic microlenses are defined based on curvature evolution and optical performance rather than strict mathematical conic classification. Within this established framework, our initial emphasis was on demonstrating reproducible access to high-performance, aspheric lens geometries under controlled LCWCE conditions.

In the revised manuscript, following the reviewers' suggestions, we expanded the geometric analysis to include systematic conic fitting across taper angles and etching conditions. This deeper analysis reveals that LCWCE does not produce a single fixed parabolic geometry, but instead enables predictable and continuous access to hyperbolic, parabolic, and prolate elliptical regimes, with the resulting geometry governed primarily by the initial taper angle and etching progression (**Fig. 4b**).

To address reproducibility quantitatively in a physically meaningful manner, we focus on experimentally robust geometric metrics, such as apex radius and curvature evolution, which directly reflect the underlying etching physics and are far less sensitive to fitting artifacts. Across multiple fiber tips fabricated under identical conditions, the apex radius was measured to be 1.49 ± 0.24 μm ($n = 6$), demonstrating high consistency in the primary geometric parameter governing optical performance.

We note that some spread in fitted conic descriptors is observed even for tips with nearly identical curvature radii (**Figs. 4b and S2**). This behavior reflects the dependence of conic classification on the specific geometric criteria used to describe the tip profile, rather than variability in the underlying fabrication outcome. To our knowledge, no prior fiber-based fabrication method has reported systematic mapping of conic geometry regimes or deterministic access to hyperbolic, parabolic, and prolate elliptical tip shapes. In this context, the consistent positioning of the fitted geometry near the parabolic boundary ($K \approx -1$) and, more importantly, the predictable trend with increasing tip radius shown in **Fig. 4b** represent the scientifically meaningful result, rather than the exact numerical dispersion of individual fitted values.

We have revised the manuscript to clarify this distinction and to emphasize that LCWCE provides predictable, physics-driven control over lens geometry regimes, while absolute equality of fitted conic parameters is neither expected nor required to demonstrate reproducibility or functional performance. These revisions strengthen the quantitative interpretation of our results without altering the experimental conclusions.

Reviewer comment 5: Suggest the authors enhance the part about tests performed to assess the long-term durability of these lenses.

Author response: We thank the reviewer for raising this important point. The fabricated lenses are monolithic fused-silica structures, identical in material composition to standard optical fibers. LCWCE removes material through controlled chemical etching but does not introduce additional materials, coatings, interfaces, or residual stresses that could compromise long-term stability. As a result, the durability of the MLFs is fundamentally governed by the intrinsic stability of silica glass, which is well established in optical, biomedical, and telecommunication applications.

Unlike polymer-based or hybrid micro-optics, which may suffer from swelling, photodegradation, or delamination in aqueous or biological environments, LCWCE-fabricated lenses consist entirely of bulk glass and therefore do not exhibit time-dependent material degradation under normal operating conditions. After fabrication, the fibers are thoroughly rinsed and removed from the etchant, and no ongoing chemical processes remain. Many of the demonstrated results were analyzed first under the microscope, and the high-resolution imaging or confocal analysis of emission profile was conducted at least a few days or a month later after the fabrication, which is beyond ongoing chemical process time.

In addition, the same LCWCE-fabricated fibers were reused across multiple optical trapping and biological experiments without observable changes in optical performance or tip morphology. We have clarified this point in the revised manuscript to explicitly state that long-term durability is ensured by the monolithic glass nature of the lenses and does not require additional aging tests beyond those already established for silica fiber devices.

Reviewer comment 6: Suggest the authors enhance the part about the experimentally measured beam profile and trapping efficiency compared to simulations of an ideal parabolic lens of the same dimensions.

Author response: The reviewer requests stronger experimental validation of the beam profile and trapping efficiency relative to simulations of an ideal parabolic lens. Direct experimental characterization of the optical emission from the HMLF at the non-visible near-infrared (976 nm) is not experimentally accessible, because fluorescence-based detection is not available at these wavelengths. Instead, we experimentally characterized the effective illumination profile using confocal fluorescence imaging in water, which directly reflects the intensity gradients relevant for optical trapping. These measurements were performed on the same probes whose geometries were independently characterized by SEM.

In the revised manuscript, we further strengthened the comparison by performing optical simulations using the experimentally measured tip geometries using SEM rather than relying on the high magnification optical microscope image. We directly compared LCWCE-fabricated hyperbolic microlenses with conventionally fabricated heat-pulled and chemically etched lenses of comparable dimensions. This geometry-matched simulation approach enables meaningful comparison between experiment and theory.

Trapping efficiency was experimentally quantified using the minimum optical power required for stable particle trapping, a widely used performance metric in fiber-based optical tweezers. The substantially reduced trapping power observed for LCWCE-fabricated lenses is consistent with the enhanced axial and transverse force gradients predicted by the simulations, thereby validating the role of geometry-controlled field confinement.

Reviewer comment 7: To further solidify this, did you perform post-hoc histology to confirm the physical location of the fiber tip relative to the recorded neuron(s) and verify that the recorded signals indeed originated from a single, identified cell body rather than a small, unresolved cluster?

Author's response: We thank the reviewer for raising this important point regarding anatomical verification.

(1) Post-hoc histology

Conventional post-hoc histological confirmation of the fiber tip location was not feasible in this study due to the extremely small probe diameter (<25 μm), which further tapers to <15 μm at the tip. Unlike conventional electrodes or larger optical probes, implantation of such ultrathin fibers does not produce a visible lesion or track in fixed tissue. Although we attempted dye-assisted marking by soaking the fiber prior to implantation, labeling was confined to superficial regions and did not reliably delineate the deep implantation trajectory or final tip position (**Fig. 10**).

(2) Independent validation of spatial resolution

To directly assess whether the probe can resolve signals at the single-cell or subcellular scale, we performed controlled *in vitro* experiments in brain slices. Using fluorescent labeling, we demonstrated that the MLF can spatially distinguish fine neuronal structures, including dendritic and axonal branches, consistent with the expected optical resolution of the probe. (**Fig. S7**) These experiments provide an independent benchmark for the spatial selectivity of the recordings.

(3) Depth-resolved reproducibility *in vivo*

During *in vivo* recordings, we advanced and retracted the probe along the dorsoventral axis and observed reproducible signal peaks appearing at the same depth across repeated passes (**Fig. S11**). The consistent re-emergence of these signals at identical anatomical depths strongly supports a localized biological origin rather than motion artifacts, optical cross-talk, or unresolved clusters of cells.

Taken together, while direct post-hoc histological confirmation is technically challenging for probes of this scale, the combination of *in vitro* spatial-resolution validation and depth-resolved reproducibility *in vivo* provides strong evidence that the recorded signals originate from spatially localized neuronal structures rather than from diffuse or unresolved cell populations.

Although direct post-hoc histological localization of the fiber tip is technically challenging for ultrathin probes (<25 μm diameter), the combination of independent in vitro spatial-resolution validation and depth-resolved reproducibility observed in vivo provides strong evidence that the recorded signals originate from spatially localized neuronal structures.

Reviewer #4

Response to the Reviewer's Overall Statement

Reviewer comment: "The submitted manuscript proposes a fabrication method for microlensed fibers based on laser-controlled wet chemical etching (LCWCE). The study introduces a potentially useful approach that may be of interest to researchers working on optical fiber microstructuring and related applications. However, while the method could have value in specialized contexts, the manuscript in its current form does not meet the standards of broad conceptual impact and presentation quality expected for Nature Communications. The work would benefit from substantial revision to enhance the accuracy, clarity, and precision of both the language and the technical explanations, as well as from a more comprehensive contextualization within the existing literature. After these improvements, the paper may be more appropriate for publication in a specialized optics or photonics journal. "

Author's response to the overall statement: We thank the reviewer for this careful assessment. In response, we have substantially revised the manuscript to improve clarity, rigor, and contextualization, and, most importantly, to articulate the broader conceptual contribution of the work. We respectfully submit that the revised manuscript now goes well beyond a technical fabrication improvement and instead introduces a general, physics-based framework for optically regulated chemical shape formation, with relevance extending beyond fiber optics.

Below, we summarize the conceptual advances and their broader impact.

(1) Conceptual advance: a new mechanism for micro-optical shaping

All established microlensed-fiber fabrication techniques, including thermal pulling, CO_2-laser melting, and arc-discharge reshaping, rely on thermomechanical deformation governed by globally applied heat. These methods inherently couple curvature, diameter, taper angle, and taper length, limiting reproducibility and preventing deterministic access to curvature-defined or application-specific geometries.

In contrast, our work introduces a fundamentally different mechanism: laser-controlled wet-chemical etching, in which localized optical emission from a designed initial geometry generates a microscale temperature field that deterministically governs spatially varying etching rates. Geometry evolution is thus regulated by reaction kinetics rather than surface-tension-driven reflow.

By explicitly designing the initial structure to control the optical emission profile, and predicting how this emission produces a temperature field and an etching-rate kernel, we achieve controlled, stepwise evolution of the geometry. This reframes micro-optical shaping from a thermal deformation problem into a laser-regulated chemical–thermal feedback process, representing a clear conceptual shift in microstructuring.

Importantly, this principle is not confined to optical fibers; it establishes a generalizable strategy for chemically mediated, optically programmed geometry evolution in microscale structures.

(2) Physics-based validation through a new multiphysics framework

In direct response to concerns regarding rigor and validation, we have introduced a quantitative multiphysics framework that explicitly links:

- wave-optical electromagnetic emission and absorption,
- steady-state microscale heat transfer, and
- temperature-dependent wet-etch kinetics.

These elements are integrated through a reduced-order moving-boundary model that quantitatively reproduces the experimentally observed geometry evolution (new Figs. 2–3 and Supplementary Information).

To our knowledge, prior laser-assisted wet-etching studies have not combined wave optics, microscale heat transport, and Arrhenius-governed chemical kinetics into a single predictive framework for geometry evolution. Existing literature typically treats laser-assisted etching empirically or assumes simplified thermal fields without coupling to evolving geometry. The framework introduced here, therefore, provides first-principles validation of the LCWCE mechanism and elevates it from phenomenology to predictive design.

3. Rigorous geometric verification beyond qualitative labeling

The revised manuscript now includes systematic conic fitting of experimentally extracted tip profiles, reporting curvature radius, conic constant, and goodness-of-fit metrics. This analysis demonstrates deterministic access to a family of aspheric geometries (hyperbolic, parabolic, and prolate elliptical), rather than a single idealized shape.

This addresses a long-standing gap in the literature, where microlensed fibers are often labeled qualitatively without quantitative conic analysis. Accordingly, we have revised terminology throughout the manuscript to avoid ambiguity and to align claims strictly with quantitative evidence.

4. Broad conceptual relevance beyond fiber optics: inverse, geometry-aware etching

Laser-assisted wet etching itself is not new; previous studies have used laser heating in liquid etchants to create localized grooves, pits, or recesses on planar substrates or flat fiber surfaces. However, these approaches are almost exclusively forward and empirical: laser exposure defines where material is removed, but not what three-dimensional geometry will result.

Crucially, prior work has not addressed the inverse problem, how to determine the initial geometry and optical conditions required to achieve a desired final shape. Geometry evolution is rarely modeled, and the link between initial structure, optical field, thermal response, and etched outcome remains implicit.

In contrast, the framework introduced here explicitly enables backtracking from a target final geometry to the required initial structure and processing conditions. By coupling wave-optical emission, microscale heat transfer, and reaction kinetics, we show how spatially resolved etching-rate kernels emerge and how these kernels deterministically sculpt geometry over time. This

transforms laser-assisted wet etching from a patterning tool into a rational, geometry-aware shape-engineering methodology.

This inverse-design capability is not limited to fiber tips. The same logic applies to laser-regulated chemical structuring of micro-optics on planar substrates, etched photonic components, microfluidic interfaces, chemical sensors, and glass or semiconductor microdevices.

5. Broad impact across scientific fields

Although demonstrated here using fiber microlenses, the underlying principle, laser-controlled, spatially selective wet-chemical etching with predictive geometry evolution, has relevance across multiple domains, including:

- optical tweezers and particle manipulation,
- minimally invasive neurophotonic probes,
- micro-optics and integrated photonics,
- fiber-based micro-endoscopy,
- microfluidic sensing and chemical detection,
- fiber–chip coupling, and
- near-field and nonlinear microscopy.

By enabling high-curvature and aspheric geometries that are inaccessible to thermomechanical methods, this approach substantially expands the design space of micro-optical devices. The conceptual contribution is therefore inherently interdisciplinary and extends beyond a photonics sub-specialty.

In summary, the revised manuscript introduces (i) a new physical mechanism for micro-optical shaping, (ii) a predictive multiphysics framework for laser-regulated chemical geometry evolution, and (iii) a general inverse-design strategy for etched microstructures. We respectfully submit that these advances constitute a broad conceptual contribution aligned with the scope and standards of *Nature Communications*.

Response to the Reviewer's Detailed Statement

Reviewer comment 1: Abstract: it is not clear what "longitudinal” exactly means at this early point in the manuscript. I suggest the authors reword to better convey the intended concept

Author’s response: Thank you for this comment. We agree that the term “longitudinal” may be ambiguous at the Abstract level, particularly given that the fiber geometry evolves during the etching process. Upon revision, we recognized that retaining this term could introduce inconsistency, as the spatial reference becomes less well-defined once the geometry departs from the initial taper.

To avoid potential confusion, we have removed the term “longitudinal” from the Abstract altogether and rephrased the description to directly reflect the evolving, apex-centered geometry without relying on a fixed directional qualifier. This revision ensures conceptual clarity and consistency with the dynamic nature of the geometry evolution described in the manuscript.

Reviewer comment 2: Two-photon lithography (TPL), which is highly relevant in this context, is only indirectly addressed through a few application-focused references (e.g., 10 and 14) but is not explicitly mentioned among the fabrication methods for microlensed fibers (MLFs) in the Introduction (page 2). It should be included there for completeness and fairness in presenting the state of the art, especially since TPL is a direct competing technique that offers a much greater degree of freedom in controlling the geometrical shape compared to the other fabrication methods discussed.

Author's response: We thank the reviewer for this important suggestion and agree that two-photon lithography (TPL) is a highly relevant fabrication technique that should be explicitly acknowledged for completeness and fairness in presenting the state of the art.

In the revised manuscript, we have added an explicit discussion of TPL in the Introduction as a competing and complementary approach for fabricating microlensed fiber tips and complex micro-optical structures. We now clearly recognize that TPL offers exceptional geometric freedom, enabling arbitrary three-dimensional shapes with sub-micrometer resolution, and that it represents one of the most powerful tools currently available for custom micro-optics fabrication.

At the same time, we have clarified the distinct positioning of LCWCE relative to TPL, rather than presenting LCWCE as a replacement. While TPL excels in geometric versatility, it typically involves:

- specialized and costly instrumentation,
- photoresist-based fabrication followed by post-processing and material transfer,
- serial writing processes that limit throughput,
- and constraints on material choice and long-term robustness for in vivo or harsh chemical environments.

In contrast, LCWCE operates directly on standard silica fibers, preserves intrinsic material properties, and enables deterministic, reproducible geometry evolution through a single physical control parameter (etching time), without resist processing or serial writing. As such, LCWCE occupies a complementary regime: it trades unrestricted freeform design for scalability, material compatibility, robustness, and accessibility, which are critical for widespread adoption in fiber-based sensing, neurophotonics, and in vivo applications.

We have revised the Introduction accordingly to:

- explicitly include TPL among state-of-the-art fabrication methods for microlensed fibers,
- clarify its strengths and limitations in the context of fiber probes,
- and more clearly articulate the niche and conceptual contribution of LCWCE relative to existing techniques.

We appreciate the reviewer's comment, which has helped us improve both the completeness and the clarity of the manuscript's positioning within the broader microfabrication landscape.

Reviewer comment 3: pag 4: "This approach utilizes the light emitted from the fiber to locally heat the etchant, influencing its absorption and increasing the reaction rate […]". "Absorption" of

what? Like this it seems that you mean that light is absorbed, and then heats the etchant, and that in turns changes the absorption (so this supposedly has a temperature dependence)

Author's response: We thank the reviewer for highlighting this ambiguity. We agree that the original wording could be misinterpreted. Our intended meaning was not that the optical absorption itself depends on temperature, but rather that optical absorption in the etchant produces localized heating, which subsequently accelerates the chemical etching reaction through temperature-dependent (Arrhenius) kinetics.

We have revised the manuscript to clearly distinguish these processes. Specifically, the revised text now explicitly states that (i) laser light is absorbed by the etchant, (ii) this absorption generates localized heating near the fiber surface, and (iii) the resulting temperature rise enhances the etching rate via its Arrhenius dependence. These clarifications remove the previous ambiguity and accurately reflect the physical mechanism underlying LCWCE.

Revised manuscript (page 4, lines 26–29): *Among these, temperature control is particularly practical, leveraging the ability of silica fibers to guide and emit light that is absorbed by the surrounding etchant near the fiber tip, producing localized heating and thereby enhancing the local etching rate through Arrhenius-type temperature dependence.*

Reviewer comment 4: pag 4: "For instance, when the flat fiber end is immersed in the etchant and light with a high absorption coefficient is emitted from it, […]". Please rephrase as light does not have "a high absorption coefficient"

Author's response: We thank the reviewer for pointing out this imprecise phrasing. The original wording was ambiguous and could indeed be misinterpreted. Our intent was to indicate that the etchant exhibits strong substantial absorption at the emission wavelength, rather than implying that the light itself possesses an absorption coefficient.

We have revised the text to explicitly distinguish optical absorption by the etchant from the resulting laser-induced heating and subsequent temperature-dependent etching kinetics, thereby eliminating the ambiguity.

- **Original:** *For instance, when the flat fiber end is immersed in the etchant and light with a high absorption coefficient is emitted from it, a temperature gradient is created along the radial direction from the center.*
- **Revised (page 5, lines 1–7):** *For a flat-cleaved fiber, optical heating absorbed by etchant produces a predominantly radial temperature gradient with a maximum at the fiber center, which accelerates etching within the core region and leads to pit formation rather than convex shaping. In contrast, formation of a smooth ML requires an etching-rate gradient oriented along the fiber axis, with the highest rate near the apex and a rapidly decaying profile along the surface. Such an axial gradient enables simultaneous axial recession and controlled radial rounding, both of which are necessary to produce convex lens geometries suitable for optical focusing.*

Reviewer comment 5: pag 4: "This gradient causes a hole to form within the optical cores, further

degrading the fibers focusing and collection efficiency." At this stage, the fiber still has a flat end, so it is not clear what "focusing and collection efficiency" the authors are referring to

Author's response: We thank the reviewer for pointing out this ambiguity. We agree that, at this stage of the process, the fiber end remains flat and therefore does not yet provide optical focusing in the lensing sense. Our original wording was imprecise in this context.

Our intent was to convey that the formation of a central pit within the optical core degrades the intrinsic optical performance by perturbing the guided mode distribution and reducing effective light transmission and collection as the pit develops at the fiber tip.

To avoid confusion, we have revised the text accordingly (as described in our response to Comment 4), removing references to optical focusing at this stage and explicitly describing the physical consequences of radially localized etching on the evolving fiber geometry.

Reviewer comment 6: pag 4: "[…] follows a higher-order decay than linear." this sentence needs more precision in language. What's a "higher-order decay'?

Author's response: We thank the reviewer for pointing out this imprecise wording. We agree that the phrase "higher-order decay" was ambiguous and did not clearly convey the intended physical meaning.

Our intention was to emphasize that an axial etching-rate profile that decreases nonlinearly with distance from the apex, that is, more rapidly than a linear decay, is essential for generating a smoothly curved, convex tip geometry. However, we recognize that the term "higher-order decay" could be interpreted in multiple ways and therefore introduced unnecessary ambiguity.

In revising the manuscript, and supported by additional simulation results, we have removed this terminology and instead explicitly describe the decay as exponential, directly linking the observed nonlinearity to its physical origin in localized optical heating and temperature-dependent etching kinetics.

We appreciate the reviewer's comment, which helped improve both the clarity and physical accuracy of the description.

Reviewer comment 7: pag 4: "A chemically tapered initial structure offers two key advantages". "Advantages" vs other methods, or what? This is not clear

Author's response: We thank the reviewer for highlighting this point and agree that the basis of comparison should be stated explicitly. In the revised manuscript, the term "advantages" refers specifically to chemical etching starting from a flat-cleaved fiber end, not to other fabrication techniques.

As clarified in the revised text, forming a convex micro-lens requires an axially oriented etching-rate gradient, where the etching rate is highest near the fiber tip and decreases smoothly along the fiber surface. Flat-cleaved fibers subjected to chemical etching predominantly develop radial

temperature and etching-rate gradients across the end face, which favor pit or hole formation rather than controlled axial rounding and convex lens generation.

A chemically tapered initial fiber geometry naturally overcomes this limitation and offers two advantages relative to flat-cleaved fibers during chemical etching. First, tapering alters mode confinement so that optical emission becomes localized near the sharp fiber end, producing a micrometer-scale, axially varying temperature field as absorbed light heats the etchant. This directly establishes the required axial etching-rate gradient. Second, the tapered geometry enables continuous and stable shape evolution during etching, allowing the fiber profile to transition smoothly from conical to elliptical, parabolic, and ultimately spherical forms under constant illumination.

We have revised the manuscript to explicitly state this comparison and to clarify that these advantages arise from the initial tapered geometry within the context of wet chemical etching, thereby improving precision and avoiding ambiguity.

Reviewer comment 8: pag 4: what the exact mechanism for light absorption here? At this point in the manuscript, what absorbs and at what wavelength are not clear. Some of these info seems to be only provided in figure 1 caption (also: what laser power for that temperature map in Fig. 1c?) and later in the manuscript

Author's response: We thank the reviewer for pointing out this lack of clarity. We agree that the absorption mechanism and operating wavelength should be stated explicitly in the main text rather than inferred from figure captions or later sections.

To address this, we have revised the manuscript to clearly specify what absorbs the light and at which wavelength, as well as the resulting physical mechanism. The relevant sentence has been modified as follows:

- **Revised manuscript (Page 4, lines 26–28):** *Among these, temperature control is particularly practical, leveraging the ability of silica fibers to guide and emit light that is absorbed by the surrounding etchant near the fiber tip, producing localized heating and thereby enhancing the local etching rate through Arrhenius-type temperature dependence.*

- **Revised manuscript (Page 5, lines 18-21):** *Here, we employed 1550 nm as the source of the energy to increase the local temperature at the end of the fiber. This wavelength was intentionally selected because it lies within a strong absorption band of the etchant while remaining readily available from standard fiber lasers (Fig. 1a inset).*

This revision explicitly clarifies (i) that the etchant (water) is the absorbing medium, (ii) the operating wavelength (1550 nm), and (iii) that optical absorption leads to localized heating which enhances the etching rate through Arrhenius-type kinetics. We believe this resolves the ambiguity raised by the reviewer and improves the clarity and completeness of the manuscript.

Reviewer comment 9: pag 4: "gradual transformation […] structures." Is there any opto/therm/fluido-dynamic modeling that enables the description of this?

Author's response: We thank the reviewer for raising this important point. A fully coupled, time-dependent opto-thermo-fluidic simulation of the LCWCE process would, in principle, require simultaneous resolution of (i) sub-micrometer optical field distributions near the fiber tip, (ii) transient heat diffusion and convection in the surrounding liquid over length scales extending tens to hundreds of micrometers, and (iii) temperature-dependent chemical reaction kinetics on an evolving three-dimensional boundary. Resolving these disparate spatial and temporal scales within a single transient framework, particularly with a continuously evolving geometry, is computationally prohibitive with laboratory-scale resources, even in two dimensions.

For this reason, the original manuscript relied on simplified simulations to qualitatively illustrate the physical mechanism. In the revised manuscript, we have introduced a physics-separated multiphysics framework that captures the dominant physical processes governing geometry evolution while remaining computationally tractable.

Specifically, the revised model integrates (i) wave-optics simulations to determine spatial optical emission and absorption, (ii) steady-state microscale heat-transfer calculations to obtain the temperature distribution along the fiber surface, and (iii) temperature-dependent wet-etch kinetics to generate a spatially resolved etching-rate kernel. This kernel is then applied within a moving-boundary surface-evolution model that advances the fiber surface along its local normal direction.

This framework reproduces the experimentally observed gradual transformation from conical to elliptical, parabolic, and near-spherical geometries (new Figs. 2–3 and Supplementary Information), providing a mechanistic and predictive description of the shape evolution referenced in the manuscript. While not a fully coupled transient opto-thermo-fluidic simulation, the reduced-order and physics-separated approach captures the governing physics responsible for geometry evolution and offers clear physical insight into how optical heating drives controlled, continuous reshaping during LCWCE.

We have revised the manuscript to explicitly reference this modeling framework when discussing gradual structural transformation, thereby clarifying the physical basis of the description and addressing the reviewer's concern.

Reviewer comment 10: pag 5: "This equation describes the relative increase in the etching rate compared to the etching rate at room temperature (20°C) under varying incident power levels." Rephrase for the fact that equ (3) does not directly integrate laser power as a parameter

Author's response: We thank the reviewer for identifying this imprecise phrasing. The original sentence incorrectly implied that Eq. (3) explicitly depends on the incident laser power. In fact, Eq. (3) relates the local etching rate solely to the local temperature through temperature-dependent reaction kinetics.

The reference to incident power was intended to describe the experimental and simulated conditions used to generate the temperature profiles shown in the corresponding figure, not to suggest that laser power appears directly in the equation itself. To avoid this confusion, we have revised the manuscript to clearly distinguish between (i) laser power as an experimental and

simulation input that determines the temperature field, and (ii) the etching rate, which depends only on the resulting local temperature.

Specifically, the imprecise wording has been removed, and the text has been revised to state explicitly that Eq. (3) describes the relative change in etching rate as a function of local temperature with respect to a reference temperature (20 °C), independent of how that temperature is generated. We appreciate the reviewer's comment, which helped improve the accuracy and clarity of the description.

Reviewer comment 11: pag 5: "A steeper gradient slope, resulting from the exponential dependence on temperature (Fig. 1e), proves its effectiveness in forming rounded structures." The steeper gradient can be leveraged to get those structures, but cannot "prove its effectiveness"

Author's response: We agree with the reviewer that the original phrasing was overly assertive. A steeper etching-rate gradient alone does not *prove* effectiveness, but rather provides a physical mechanism that can be exploited to achieve controlled geometry evolution.

To address this, we have removed the ambiguous wording and revised the manuscript to avoid causal overstatement. Instead, we now explicitly demonstrate effectiveness through numerical modeling and experimental validation. In the revised manuscript, the role of the nonlinearly decaying, apex-localized etching-rate gradient is quantitatively evaluated using the physics-separated multiphysics framework, which directly reproduces the observed geometry evolution and the formation of aspheric tip profiles spanning multiple conic families.

Thus, rather than inferring effectiveness from the gradient shape alone, the revised manuscript shows how such gradients *enable* controlled rounding and shape transitions, with effectiveness established by agreement between simulation and experiment. We thank the reviewer for prompting this clarification, which improved both the rigor and precision of the presentation.

Reviewer comment 12: pag 5: "After the LCWCE process, the sharp tip of the tapered fiber undergoes etching, yielding a smoothly rounded fiber end." This description is not clear enough at this point in the manuscript, and needs rephrasing. I seem to interpret that the etching process proceeds up to a certain point with the 1550nm laser on ("LCWCE process"), and then at some point the laser is tuned off and the etching continues without its influence? Is that correct?

Author's response: We thank the reviewer for pointing out this ambiguity. The reviewer's interpretation is not correct, and the original wording was indeed insufficiently precise.

In LCWCE, etching and rounding occur exclusively while the laser is on. The localized temperature gradient required for shape evolution is generated by laser absorption in the etchant, and when the laser is turned off, this gradient disappears. The fiber is removed from the etchant immediately after laser illumination is terminated, and no post-etching or laser-free etching step is involved.

The original sentence was intended only to describe the *observable outcome*, that a sharp, chemically tapered fiber tip becomes smoothly rounded *as a result of* the LCWCE process, not to imply a sequential or two-stage etching procedure.

To eliminate this confusion, we have revised the manuscript and Supplementary information to explicitly state that the geometry evolution occurs continuously during laser illumination and ceases when the laser is turned off.

- ***Revised Manuscript (page 5, lines 15-17):*** *"The evolution of the fiber tip geometry with etching time was experimentally characterized under continuous laser illumination (150 mW) in 10.9 wt% HF using high-magnification optical microscopy and scanning electron microscopy (SEM) (**Figs. 1b,c**)."*

- ***Supplementary information (page 4, lines 14-26):*** *"For MLF fabrication, chemically pre-tapered fibers were immersed in a 10.9 wt% HF solution and subjected to continuous laser illumination throughout the etching process. The laser remained on continuously for the entire etching duration, during which the fiber-tip geometry evolved under laser-induced temperature gradients. Immediately upon completion of the LCWCE process, the laser was turned off, and the fiber was removed from the etchant, thereby terminating the etching reaction. The fiber was then rinsed with deionized water to remove residual HF. No further etching occurred after laser shutdown. Prior to immersion, the laser output was stabilized in air for at least 15 minutes to ensure consistent optical power delivery during the shaping process."*

In addition, the revised manuscript now explains the mechanism using numerical simulations that directly link laser-induced heating to time-dependent geometry evolution, further clarifying the process. We believe these revisions fully resolve the ambiguity and improve the clarity of the description.

Reviewer comment 13: pag 5: "effectively confining the incident light at the fiber tip (Figs. 2c,d).". "Confining the incident light" is not what I see in Fig2. I only see a focusing output light from the etched fiber (in fig 2d)

Author's response: We thank the reviewer for pointing out this ambiguity. The original phrasing was imprecise and could indeed be misinterpreted.

Our intention was not to suggest confinement of the *incident (input) light within the fiber*, but rather to describe the spatial confinement and focusing of the emitted light at the fiber output produced by the etched tip geometry. Specifically, Fig. 1c in the revised manuscript and the associated beam-width analysis illustrate the ability of the etched fiber tip to focus the output light into a tightly confined spot in the surrounding medium.

To avoid confusion between input light guidance and output beam shaping, we have removed the phrase "confining the incident light" from the revised manuscript and replaced it with language that explicitly refers to focusing and spatial confinement of the emitted optical field. This revision

ensures consistency with the data shown in Fig. 1c in the revised manuscript and eliminates any potential misinterpretation.

We appreciate the reviewer's comment, which helped improve the clarity and precision of the manuscript.

Reviewer comment 14: pag 5: "[…] marking the first experimental demonstration of subwavelength beam confinement (beamwidth: 406 nm) of lensed fiber in water." What do the authors mean with "first experimental demonstration"? "First" with respect to what? The authors should provide more context for that claim

Author's response: We thank the reviewer for raising this important point. Our original intent in using the term "first experimental demonstration" was to highlight that, to the best of our knowledge, previous studies of lensed fibers have not experimentally measured subwavelength output beam widths in aqueous environments. While several reports have characterized focused beam profiles from lensed fibers in air, where spherical lenses readily provide strong focusing, corresponding experimental measurements in water are rare. In aqueous media, the reduced refractive-index contrast substantially weakens focusing for spherical tips, making aspheric geometries necessary, and prior studies have typically addressed this regime through simulations rather than direct experimental beam-width measurements.

However, we agree that the use of the word "first" without extensive contextualization could be interpreted as overstated or ambiguous. To avoid unnecessary debate over priority and to maintain a conservative and precise presentation, we have removed this claim entirely in the revised manuscript. The revised text now focuses on the demonstrated beam confinement enabled by the precisely engineered tip geometries, without asserting precedence.

This revision aligns the manuscript more closely with its central contribution, predictable and reproducible fabrication of aspheric microlensed fiber geometries, rather than emphasizing claims of priority. We appreciate the reviewer's comment, which helped us refine both the clarity and tone of the presentation.

Reviewer comment 15: pag 5: in the discussion about the evolution of the etching process with consequent different shaping of the fiber tip, the used/needed optical power is only mentioned at the end of the section, while it would be better to say it before.

Author's response: We thank the reviewer for this suggestion. We agree that the optical power should be specified earlier to provide proper experimental context. The manuscript has been revised accordingly. The discussion of geometry evolution now begins with:

Page 5, lines 15–17: The evolution of the fiber tip geometry with etching time was experimentally characterized under continuous laser illumination (150 mW) in 10.9 wt% HF using high-magnification optical microscopy and scanning electron microscopy (SEM) (Figs. 1b,c)."

Reviewer comment 16: pag 5-6: "Here, b was fixed at 5 μm, corresponding to a single-mode

fiber with a mode field diameter of 10 µm (@ 1500 nm).” It is not clear to me why this 5 um should correspond to a SM fiber with a 10um MFD.

Author’s response: We thank the reviewer for pointing out this ambiguity. We agree that the original phrasing did not clearly define the relationship between *b* and the mode-field diameter (MFD).

In the revised manuscript, *b* is explicitly defined as the mode-field radius, i.e., half of the mode-field diameter. For a standard single-mode fiber operating near 1550 nm (e.g., SMF-28–type fibers), the MFD is approximately 10 µm, corresponding to a mode-field radius of $b \approx 5$ µm. This definition follows standard fiber-optics conventions, where the fundamental mode is approximated by a Gaussian profile and the MFD is defined at the $1/e^2$ intensity contour.

We have revised the text to clarify this definition and to emphasize that *b* is used as a normalization length scale for comparing axial sag and curvature, rather than as a strict material or fiber-specific parameter. This clarification resolves the ambiguity and improves consistency with standard fiber terminology.

Reviewer comment 17: pag 6: “As etching proceeds, tip curvature decreases with time, […]”. Is this the etching process that happens AFTER turning the 1550nm laser off? It is currently not clearly described.

Author’s response: We thank the reviewer for pointing out this lack of clarity. The etching process described occurs only while the 1550 nm laser is on. The laser illumination continuously defines the local temperature field and thus the spatially varying etching rate. Once the laser is turned off, the fiber is immediately removed from the etchant, and no further etching or shape evolution occurs.

To avoid any misunderstanding, we have revised the manuscript to explicitly state that the reported decrease in tip curvature with time refers to continuous laser-assisted etching during LCWCE, not to a post-illumination etching process. For more detailed improvement, please refer to our response to comment 12.

Reviewer comment 18: pag 7: “PMLFs fabricated via our all-wet laser-controlled etching process preserve a uniform optical core diameter along the fiber length.“. The proposed process is tapering the fiber to sizes well below the fiber core’s diameter (see Fig. 2), so this statement is hard to interpret at this point.

Author’s response: Thank you for this comment. We agree that the statement required clarification, and the confusion appears to arise from conflating two distinct fiber-tapering approaches: chemical etching and thermal pulling.

Tapered fibers can be fabricated either by chemical etching or by thermal pulling, depending on the fiber type and the intended application. For single-mode fibers, thermal pulling is generally unnecessary, as commercial single-mode fibers already possess a small, well-defined core that supports only the fundamental mode. In this case, microlens fabrication can be performed directly at the fiber tip without modifying the core or cladding diameter along the fiber length.

For multimode fibers, the situation is application dependent. Commercial multimode fibers typically have fixed core diameters, which may or may not be optimal. For applications requiring enhanced spatial confinement or high effective numerical aperture (e.g., biosensing or neural probing), thermal pulling may be used prior to lens fabrication to reduce the core diameter. In contrast, for applications prioritizing efficient light coupling or transmission, such as laser-diode coupling or short-range communication, preserving the original core diameter and fabricating the microlens directly at the fiber tip is preferred.

In this manuscript, we intentionally demonstrate LCWCE on both thermally pulled and non-thermally pulled multimode fibers, as well as on single-mode fibers, to highlight the versatility of the approach. The statement that "PMLFs fabricated via our all-wet laser-controlled etching process preserve a uniform optical core diameter" refers specifically to cases where no thermal pulling is applied prior to lens formation and is intended to contrast LCWCE with melting-based methods that inherently introduce uncontrolled core-diameter transitions.

We have revised the manuscript (**page 12, lines 20 - page 13, line 7 of the revised version**) to clarify this distinction and eliminate potential misinterpretation.

Reviewer comment 19: Page 7: Some of the claims appear to be overstated. For example, although the proposed method may offer improved shape control compared to certain existing techniques, it cannot be considered the most accurate overall. In contrast, two-photon lithography (TPL) enables full control over all geometrical parameters of a parabolic profile ($y = ax^2 + bx + c$). The authors should clarify which specific parameters their method can actually control and to what degree.

Author's response: We thank the reviewer for this important clarification request and agree that the original wording could be interpreted as overstating the degree of geometric control relative to techniques such as two-photon lithography (TPL). We have revised the manuscript to more precisely define the scope and nature of controllability provided by LCWCE.

TPL indeed offers point-by-point, parametric control over arbitrary three-dimensional geometries and remains unmatched in this respect. Our intent is not to claim superior absolute geometric freedom compared to TPL. Instead, the key contribution of LCWCE lies in deterministic, continuous control over curvature-defined axisymmetric geometries directly in glass fibers, achieved through a fundamentally different and far more scalable mechanism.

In the revised manuscript, we now explicitly quantify the achievable geometries using conic fitting, reporting the radius of curvature, conic constant (K), and goodness-of-fit for each fabricated tip. This analysis demonstrates that LCWCE enables predictable and reproducible access to a continuous range of axisymmetric conic profiles, including hyperbolic ($K < -1$), parabolic ($K \approx -1$), prolate elliptical ($K > -1$), and near-spherical geometries, by tuning a small number of experimentally accessible parameters (primarily etching time and initial taper geometry).

Thus, LCWCE does not provide arbitrary free-form control of all polynomial coefficients (a, b, c) in an abstract sense. Rather, it offers robust control over physically meaningful optical parameters,

such as apex curvature, working distance, and conic class, that directly determine focusing and collection behavior in fiber-based systems. This distinction is now made explicit in the revised text, and claims of “accuracy” have been moderated accordingly.

We have revised the manuscript to:

- Remove language implying universal geometric optimality,
- Clearly distinguish LCWCE from serial, polymer-based lithographic methods such as TPL, and
- Emphasize LCWCE’s strengths in deterministic curvature control, scalability, and material compatibility (glass), rather than unrestricted geometric freedom.

We appreciate the reviewer’s comment, which has helped us refine the claims and more accurately position LCWCE within the landscape of micro- and nano-fabrication techniques.

Reviewer comment 20: pag 9: “The micro-bead was suspended in agar gel, then solidified and cut perpendicular to the probe axis.” This sentence seems to mean that the bead was “solidified” and ”cut”. Is that what the authors wanted to mean?

Author’s response: We thank the reviewer for pointing out this ambiguity. We clarify that the agar gel, not the micro-beads, was solidified and sectioned. The micro-beads were embedded within the agar matrix, and after gel solidification, the agar was sectioned perpendicular to the probe axis. This procedure exposed beads located near the cut surface, enabling controlled axial and lateral scanning while maintaining mechanical stability.

To avoid potential misinterpretation and to accommodate the additional experimental data introduced in the revised manuscript, we have moved the detailed experimental description to the Supplementary Information.

Revised Supplementary Information (page 7, lines 3–7): *“Subsequently, the gel was sectioned perpendicular to the probe axis, exposing micro-beads located near the cut surface. Fluorescence was excited and collected through the MLF while the probe was systematically scanned laterally and axially relative to the immobilized bead (Fig. 7). These measurements were compared with those obtained using a tapered MFCF of similar tip diameter (Fig. 7a,c).”*

Reviewer comment 21: The reported application of fiber-based Optical Tweezers (OT) as a benchmark to demonstrate the significance of the proposed method is not particularly compelling. Fiber OT systems with the geometry presented by the authors inherently feature very short working distances in order to achieve the high numerical aperture required for full 3D optical trapping. Consequently, their applicability is intrinsically limited, as truly contactless trapping can typically be realized only for submicron-sized particles (< 1 μm in diameter). In fact, in Fig. S8, the 7 μm bead shown could well be in physical contact with the fiber tip rather than optically trapped in a contactless configuration.

Author’s response: We thank the reviewer for this thoughtful critique and for highlighting an important distinction between fully contactless trapping and near-field (contact or quasi-contact) trapping in fiber-based optical tweezers.

We agree that single-fiber optical tweezer inherently operate at short working distances in order to generate sufficiently large numerical aperture and near-tip intensity gradients for 3D confinement. As a result, its applicability is typically limited to near-field trapping and extended-distance contactless trapping is not its primary strength. We also agree that, in this regime, the trapping configuration of a large bead (e.g., the 7 µm bead in Fig. S8) may involve physical contact or quasi-contact interaction with the fiber tip.

Importantly, however, near-field/contact-assisted fiber trapping is not a limitation unique to our study, but rather a widely studied and actively exploited operating regime in fiber optical tweezers, particularly when the objective is high trapping efficiency, compactness, and operation in confined environments, rather than long-range manipulation. For example, tapered fiber probes have been used for stable trapping and highly flexible manipulation at the fiber end (Eom et al., Pre. Eng., 2009, Xin et al., Sci. Rep., 2012,), fiber tweezers have enabled controlled manipulation of bacteria under microfluidic flow conditions (Xin et al., Appl. Phys. Lett., 2013), nanofiber evanescent-field traps have achieved nanoparticle trapping within tens of nanometers from the surface (Swaim et al., Appl. Phys. Lett., 2013), and single-fiber platforms have integrated trapping, excitation, and detection for single-bacterium analysis (Xin et al., Small, 2017). Near-field fiber probes have also been used for subwavelength biological interrogation where signal localization occurs only upon close contact with the target interface (Li et al., ACS Nano, 2018).

In our work, the optical tweezers demonstration was intentionally conducted in the near-field/contact-assisted regime, enabled by the deterministic fabrication of a hyperbolic microlens. While fully contactless trapping can be achieved with fiber tips of larger curvature radius (weaker focusing and longer effective working distance), this regime is not unique to LCWCE and can also be reached using conventional approaches such as thermal melting. We therefore focus on the practically relevant near-field regime, where trapping performance critically depends on precise and reproducible control of the tip geometry.

More importantly, the purpose of including the fiber-based optical tweezers comparison is not to claim a universally superior trapping modality, but to benchmark the geometry-driven enhancement in near-field trapping efficiency enabled by LCWCE. Our optical force calculations for 450 nm polystyrene nanoparticles show that the LCWCE-fabricated probes generate substantially stronger axial attraction and transverse confinement than conventional microlensed fibers, enabling subwavelength localization near the tip at reduced optical power. In contrast, conventional microlensed fibers exhibit axial repulsion for submicron particles and only trap above a larger size threshold (Fig. S10).

Crucially, the revised manuscript now includes quantitative geometric characterization confirming that the fabricated tips follow hyperbolic conic geometries with high-quality conic fitting. This provides a direct physical basis for the enhanced trapping: hyperbolic tips produce steeper near-field intensity gradients and stronger axial forces than spherical or tapered tips, thereby improving confinement and promoting stable trapping under low-power operation. In contrast, most prior fiber-tweezer approaches based on thermal reshaping or pulling tend to yield spherical/irregular aspheric tips and do not offer deterministic access to hyperbolic conics with quantitative validation or a direct link between geometry control and trapping performance.

Accordingly, we have revised the manuscript to (i) clearly frame the benchmark as a near-field fiber optical tweezers comparison, (ii) acknowledge the possibility of contact/quasi-contact stabilization for large beads, and (iii) emphasize the central advance: a reproducible, geometry-controlled route to hyperbolic fiber tips that measurably improves near-field trapping efficiency.

Revised manuscript (page 15, lines 18-26): *A wide variety of fiber geometries, including tapered and lensed designs, have been explored to enhance trapping performance. Among these, lens geometries that concentrate optical power into a steep axial intensity gradient near the tip are particularly effective, as trapping efficiency in aqueous environments is governed primarily by the local field gradient rather than absolute intensity. In this context, hyperbolic and near-parabolic profiles are especially attractive because they naturally generate strong, tightly confined gradients with short working distances, which are ideal for stable trapping at low optical power. In this short-working-distance regime, micron-scale particles may enter a contact or quasi-contact state after being localized near the tip, which is a widely used operating mode for fiber-based optical tweezers.*

[1] Eom, Sang In, Yasuhiro Takaya, and Terutake Hayashi. "Novel contact probing method using single fiber optical trapping probe." *Precision engineering* 33.3 (2009): 235-242.
[2] Xin, Hongbao, Rui Xu, and Baojun Li. "Optical trapping, driving and arrangement of particles using a tapered fibre probe." *Scientific reports* 2.1 (2012): 818.
[3] Swaim, Jon D., Joachim Knittel, and Warwick P. Bowen. "Tapered nanofiber trapping of high-refractive-index nanoparticles." *Applied Physics Letters* 103.20 (2013).
[4] Xin, Hongbao, et al. "Optofluidic manipulation of Escherichia coli in a microfluidic channel using an abruptly tapered optical fiber." *Applied Physics Letters* 103.3 (2013).
[5] Xin, Hongbao, et al. "Single Upconversion Nanoparticle–Bacterium Cotrapping for Single-Bacterium Labeling and Analysis." *small* 13.14 (2017): 1603418.
[6] Li, Yuchao, et al. "Living nanospear for near-field optical probing." *ACS nano* 12.11 (2018): 10703-10711.

Reviewer comment 22: Page 13: Most of the discussion section mainly reiterates points already presented earlier in the manuscript, with redundant repetitions and little critical analysis. A substantive discussion is missing - one that addresses the current limitations of the proposed method and outlines possible directions for future improvement. For instance, the authors should comment on the reproducibility of the fabrication process and discuss whether, and how, the fabrication parameters (e.g., laser power, etching time, etc.) need to be adjusted depending on the desired final tip geometry.

Author's response: We thank the reviewer for highlighting the importance of reproducibility and parameter dependence, and we have revised the Discussion to address these points explicitly.

First, we now include a quantitative reproducibility assessment in the revised manuscript (new Fig. S2), where multiple fiber tips were fabricated independently under identical LCWCE conditions. The resulting apex radius of 1.49 ± 0.24 μm (n = 6) demonstrates robust reproducibility of the LCWCE process under the operating conditions used in this study. This directly addresses concerns regarding process reliability and operator dependence, which have historically limited broader adoption of lensed fiber technologies.

Second, regarding fabrication parameters, we clarify that all results reported in this work were obtained using moderate laser power and optimized (low) etchant concentration, chosen to ensure

stable and repeatable geometry evolution. While we do not present a systematic experimental study of high-power or high-concentration regimes, we now explicitly note in the Discussion that aggressive processing conditions aimed at maximizing throughput reduce process stability. In particular, higher optical power or etchant concentration can increase sensitivity to small fluctuations in local absorption, reaction timing, and gas evolution, which may perturb the local thermal field during etching.

Importantly, we emphasize that these considerations represent practical operating constraints rather than intrinsic limitations of the LCWCE mechanism. Under appropriately chosen conditions, LCWCE exhibits strong predictability and reproducibility, as demonstrated by the quantitative analysis included in the revised manuscript.

Finally, we outline future directions to further enhance robustness and functional versatility, including closed-loop laser power control, in situ optical or thermal monitoring during etching, and integration of the multiphysics framework to guide parameter selection and inverse design of target geometries.

Reviewer comment 23:

1) Figure 2: the LITWCE label should be replaced with LCWCE.
2) Does TA stand for “taper angle” in subfigure 2e? If yes, please specify if this the angle at the end of the LCWCE step (is my understanding correct?) and add it to figure/caption.

Author’s response: We thank the reviewer for identifying these points.

1. Terminology correction.

The label “LITWCE” in Figure 2 was a typographical error. We have corrected it to LCWCE and ensured consistent terminology throughout the manuscript.

2. Clarification of TA (taper angle).

Yes, TA denotes the initial taper angle of the chemically tapered fiber prior to the LCWCE step, not the angle after lens formation. To eliminate ambiguity, we have explicitly clarified this in both the figure and the caption.

Caption for Figure 1 in the revised manuscript:

(f) Aspect ratio versus etching time, showing controlled transitions from sharp to spherical tip geometries, governed by the initial taper angle (TA) of the chemically tapered fiber before LCWCE. (g) Representative optical images of MLFs corresponding to the etching stages in (f), grouped by initial TA.

These revisions ensure that the role of the initial taper geometry in determining the subsequent lens evolution is clearly defined.

Response to Reviewer #1

Comment 1 - The quality of the figures is relatively low, and the presentation of the content feels somewhat unfocused. I recommend that the authors further optimize the figures to improve clarity and readability and reorganize the text to present the results and their significance in a more cohesive manner.

Author response: We thank the reviewer for this constructive suggestion and agree that improving figure clarity and strengthening the narrative structure would enhance the accessibility and overall presentation of the manuscript.

To address this concern, we revised both the figure presentation and the organization of the Results section to improve visual clarity and reinforce the logical connection between the central concept, underlying mechanism, and resulting functionality, thereby enhancing the overall focus of the manuscript. Although these revisions involved a considerable reorganization and presentation updates, the experimental data, analysis, and conclusions remain unchanged.

Specifically, Figure 1 was redesigned as a focused proof-of-concept figure to more clearly present the overall workflow and outcome of LCWCE, including (i) localized laser-induced wet chemical etching, (ii) deterministic tip evolution, and (iii) experimentally demonstrated optical functionality enabled by controlled geometry formation.

To improve cohesion and reduce redundancy, the subsequent figures and corresponding text were reorganized into a mechanism-driven narrative. The previous separation between physical characterization and geometry evolution was consolidated, resulting in a clearer progression from: (i) deterministic microlens formation (Fig. 1), to (ii) validation of the underlying optical-thermal-chemical mechanism and associated geometry evolution (Fig. 2), and finally to (iii) taper-angle-dependent access to distinct conic geometries and their interpretation (Fig. 3).

To improve the focus of the main narrative, quantitative validation experiments providing additional characterization of the effective sensing profile, defined by the combined emission and collection efficiencies (original Fig. 5), were moved to the Supplementary Information (Supplementary Fig. 8). These results remain fully retained and continue to support device performance while allowing the main text to more clearly emphasize deterministic geometry formation, mechanistic understanding, and functional demonstration.

The invasiveness evaluation of the two-step tapered MLF (original Fig. 10) was also moved to the Supplementary Information (Supplementary Fig. 17) to maintain the focus of the revised manuscript on the fabrication capability of LCWCE across diverse fiber geometries and configurations. While optical performance validation remains in the main text, the invasiveness evaluation was repositioned as supporting application-specific characterization to further emphasize the broader applicability of the fabrication methodology.

Figure 7a was redesigned to improve visual quality and provide a clearer representation of the experimental setup.

The Results section was reorganized to align the discussion more closely with the progression of experimental observations. The revised structure provides clearer separation between fabrication behavior, geometry evolution, broader applicability across fiber configurations, and representative functional demonstrations.

Overall, these revisions were intended to improve clarity and cohesion while placing greater emphasis on the general fabrication principle and deterministic geometry formation enabled by controlled optical-thermal-chemical coupling.

We thank the reviewer again for this valuable suggestion, which helped improve the clarity, organization, and focus of the manuscript.

Comment 2 - For the neural probing, some recent literatures for high-precision optical neural modulation (single cell or even subcellular precision) were missing, for example Light:Sci. Appl. 13, 258 (2024).

Author response: We thank the reviewer for this valuable suggestion and agree that recent advances in high-precision optical neural interfaces provide important context for positioning the significance and future applicability of microfiber-based neural probes.

To address this comment, we expanded the Results section to include recent demonstrations of highly localized optical neural interrogation and modulation, including deep-brain imaging with subcellular spatial resolution and optical stimulation of individual neuronal compartments (Refs. 67 and 68 page 17, including Light: Sci. Appl. 13, 258 (2024)). These studies highlight the rapid progress toward cellular- and subcellular-scale neural interfacing.

While these studies primarily emphasize achieving increasingly localized optical interaction and control, our work focuses on a complementary challenge: enabling minimally invasive deep-brain optical interfacing through deterministic lens formation on ultrathin two-step tapered fibers to engineer localized optical interactions at depth. Although the objectives and implementation differ, together these advances underscore the growing demand for optical neural interfaces that combine high spatial precision with efficient light delivery and accessibility in deep tissue.

Accordingly, we revised the corresponding paragraph to better position the present study within this broader and evolving research landscape.

Comment 3 - No access to the code.

Author response: We thank the reviewer for pointing this out. The code had been uploaded and deposited to Code Ocean at the time of submission and was intended to be available for access and review.

Response to Reviewer #4

Comment 1 - The proposed method may be potentially useful and of interest to researchers working on optical fiber microstructuring and related applications, but the overall scope and significance still appear more suitable for a specialized optics or photonics journal rather than for a broad multidisciplinary venue.

Author response: We appreciate the reviewer's concern and recognize that the previous version may have emphasized the specific fiber implementation more strongly than the broader methodological contribution. To address this, we revised the manuscript to more explicitly communicate that the central contribution of this work is not the fabrication of lensed optical fibers as a specific device outcome, but the establishment of a deterministic strategy for geometry programming through coupled optical, thermal, and chemical processes.

Specifically, we clarified that localized optical excitation under conduction-dominated microscale heat transfer generates stable and spatially confined temperature gradients that are transformed into amplified etching-rate gradients through the exponential temperature dependence of Arrhenius kinetics, thereby continuously guiding geometry evolution. The revised text further emphasizes that these coupled optical-thermal-chemical interactions can be intentionally exploited to enable predictive control of geometry evolution and rational selection of initial geometries to achieve target optical functionality.

To better communicate this concept, Figure 1 and the associated discussion were revised to more clearly illustrate the governing mechanism and generalized workflow. In addition, the Introduction was expanded to provide a more detailed explanation of conduction-dominated optothermal microheating, localized temperature formation, and the resulting spatially controlled etching kinetics, accompanied by a new schematic illustration summarizing this mechanism.

The presentation of the multiphysics framework was also strengthened to improve mechanistic interpretation and highlight the predictive nature of the methodology. To reinforce this aspect, we revised the description of the model to explicitly include moving-boundary geometry evolution. The revised text now states: "We therefore formulate a physics-separated multiphysics modeling framework, combining (i) full wave-optics electromagnetic simulations, (ii) steady-state heat-transfer calculations, and (iii) temperature-dependent wet-etch kinetics coupled with moving-boundary geometry evolution."

This clarification emphasizes that the framework extends beyond optical and thermal analysis on a fixed geometry by capturing the dynamic coupling among optical energy deposition, temperature distribution, spatially varying reaction kinetics, and evolving boundaries throughout fabrication. By explicitly accounting for geometry evolution, the framework enables predictive simulation of structure formation and supports rational selection of processing conditions and initial geometries to achieve target optical functionality.

Accordingly, the revised manuscript places greater emphasis on the fabrication principle and predictive geometry control enabled by the methodology, while presenting the demonstrated fiber architectures as representative examples of its broader applicability. Because the governing

mechanism relies on controllable coupling among optical energy distribution, thermal transport, reaction kinetics, and geometry evolution, the framework is not inherently restricted to the demonstrated fiber architectures and may be extendable to broader classes of geometry-engineered optical interfaces and functional microstructures.

We thank the reviewer for this valuable comment, which helped us improve the positioning, clarity, and broader context of the manuscript.

Comment 2 - The manuscript still suffers at places from expressions unnecessarily repeated even within newly added text (e.g. "10.9 wt% and a laser power of 150 mW" is repeated at least twice), and the writing is not always as accurate and rigorous as it should be.

Author response: We agree that further refinement of presentation quality was warranted. We carefully reviewed the revised manuscript to remove redundant expressions (including repeated descriptions such as "10.9 wt% and a laser power of 150 mW"), improve conciseness, and strengthen technical precision and consistency throughout the text. (Page 7, line 7-15).

Comment 3 - Reviewer #1, do not appear to have been fully resolved in a sufficiently convincing way.

Author response: We appreciate the reviewer's observation regarding comments raised by Reviewer #1. These comments were carefully addressed in the previous revision through substantial revisions to the manuscript, including additional validation, clarification of the physical interpretation, refinement of terminology and scope, and strengthening of quantitative support.

For the present revision, we revisited those sections and implemented additional refinements where further clarification was beneficial to improve readability, continuity of the arguments, and correspondence between the manuscript and the previous point-by-point responses.

Detailed responses and associated manuscript updates are indicated in response to the additional comments from Reviewer #1.

We thank the reviewer again for this comment, which helped us further improve the clarity and presentation of the manuscript.

Changes to figures made for clarity and conciseness

Figure 1
Figure 1a was redesigned to improve the clarity of the LCWCE concept. The revised figure more clearly illustrates the geometry evolution of a tapered fiber driven by localized heating in the surrounding etchant. The schematic further emphasizes that micrometer-scale localized heating establishes a conduction-dominated thermal transport regime, enabling deterministic and spatially confined etching. Corresponding text revisions associated with this figure update are highlighted in red (page 5, line 11 to page 6, line 23).

Figure 2
Figure 2 was merged with Figure 3 of the original manuscript. This reorganization was made to provide a more focused presentation of the optical-thermal-chemical mechanism governing LCWCE-driven geometry evolution. Corresponding text revisions associated with the figure reorganization and the resulting refinement of the Results flow are highlighted in red. (page 7, line 16 to page 10, line 21)

Figure 3
Original figure 4 was merged with original Figures 1f and 1g and reorganized as Figure 3 in the revised manuscript to provide a more focused and cohesive presentation of the results. Corresponding text revisions associated with the figure reorganization and the resulting refinement of the Results flow are highlighted in red. (page 6, line 13 - page 6, line 23 and page 10, line 22 - page 12, line 28).

Because the simulation was performed using half of the fiber geometry under the assumption of geometric and thermal symmetry, the half taper angle (θ) was originally used in Figures 3e and 3f. For consistency and clarity of labels and symbols across the manuscript, this notation was replaced with the full taper angle (TA, ψ).

Figure 6
The color scale in Figure 6g was updated to correctly represent the time-averaged Poynting vector, (<**S**>). In addition, the label D_t(μm) in Figure 6h was revised to correctly indicate transverse displacement.

Figure 7
Figure 7a was redesigned to improve visual quality and provide a clearer representation of the experimental setup. In addition, an error in the time-scale legend of Figure 7g was corrected. To streamline the figure caption, PMT and signal-processing details were removed where redundant and retained in the Methods section.

Changes to text made for clarity and conciseness

All revised text is highlighted in red or green in the Manuscript_Mark and Supplementary_Information_Mark versions.

Text revisions arising from reviewer comments, figure reorganization, restructuring of the presentation and Results narrative, and additional changes introduced to improve the focus and clarity of the manuscript are highlighted in red.

Editorial revisions made in response to the editor's checklist and to comply with Nature formatting and reporting requirements are highlighted in green. Global consistency updates related to formatting, including abbreviation standardization, clarification of symbols and definitions in figure legends, and notation formatting (for example, vector and scalar conventions), were not individually highlighted where changes were applied systematically and did not affect the scientific content, analyses, interpretation, or conclusions.

Page 1, Line 9-11

The affiliations for the Brain Research Centre and the Departments of Psychiatry & Neuroscience and Anesthesiology & Intensive Care should have been listed separately. Accordingly, we revised the author affiliations and presented them as separate institutional affiliations.

Page 3, lines 12-25

The description of our approach was expanded and clarified to better communicate the significance and broader applicability of the study. No new strategy or mechanism was introduced; instead, the existing mechanistic framework was further elaborated to improve clarity and provide a more detailed explanation of the underlying process.

Page 4, line 20

Following the reorganization of Figure 1 to improve coherence and strengthen the logical progression of the Results section, the section title was revised accordingly to better reflect the focus of the content and improve continuity.

Page 5, line 9 - Page 6, line 5, and Page 6, line 15 – line 23

Following the redesign of Figure 1a and the reorganization of the figures, including integration of original Figures 1f–g with Figure 4 to present taper-angle-dependent evolution of rounded fiber-tip geometries within a unified framework, the corresponding text was revised to improve alignment with the updated figure structure and to enhance the clarity, continuity, and logical flow of the Results section.

In Figure 1a, we introduced the local recession orientation term $\theta_y(s)$, which represents the local surface recession orientation relative to the fiber axis (y). This term was introduced solely as a descriptive geometric parameter to facilitate presentation of the proposed geometry-evolution process and provide intuitive interpretation of the resulting conic-shape evolution. The term does not introduce a new physical model or simulation parameter, but rather provides an explicit representation of the surface-normal recession direction already inherent to the multiphysics simulation framework.

Page 6, line 28

“a level of” was added for clarity and to align with standard scientific terminology.

Page 7, lines 7-15

In response to Reviewer #4’s comments, the paragraph was streamlined to improve clarity and reduce redundancy while preserving the underlying physical interpretation.

Page 7, lines 16-18

Following the merger of original Figures 2 and 3 into a unified mechanism section, the section title and introductory text were revised to improve continuity and better reflect the optical-thermal-chemical framework governing geometry evolution.

Page 7, lines 29

To better communicate the broader methodological contribution of the work, we revised the description of the multiphysics framework to explicitly describe the moving-boundary geometry-evolution process that was already incorporated in the simulation but was not sufficiently emphasized in the original strategy overview, which primarily focused on etching kinetics. This clarification highlights that geometry formation emerges through coupled optical-thermal-chemical interactions together with iterative surface recession during fabrication, improving the conceptual understanding and predictive interpretation of deterministic geometry evolution. No additional simulation components or analyses were introduced.

Page 8, lines 10-21

The subtitle of this section was removed because original Figures 2 and 3 were merged into a unified mechanism section. To improve continuity and reduce fragmentation of the discussion, the corresponding text was integrated accordingly.

Additional thermophysical property information that had previously been detailed only in the Supplementary Information was incorporated into the main text to improve clarity and transparency of the thermal modeling assumptions. Consequently, the supplementary figure order was updated.

Page 9, line 3 - Page 11, line 28

Following the reorganization of Figures 1-4 in response to Reviewer #1’s suggestion to improve the focus of the manuscript, the corresponding text was revised to enhance coherence, clarity, and the logical progression of the Results section. These revisions were limited to presentation and narrative organization; the underlying physical interpretation, experimental data, and scientific conclusions remain unchanged.

Page 10, line 7

The description of the difference in evolution timescale between simulations and experiments was corrected for accuracy. Specifically, the text was revised to clarify that the simulated transition

occurs earlier (originally stated as later) than experimentally observed due to the steady-state thermal approximation, which neglects transient heating of the surrounding etchant before establishment of the steady-state temperature field. This discrepancy affects only the absolute time scale and does not alter the predicted evolution sequence or final geometry.

Page 13, lines 5 - line 25

To place greater focus on deterministic geometry formation and mechanistic understanding, the manuscript was streamlined by reducing application-specific details in the main text. This revision improves focus while better highlighting the broader methodological contribution of the study.

Page 14, lines 13-21

As original Figure 5 was moved to the Supplementary Information (Figure S8), the corresponding text was revised and streamlined to maintain the focus of the revised manuscript on the fabrication capability of LCWCE across diverse fiber geometries and configurations.

Page 15, Lines 17-18

To improve the focus and readability of the manuscript, the paragraph was streamlined to reduce redundancy.

Page 17, Lines 3-6

To improve the focus and readability of the manuscript, the paragraph was streamlined while preserving the key information and intended meaning.

Page 17, lines 8-9

To better emphasize the complementary objective of our approach and clarify the intended application of the fiber design in minimally invasive deep-brain neural interfacing, we revised the title from *"Lens Formation on Ultrathin Two-Step Tapered Fibers Enables Deep, High-Resolution Neural Probing"* to *"Lens Formation on Ultrathin Two-Step Tapered Fibers Enables High-Resolution Neural Probing Deep Within Live Brains."*

Page 17, lines 17-27

The manuscript was expanded to include recent demonstrations of highly localized optical neural interrogation and modulation in response to the reviewer #1's suggestion and to better position the study within recent advances in the field.

Page 18, line 3

"single-cellular precision" was replaced with "single-cell precision" for clarity and consistency with standard terminology.

Page 18, line 18-21

For improved clarity, we expanded the text from “In vivo, 2ST-MLFs were inserted into the brains of anesthetized mice expressing ChR2-EYFP and jRGECO1a. jRGECO1a enabled Ca^{2+} imaging with red-shifted excitation (>561 nm) to minimize ChR2 cross-activation” to “In vivo, 2ST-MLFs were inserted into the brains of anesthetized mice in regions where neurons were transduced with the blue-light-activatable opsin channelrhodopsin (ChR2-EYFP) for optogenetic stimulation and the genetically encoded Ca^{2+} indicator jRGECO1a for activity monitoring. jRGECO1a was selected to allow Ca^{2+} imaging under red-shifted excitation (>561 nm), thereby minimizing cross-activation of ChR2.”

Page 20, line 15

To better communicate the broader methodological contribution of the work, we revised the description of the multiphysics framework to explicitly describe the moving-boundary geometry-evolution process, consistent with the corresponding clarification introduced in the Introduction.

Page 23, line 10

A typographical error in the unit was corrected. Specifically, the tapering speed was corrected from µm/min to µm/sec.

Supplementary Information

1. **Page 1, lines 11-13**

   The affiliations for the Brain Research Centre and the Departments of Psychiatry & Neuroscience and Anesthesiology & Intensive Care should have been listed separately. Accordingly, we revised the author affiliations and presented them as separate institutional affiliations.

2. **Page 2, lines 1-20**

   Additional explanatory text was added following relocation of original Figure 5 to the Supplementary Information.

3. **Supplementary figures**

   Due to figure rearrangement and manuscript revision, the supplementary figure order was updated accordingly. Except for the rearrangement of figures within the Supplementary Information, modifications and figures moved from the main text are summarized below.

   - **Supplementary Figure 4**
     The figure caption was updated to improve clarity and remove redundancy.

   - **Supplementary Figures 8 and 17**
     Original Figures 7 and 10 were moved to the Supplementary Information (Supplementary Figures 8 and 17, respectively) to streamline the main narrative and maintain the focus of the revised manuscript on the mechanism and capabilities of

LCWCE for shaping fiber geometries across diverse fiber configurations, while retaining the supporting characterization data. Corresponding text revisions associated with the figure reorganization and the resulting refinement of the Results flow are highlighted in red.

- **Supplementary Figure 10**
  The image was updated to comply with the journal's copyright and formatting policies.

- **Supplementary Figures 15, 16, 20**
  The presentation of laser operating configuration details was updated to maintain a consistent reporting format between the figure panels and captions. More specifically, these details were moved to the figure captions and removed from the figure panels.

- **Supplementary Figure 17**
  The figure was moved from the main manuscript to the Supplementary Information to improve focus.

Editorial updates to improve consistency and compliance with Nature style guidelines

Methods

Methods section (Pages 21–27) was moved from the Supplementary Information to the main manuscript and reorganized into subsections and subheadings in accordance with Nature Portfolio requirements. As a result, references throughout both the main manuscript and Supplementary Information were updated accordingly.

In addition, experimental details and reporting information were expanded in response to the Author Checklist to ensure compliance with Nature Portfolio requirements. These revisions are highlighted in green.

Acknowledgement, Funding, and Contributions

The Acknowledgements, Funding, and Author Contributions sections have been included in the revised manuscript.

Statistics and reproducibility sections.

The optical microscope, SEM, and fluorescence images shown throughout the manuscript are representative images selected to illustrate the corresponding experimental conditions. Accordingly, the term "Representative" has been added to the relevant figure captions where applicable (Figures 1 and 3–7, S5, S7–S9, S12, S14, S15, and S17). Additional information regarding image analysis and reproducibility has been included in the Statistics and Reproducibility section in the Supplementary Information.

Abbreviation standardization and clarification

Abbreviations were reviewed and updated throughout the manuscript following text and figure reorganization. Full terms were introduced at first occurrence where appropriate, and figure legends were revised to improve standalone readability by defining non-standard abbreviations independently within each figure legend. These updates included, but were not limited to, LCWCE, MLF, MFCF, TA, FIB, PMT, and SNR. As these revisions were implemented globally for consistency and compliance with journal style guidelines, individual abbreviation updates were not separately highlighted in the marked manuscript.

Mathematical notation standardization

Mathematical notation was reviewed and standardized throughout the manuscript to ensure consistency with journal style conventions. Vector quantities (for example, **F**, **E**, **H**, **T**, and <**S**>) were formatted in bold, while scalar quantities, vector components, and associated mathematical symbols were formatted according to standard notation conventions

References update

References for the values in Table 1 were included directly in the table in addition to the citations provided in the main manuscript.
